# GbWAKL20 Phosphorylates GbNFYB8 to Modulate Verticillium Wilt Resistance in Cotton

**DOI:** 10.1002/advs.202515724

**Published:** 2026-01-11

**Authors:** Guilin Wang, Qingxin Si, Zhiguo Chen, Zhe Yu, Zhan Guo, Lu Wang, Weixi Li, Wangzhen Guo

**Affiliations:** ^1^ State Key Laboratory of Crop Genetics & Germplasm Enhancement and Utilization Engineering Research Center of Ministry of Education for Cotton Germplasm Enhancement and Application Nanjing Agricultural University Nanjing China

**Keywords:** cotton, GbNFYB8, signaling pathways, verticillium wilt, wall‐associated receptor‐like kinases

## Abstract

Verticillium wilt (VW), caused by *Verticillium dahliae* (*Vd*), is a major threat to cotton production worldwide. Wall‐associated receptor‐like kinases (WAKLs) are critical for plant‐environment communication and fungal pathogen resistance, yet their regulatory mechanisms in cotton remain unclear. Through genomic and transcriptomic analysis, coupled with disease resistance assays, we identified a WAKL gene from *Gossypium barbadense* acc. Hai7124, named *GbWAKL20*, was involved in VW resistance. *GbWAKL20* was significantly induced upon *Vd* infection. Silencing *GbWAKL20* in Hai7124 compromised VW resistance, suppressed the mitogen‐activated protein kinase (MAPK) cascade and salicylic acid (SA) signaling pathway, whereas its ectopic overexpression in Arabidopsis enhanced immune responses. Upon sensing extracellular stress signals at the plasma membrane, GbWAKL20 accumulates and transmits these signals to the nucleus via endoplasmic reticulum‐mediated Golgi vesicle transport. GbWAKL20 interacts with the transcription factor GbNFYB8 and promotes its nuclear translocation through phosphorylation. GbNFYB8 binds to CCAAT elements in promoters of immunity‐related genes and activates their expression. Silencing *GbNFYB8* reduces VW resistance in cotton. GbWAKL20‐mediated phosphorylation enhances transcriptional activation activity of GbNFYB8, further amplifying disease resistance responses and improving plant resistance. Our results highlight the significance of the GbWAKL20‐GbNFYB8 module in defending against VW and provide novel insights into plant immune signaling pathways.

## Graphical abstract

Wall‐associated receptor‐like kinases (WAKLs) play pivotal roles in extracellular–intracellular signal transduction. Upon sensing *Verticillium dahliae* infestation at the plasma membrane, GbWAKL20 accumulates and transmits signals to the nucleus via endoplasmic reticulum‐mediated Golgi vesicle transport. GbWAKL20 phosphorylates the transcription factor GbNFYB8 and promotes its nuclear translocation, greatly activating downstream defense responses and enhancing cotton resistance to *V. dahliae*.

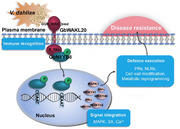

## 1 Introduction

Plants perceive exogenous signals and initiate immune responses through a variety of plasma membrane‐bound receptors [1]. These cell surface receptors include receptor‐like kinases (RLKs) and receptor‐like proteins (RLPs), which serve as an early warning system by recognizing pathogen‐associated molecular patterns (PAMPs) and transmitting signals to downstream molecular networks, thus being referred to as pattern recognition receptors (PRRs) [2]. A typical RLK consists of an extracellular domain, a single transmembrane domain, and a cytoplasmic kinase domain, whereas RLPs lack the kinase domain and possess a short cytoplasmic tail. The extracellular domains of RLKs and RLPs are responsible for ligand recognition, while the cytoplasmic kinase domains of RLKs facilitate signal transduction to the intracellular through phosphorylation events, ultimately eliciting specific cellular responses [3, 4]. PRRs can be classified based on the diversity of their extracellular domains into several groups, including leucine‐rich repeats (LRR), lysin motifs (LysM), lectin motifs, and epidermal growth factor‐like (EGF‐like) motifs [5, 6]. For instance, the LRR receptor kinase FLS2 recognizes the conserved 22‐amino‐acid peptide Flg22 from bacterial flagellin, and forms a complex with the co‐receptor kinase BAK1 to activate immune responses [7]. Chitin binds to LysM receptor kinases CERK1 and LYK4/5, forming a heteromeric complex that mediates signal perception and transmission [8].

Wall‐associated receptor kinases (WAKs) are transmembrane proteins characterized by an intracellular serine/threonine kinase domain, an extracellular calcium‐binding EGF‐like domain, and a galacturonan‐binding (GUB_WAK_bind) domain, which interact with extracellular stress signals and transmit them intracellularly. The first five homologous WAK genes (WAK1‐5) were identified in Arabidopsis, where they form covalent bonds with pectin in the cell wall [9, 10, 11]. In contrast to WAKs, some homologous genes lack or have incomplete GUB_WAK_bind or EGF domains and are collectively referred to as WAK‐like genes (WAKLs) [12]. Although the intracellular kinase domains of WAKs/WAKLs are highly conserved, their extracellular domains exhibit considerable variability, enabling the recognition of diverse ligands [13]. WAKs/WAKLs are primarily localized to the plasma membrane but are closely associated with the extracellular matrix [14]. In Arabidopsis, 5 WAKs and 22 WAKLs have been identified, and many WAKs/WAKLs have also been reported in other plants, including *Oryza sativa*, *Zea mays*, and *Populus trichocarpa* [15, 16, 17]. These WAKs/WAKLs play critical roles in plant cell development, responses to biotic and abiotic stresses, and interactions with the external environment.

One of the most extensively studied functions of WAKs/WAKLs is their regulation of plant defense responses against a wide range of pathogens [18]. In Arabidopsis, rice, and maize, WAKs/WAKLs are highly expressed in vascular tissues under external stress conditions [19, 20]. AtWAK1 forms a complex with the glycine‐rich extracellular protein AtGRP3 and the cytoplasmic type 2C protein phosphatase KAPP to transmit signals. Its expression is induced by salicylic acid (SA) in an NPR1‐dependent manner, and overexpression of *AtWAK1* enhances resistance to *Botrytis cinerea* [21, 22]. In tomatoes, SlWAK1 interacts with FLS2/3 proteins to regulate PRR‐mediated immune responses [23]. In rice, OsWAK14, OsWAK91, and OsWAK92 positively regulate defense responses against rice blast disease [24]. In maize, ZmWAKL interacts with the immunity protein ZmWIK on the plasma membrane, phosphorylates the cytoplasmic kinase ZmBLK1, and enhances plant resistance to gray leaf spot by inducing reactive oxygen species (ROS) accumulation [25]. The *Htn1* gene, encoding a putative wall‐associated receptor‐like kinase, confers resistance to maize leaf blight [26]. Additionally, the *ZmWAK17*‐overexpressing transgenic maize showed enhanced resistance to *Fusarium graminearum* [27]. In *Brassica rapa*, *BrWAK1* is induced by salicylic acid and BrWAK1 interacts with BrBAK1 to activate downstream mitogen‐activated protein kinase (MAPK) signaling, triggering immune responses [28]. In cotton, GhWAK7A binds to chitin receptors and promotes immune activation [29]. GbWAKL14 interacts with GbPP2C to negatively regulate *G. barbadense* resistance to Fusarium wilt [30]. Collectively, these studies highlight the crucial role of WAKs/WAKLs in plant immunity. Natheless, the mechanisms by which WAKs/WAKLs phosphorylate downstream target genes through the kinase domain and contribute to disease resistance remain poorly understood.

*Verticillium dahliae* (*Vd*) is a hemibiotrophic fungal pathogen that causes wilt disease in more than 200 host species, including important economic crops such as cotton, which can result in significant decreases in crop yields [31]. Due to its ability to persist in soil for extended periods and colonize vascular tissues after root infection, chemical control of VW is often ineffective and environmentally detrimental [32]. Therefore, elucidating the mechanisms of immune activation in plants and identifying essential resistance genes for breeding resistant varieties are of utmost urgency. Investigating how cell surface receptors activate immune responses may provide insights into preventing *Vd* invasion and colonization. Despite advances in understanding WAKs/WAKLs in various plants, the key WAKL genes and their functional mechanisms in conferring resistance to *Vd* remain largely unexplored in cotton.

*Gossypium hirsutum* (*G. hirsutum*) and *G. barbadense* are the two main cultivated cotton species. *G. hirsutum* is widely grown for its higher yield, while *G. barbadense* exhibits excellent disease resistance, making it an ideal model for studying cotton disease resistance mechanisms. In this study, we identified a subset of cotton WAKs/WAKLs members responsive to *Vd* infection. Notably, silencing a WAKL gene in *G. barbadense* acc. Hai7124, named *GbWAKL20*, inhibited the MAPK cascade and SA signaling pathway‐mediated immune response, greatly impairing resistance to *Vd*. Conversely, overexpression of *GbWAKL20* in Arabidopsis enhanced immune pathway activation and increased VW resistance. GbWAKL20 transmits external stress signals from the plasma membrane to the nucleus, where it interacts with the transcription factor GbNFYB8, promotes nuclear translocation of GbNFYB8 through phosphorylation, and thereby regulates the activation of downstream immune responses. Our results provide new insights into the functional roles of cotton WAKL proteins in enhancing pathogen resistance, and offer the strategy of targeting the GbWAKL20‐GbNFYB8 module for VW‐resistant breeding utilization in cotton.

## 2 Results

### 2.1 Silencing *GbWAKL20* Impairs Plant Resistance to *Vd* Infection

To mine the key WAK/WAKL family genes associated with VW resistance in cotton, we conducted a genome‐wide search and validation based on the extracellular GUB_WAK_bind domain, the WAK_assoc domain, the EGF domain, and the intracellular Serine/Threonine kinase domain, further identified 139 WAK/WAKL genes in *G. hirsutum* acc. TM‐1 and 136 in *G. barbadense* acc. Hai7124 (Table S1). Based on the domains, these genes were classified into five classes, with the majority belonging to class II, which is characterized by the presence of both the GUB_WAK_bind and kinase domains (Figure S1a). Among these, five genes exhibited higher expression levels in cotton roots or stems, and were significantly upregulated following *Vd* infection (Figure S1b).

We employed a tobacco rattle virus (TRV)‐based virus‐induced gene silencing (VIGS) system to investigate the functional roles of these five *Vd*‐responsive genes using resistant Hai7124 as a receptor. Two weeks after agroinfiltration with TRV: *GbCLA1*, the cotton leaves exhibited a pronounced photobleaching phenotype, confirming the effectiveness of the VIGS system (Figure S2a). Subsequently, Hai7124 seedlings infiltrated with different constructs were sampled for RNA isolation and real‐time quantitative polymerase chain reactions (RT‐qPCR) analysis. The expression levels of the target WAKL genes were significantly reduced in the silenced plants compared to the mock plants (Figure S2b). Repeated experiments confirmed that silencing each of these five genes resulted in varying degrees of compromised VW resistance in cotton. Among them, silencing *GbWAKL20* (*GB_A10G2636*/ *GB_D10G2591*), a homologous gene with Arabidopsis *AtWAKL20*, showed the most severe impact on VW resistance (Table S2).

**FIGURE 1:**
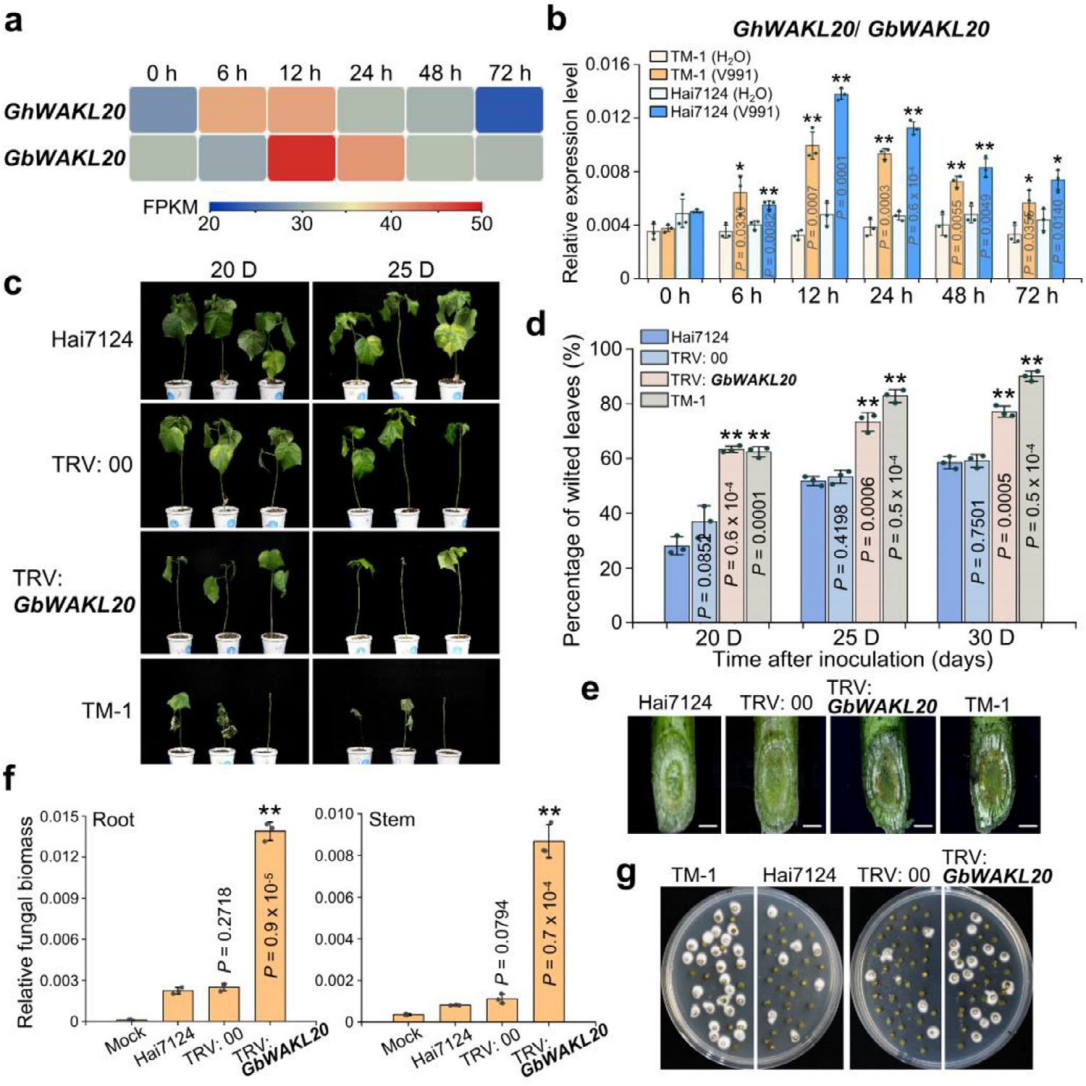
Silencing *GbWAKL20* enhances cotton susceptibility to *Vd* pathogen. (a) The expression pattern of *GhWAKL20*/*GbWAKL20* in the roots of *G. hirsutum* acc. TM‐1 and *G. barbadense* acc. Hai7124 seedlings in response to *Vd* infection were analyzed. The expression data were represented as FPKM values, which were used to calculate the expression levels of *GhWAKL20*/*GbWAKL20*. Colored squares indicated expression levels from 20 (blue) to 50 (red). Each treatment included three biological replicates (*n* = 3). Expression patterns were visualized using TBtools v2.056. Gh, *G. hirsutum*; Gb, *G. barbadense*. (b) RT‐qPCR analysis of *WAKL20* expression in response to *Vd* infection. Error bars represent the standard deviation of three independent biological replicates for each experiment (*n* = 3). The statistical analyses were performed by comparing expression levels at different time points between *Vd* infection and H_2_O treatment using Student's *t*‐test (*P < 0.05, **P < 0.01). (c) Disease symptoms of *GbWAKL20*‐silenced cotton plants were observed at 20 and 25 days after *Vd* inoculation. (d) The percentage of wilted leaves in *GbWAKL20*‐silenced cotton plants after *Vd* inoculation. Each biological repeat contains at least 30 seedlings. Error bars represent the standard deviation of three biological replicates (*n* = 3). Statistical analyses were performed by comparing with Hai7124 using Student's *t*‐test (**P < 0.01). (e) Vascular discoloration was observed in *GbWAKL20*‐silenced plants compared to the controls after *Vd* inoculation. Photographs were taken using a stereoscope (Olympus MVX10, Tokyo, Japan) 15 days post‐inoculation. Scale bars: 1.5 mm. (f) qPCR analysis of fungal biomass in *GbWAKL20*‐silenced and control plants. The DNA was extracted from the roots and stems of plants 15 days after *Vd* inoculation. The mock plants were Hai7124 without *Vd* infection. Error bars represent the standard deviation of three biological replicates (*n* = 3). Statistical analyses were performed by comparing with controls using Student's *t*‐test (**P < 0.01). (g) Fungal recovery experiments. Stem sections of *GbWAKL20*‐silenced and control plants at 15 days post‐inoculation were cut and placed on potato dextrose agar plates and incubated at 25°C. Photographs were taken at 3 days after culture.

RNA‐seq and RT‐qPCR analyses demonstrated that either *GhWAKL20* in TM‐1 or *GbWAKL20* in Hai7124 was significantly upregulated after *Vd* infection, with peak expression occurring approximately 12 hours post‐inoculation (Figure 1a,b). The result indicates *WAKL20* homologs from different cotton species are responsive to *Vd* induction. At 25 days post‐inoculation, the percentage of wilted leaves in TRV: 00 control plants was approximately 53.32%, whereas in TRV: *GbWAKL20* plants, the percentage increased to 73.37% (Figure 1c,d). Stereomicroscopic observation of vascular tissues revealed that *GbWAKL20*‐silenced plants exhibited more severe *Vd* colonization compared to control plants (Figure 1e). Fungal biomass assays further confirmed that *Vd* accumulation in stems was significantly higher in *GbWAKL20*‐silenced plants (Figure 1f). A recovery assay to examine the degree of colonization of *Vd* in infected stems also showed that a large number of colonies were present in the stems of TRV: *GbWAKL20* plants compared to TRV: 00 and Hai7124 plants (Figure 1g). These results indicate that *WAKL20* is upregulated in response to *Vd* infection and plays a critical role in conferring resistance to VW in cotton.

### 2.2 GbWAKL20 Homologs From Different Species Show Variable Extracellular Domain but Conserved Intracellular Kinase Domain

**FIGURE 2:**
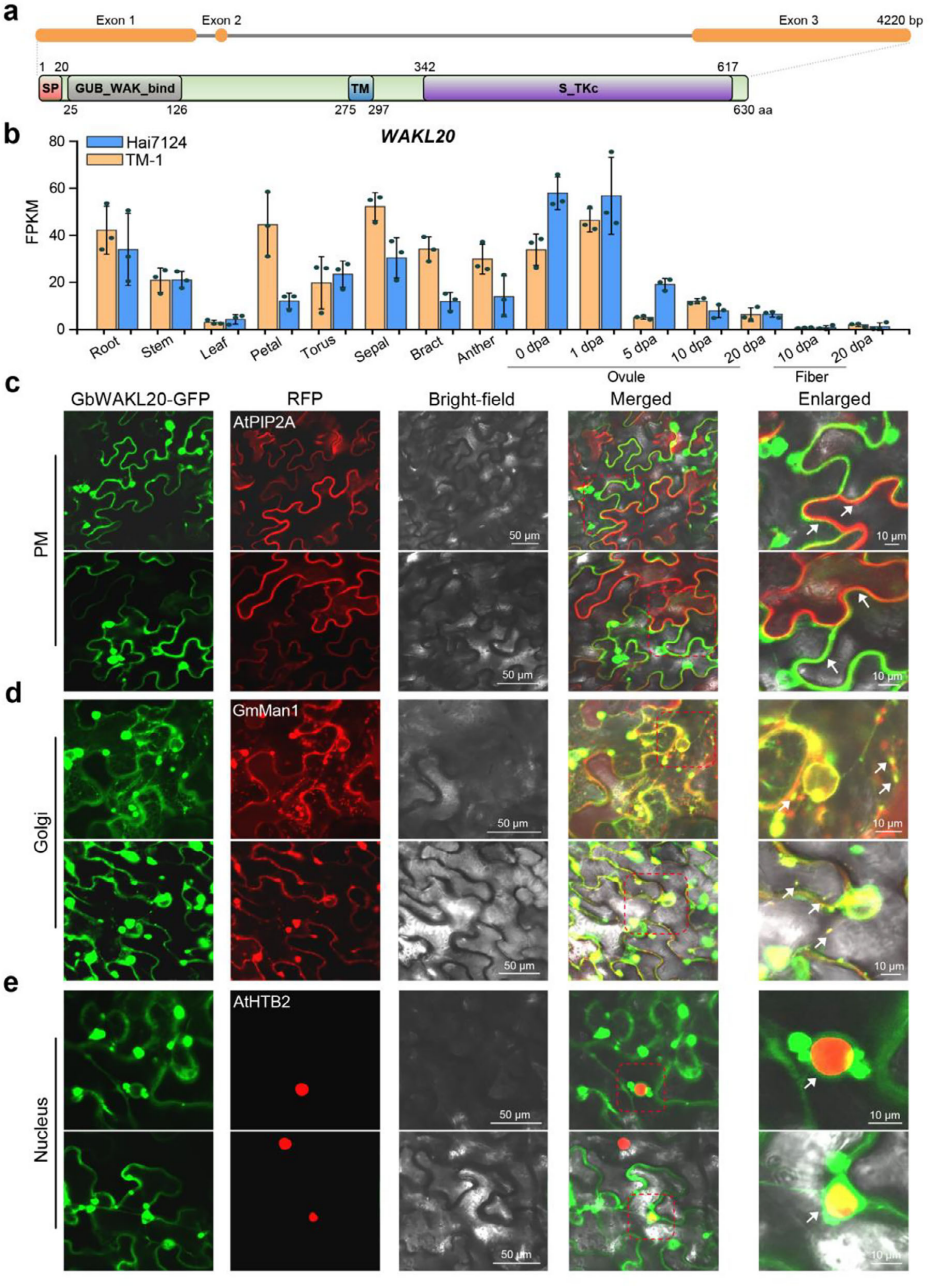
Biological characterization of GbWAKL20. (a) Exon–intron structure analysis and amino acid domains prediction of GbWAKL20 in *G. barbadense* acc. Hai7124. The domains of GbWAKL20 were analyzed using SMART (http://smart.embl.de) and INTERPROSCAN software (http://www.ebi.ac.uk/interpro/). SP, Signal peptide; GUB_WAK_bind, galacturonan‐binding domain; TM, Transmembrane; S_TKc, Serine‐Threonine kinase. aa, amino acid. (b) Expression pattern of *GbWAKL20*/*GhWAKL20* homologs in various tissues in *G. barbadense* acc. Hai7124 and *G. hirsutum* acc. TM‐1. The expression data were converted to FPKM to calculate the expression levels of *GbWAKL20*/*GhWAKL20* in the corresponding species. Error bars represent the standard deviation of three biological replicates (*n *= 3). The RNA‐seq data were obtained from http://www.ncbi.nlm.nih.gov/bioproject/503814. The tissues used include root, stem, leaf, petal, torus, sepal, bract, anther, and ovules at 0, 1, 5, 10, and 20‐days post anthesis (dpa), and fibers at 10 and 20 dpa. (c) The GbWAKL20‐GFP fusion protein co‐localizes with a plasma membrane marker (AtPIP2A, a plasma membrane aquaporin). White arrows indicate overlapping regions of the plasma membrane. (d) The GbWAKL20‐GFP fusion co‐localizes with a Golgi marker (GmMan1, soybean a‐1,2‐mannosidase I). White arrows indicate small overlapping Golgi vesicles. (e) The GbWAKL20‐GFP fusion co‐localizes with a nuclear marker (AtHTB2, histone B2). White arrows indicate GbWAKL20‐GFP green fluorescence distributed around the nucleus. Images were captured by confocal microscopy (LSM 780; Zeiss). Scale bars: 50 and 10 µm.

*GbWAKL20* consists of three exons and two introns. The encoded protein (630 aa) contains a C‐terminal kinase domain, an N‐terminal GUB_WAK_bind domain, a central transmembrane helix, and an N‐terminal 20‐amino‐acid signal peptide (Figure 2a). Expression profiling revealed that *GbWAKL20* in *G. barbadense* acc. Hai7124 and its homologs *GhWAKL20* in *G. hirsutum* acc. TM‐1 exhibited comparable tissue‐specific patterns, with predominant expression in roots, stems, floral organs, and developing ovules, while very low expression in leaves and fibers (Figure 2b). Phylogenetic analyses of WAKL20 orthologs across diverse species revealed a close evolutionary relationship between GhWAKL20/GbWAKL20 and its homologs in *H. syriacus*, *T. cacao*, and *H. umbratica*, both members of the Malvaceae family. In contrast, sequence homology with monocot species (*O. sativa*, *Z. mays*, etc.) was below 60%, and even lower (48.94%) with Arabidopsis (Figure S3). Multiple sequence alignment further highlighted a highly conserved Ser/Thr protein kinase domain, including an ATP‐binding motif and an active site, whereas the signal peptide and the extracellular GUB_WAK_bind domain exhibited obvious divergence (Figure S4). These findings suggest that WAKL20 proteins may recognize distinct extracellular ligands through their variable extracellular domains while activating conserved intracellular signaling pathways via the kinase domain.

### 2.3 GbWAKL20 Localizes to Plasma Membrane and Transmits Signals to Nucleus via Golgi Vesicle Transport

Based on the subcellular localization predictions, GbWAKL20 was shown to be localized to the plasma membrane (Table S3). To further clarify its subcellular distribution, we cloned the full‐length open reading frame (ORF) of *GbWAKL20* from the A‐subgenome in Hai7124 and confirmed the 100% sequence identity with *GB_A10G2636*. We transiently expressed a GbWAKL20‐GFP fusion construct (35S::*GbWAKL20‐GFP*) in *Nicotiana benthamiana* (*N. benthamiana*) epidermal cells by Agrobacterium‐mediated infiltration. Three days post‐infiltration, confocal microscopy revealed that GbWAKL20‐GFP co‐localized with marker proteins of the plasma membrane (PM), Golgi apparatus, and endoplasmic reticulum (ER) (Figure 2c,d and Figure S5a). Intriguingly, GbWAKL20‐GFP fluorescence was also detected at the nuclear membrane, suggesting that the Golgi apparatus contacts the nuclear membrane to transmit intracellular information (Figure 2e). Plasmolysis assays confirmed membrane association, as GbWAKL20‐GFP fluorescence remained PM‐associated without apoplastic or cell wall diffusion (Figure S5b). As a control (35S::*GFP*), GFP fluorescence co‐localizes with ER and nuclear marker proteins, but does not co‐localize with Golgi apparatus marker proteins (Figure S6). Time‐lapse imaging further demonstrated dynamic trafficking of GbWAKL20‐GFP between the PM and nuclear envelope, mediated by Golgi‐derived vesicles (Movies S1–S4). Notably, Golgi bodies frequently clustered near both the PM and nuclear membranes, implying their role in facilitating signal relay. These findings support a model wherein GbWAKL20 functions as a membrane‐anchored sensor, perceiving extracellular stimuli at the PM and transducing signals to the nucleus via the ER‐Golgi network, thereby regulating transcriptional responses to external stress.

### 2.4 Silencing *GbWAKL20* Inhibits MAPK and SA‐Mediated Immune‐Related Pathways

To elucidate the molecular mechanisms by which *GbWAKL20* confers resistance to *Vd* in cotton, we performed RNA‐seq analysis on roots of TRV: *GbWAKL20* plants, with TRV: 00 serving as the empty vector control. Correlation analysis of read counts for 75 071 annotated genes demonstrated high reproducibility among biological replicates, as evidenced by hierarchical clustering (Figure S7a). *GbWAKL20* exhibited significantly lower FPKM (Fragments Per Kilobase of exon model per Million mapped fragments) values in TRV: *GbWAKL20* plants, confirming the effectiveness of gene silencing (Figure S7b). Comparative transcriptome profiling identified a total of 1518 differential expression genes (DEGs) (*q *< 0.05, log_2_FC > 1), comprising 990 downregulated and 528 upregulated genes (Figure S7c). To validate the RNA‐seq data reliability, 17 DEGs were randomly selected for RT‐qPCR analysis, exhibited strong concordance with the sequencing results (*R* ^2^ > 0.8) (Figure S7d).

**FIGURE 3:**
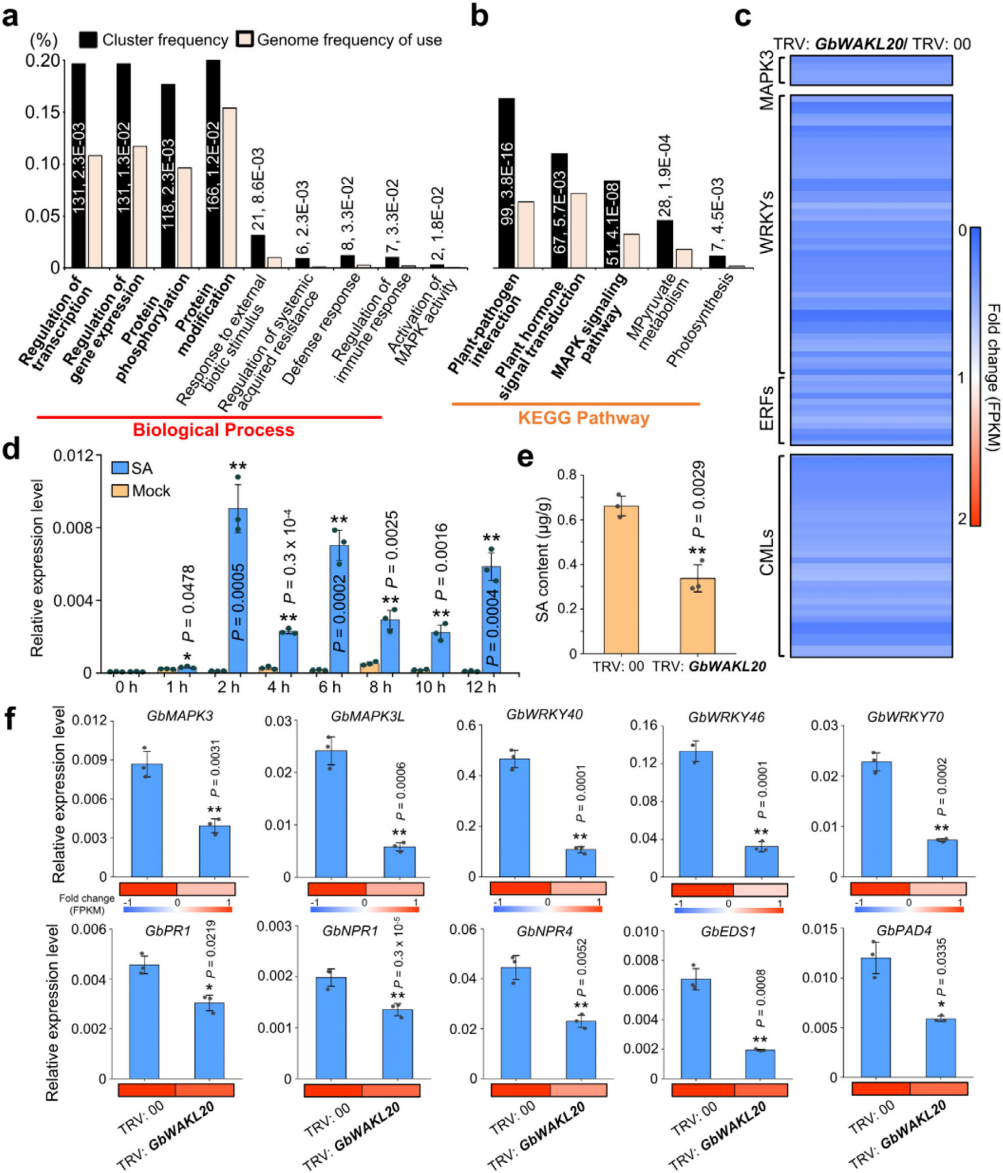
Silencing *GbWAKL20* weakens the systemic immune response in cotton. (a) GO enrichment analysis of biological processes among 1518 differentially expressed genes (DEGs) in TRV: *GbWAKL20* versus TRV: 00 plants. (b) KEGG pathways that were enriched using 1518 DEGs in TRV: *GbWAKL20* plants compared to TRV: 00 plants. The numbers near the columns indicate the count of DEGs with corresponding annotation and the *P.adjust*‐value, respectively. (c) Heatmap illustrating the MAPK cascade pathway‐related genes, hormone‐related transcription factors, and calcium‐binding‐related genes downregulated in TRV: *GbWAKL20* plants compared to TRV: 00 plants. The numerical values on the blue‐to‐red gradient bar represent the fold change of the FPKM values of the DEGs in each sample. (d) The *GbWAKL20* expression level was elevated by SA treatment. Error bars represent the standard deviation of three biological replicates (*n *= 3). (e) Total SA content in TRV: *GbWAKL20* plant roots reduced significantly. Error bars represent the standard deviation of three biological replicates (*n* = 3). (f) The expression of immune‐related genes was validated using RT‐qPCR. Error bars represent the standard deviation of three biological replicates (*n* = 3). Asterisks indicate statistically significant differences, as determined by Student's *t*‐test (*P < 0.05, **P < 0.01). The numerical values on the blue‐to‐red gradient bar represent the fold change of the FPKM values from RNA‐seq analysis.

Gene ontology (GO) enrichment analysis of the DEGs revealed that they were primarily associated with biological processes such as regulation of transcription and gene expression, protein phosphorylation, and protein modification (Figure 3a). In terms of molecular function, DEGs were enriched in DNA binding, transcription factor activity, and protein kinase activity. The effects on cellular components were involved in the membrane (Figure S7e,f). Kyoto Encyclopedia of Genes and Genomes (KEGG) pathway analysis further demonstrated that the DEGs were significantly enriched in immune‐related pathways, including plant–pathogen interaction, plant hormone signal transduction, and the MAPK signaling pathway (Figure 3b and Table S4). Notably, the expression of immune‐related genes, including MAPK3, hormone‐related transcription factors (WRKYs and ERFs), and calcium‐related proteins, was significantly reduced in TRV: *GbWAKL20* plants (Figure 3c and Table S5).

We further analyzed the expression pattern of *GbWAKL20* in cotton treated with signaling molecules such as the immune‐related hormones SA, jasmonic acid (JA), ethylene (ET), and hydrogen peroxide (H_2_O_2_), to clarify its function in the cotton‐*Vd* interaction. *GbWAKL20* expression was significantly upregulated at multiple time points following treatments with SA, JA, ET, or H_2_O_2_, with the most robust activation observed after SA treatment (Figure 3d and Figure S8). Biochemical assays confirmed that total SA content was significantly reduced in *GbWAKL20*‐silenced plants (Figure 3e). Additionally, RT‐qPCR analysis revealed that the expression of *GbMAPK3*, hormone‐related transcription factors (*GbWRKY40*, *GbWRKY46*, and *GbWRKY70*), and SA signaling transduction‐related genes was significantly downregulated in TRV: *GbWAKL20* plants (Figure 3f). These findings suggest that *GbWAKL20*‐mediated cotton immune responses were involved in the MAPK cascade and SA signal transduction pathways.

### 2.5 Overexpressing *GbWAKL20* in Arabidopsis Promotes Activation of Immune Responses

**FIGURE 4:**
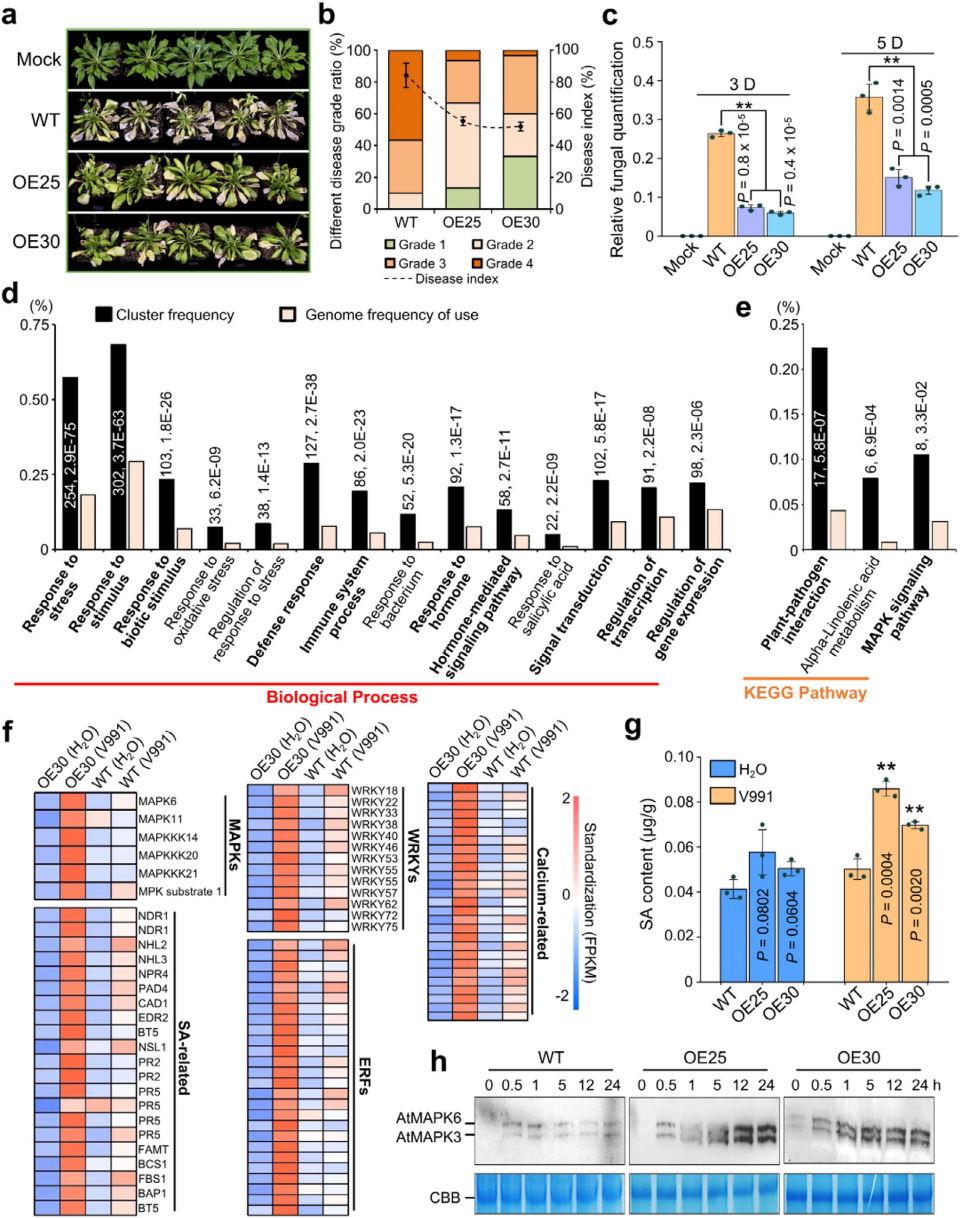
Overexpression of *GbWAKL20* activates immune responses after *Vd* inoculation and improves plant disease resistance. (a) Overexpression of *GbWAKL20* enhances VW resistance in Arabidopsis. Two homozygous transgenic lines, OE25 and OE30, were used for the analysis of disease resistance. The roots of 4‐week‐old Arabidopsis were inoculated with *Vd* spore suspension. Photographs were taken at two weeks after *Vd* inoculation. (b) Statistical analysis of disease grade and disease index in *GbWAKL20* transgenic Arabidopsis and the WT plants. The disease grade was classified into five levels as described in the Experimental Procedures. The data were generated from three biological replicates, each consisting of 30 plants (*n *= 3). (c) qPCR analysis of fungal biomass in *GbWAKL20‐*overexpressing and control plants. The DNA was extracted from the roots of plants at 3 and 5 days post‐inoculation with *Vd*. The mock plants were WT without *Vd* infection. Error bars represent the standard deviation of three biological replicates (*n* = 3). Statistical analyses were performed by comparing with controls using Student's *t*‐test (**P < 0.01). (d,e) GO terms and KEGG pathways were statistically enriched in 62.33% (579/929) of the DEGs with more strongly induced by *Vd* in OE30 plants. The numbers near the columns indicate the number of DEGs with the corresponding annotation and the *P.adjust*‐value, respectively. (f) Heatmap indicating DEGs related to immune response shown in the 62.33% of DEGs. (g) The total SA content in the roots of *GbWAKL20‐*overexpressing plants was significantly increased upon *Vd* infection compared with WT plants. Error bars represent the standard deviation of three biological replicates (*n* = 3). Statistical analyses were performed by comparing with controls using Student's *t*‐test (**P < 0.01). (h) AtMPK3 and AtMPK6 kinase activities in WT, OE25, and OE30 plants after *Vd* treatment. Total proteins loaded were detected by Coomassie Brilliant Blue (CBB) staining.

To further investigate the functional role of *GbWAKL20*, we generated *GbWAKL20*‐overexpressing Arabidopsis lines. Five independent transgenic lines were obtained and validated at both the genomic and transcripts level (Figure S9a,b). When grown under identical conditions for four weeks, no obvious phenotypic differences were observed between *GbWAKL20*‐overexpressing and wild‐type (WT) plants (Figure S9c). Two *GbWAKL20*‐overexpressing lines (OE25 and OE30) exhibiting high *GbWAKL20* expression levels were selected for subsequent *Vd* inoculation assays. Following inoculation with V991, *GbWAKL20*‐overexpressing plants displayed enhanced resistance, as evidenced by attenuated disease symptoms including chlorosis, premature senescence, wilting, and necrosis (Figure 4a). Two weeks post‐infection, the disease index of WT plants reached 83.3%, whereas the disease index for the OE25 and OE30 lines was significantly lower at 56.7% and 52.5%, respectively (Figure 4b). Fungal biomass quantification further confirmed that *Vd* accumulation in the roots of *GbWAKL20*‐overexpressing transgenic plants was significantly reduced compared to WT plants (Figure 4c).

RNA‐seq analysis revealed that the roots of OE30 plants exhibited an enrichment of only 153 DEGs compared to control plants in the absence of *Vd* infection, suggesting that *GbWAKL20* overexpression has minimal impact on Arabidopsis. However, following *Vd* infection, the roots of OE30 plants showed 929 DEGs, with 77% being upregulated. In contrast, WT plants exhibited 1274 DEGs, with a higher proportion of downregulated genes compared to OE30 (Figure S10a–c). These results indicate that *GbWAKL20* overexpression enhances plant disease resistance. Notably, OE30 and WT plants displayed largely non‐overlapping DEG sets post‐infection, indicating that they activate distinct immune responses (Figure S10d). Comparative analysis revealed that 62.33% of *Vd*‐responsive DEGs in OE30 plants showed stronger upregulation than that in WT (Figure S10e). GO and KEGG enrichment analysis showed that these DEGs were associated with immune‐related biological processes, including response to biotic stimulus, response to defense and hormone, and signal transduction (Figure 4d), as well as plant–pathogen interaction and the MAPK signaling pathway (Figure 4e). Furthermore, these DEGs correlate with DNA binding, protein kinase activity, and components of the cell membrane (Figure S10f,g and Table S6). Specifically, DEGs involved in the MAPK cascade, SA signaling, hormone‐related transcription factors (e.g., WRKYs and ERFs), and calcium‐related proteins were significantly upregulated in *GbWAKL20*‐overexpressing plants following *Vd* infection (Figure 4f and Table S7). Consistent with transcriptomic data, *GbWAKL20*‐overexpressing plants accumulated higher SA levels than WT specifically upon *Vd* infection, despite showing no basal SA alteration (Figure 4g). Furthermore, OE25 and OE30 lines exhibited enhanced and sustained activation of AtMPK3/6 within 24 hours post‐inoculation (Figure 4h). These findings suggest that *GbWAKL20* overexpression enhances VW resistance by promoting the activation of MAPK and SA‐mediated defense pathways.

### 2.6 GbWAKL20 Interacts With the Transcription Factor GbNFYB8

**FIGURE 5:**
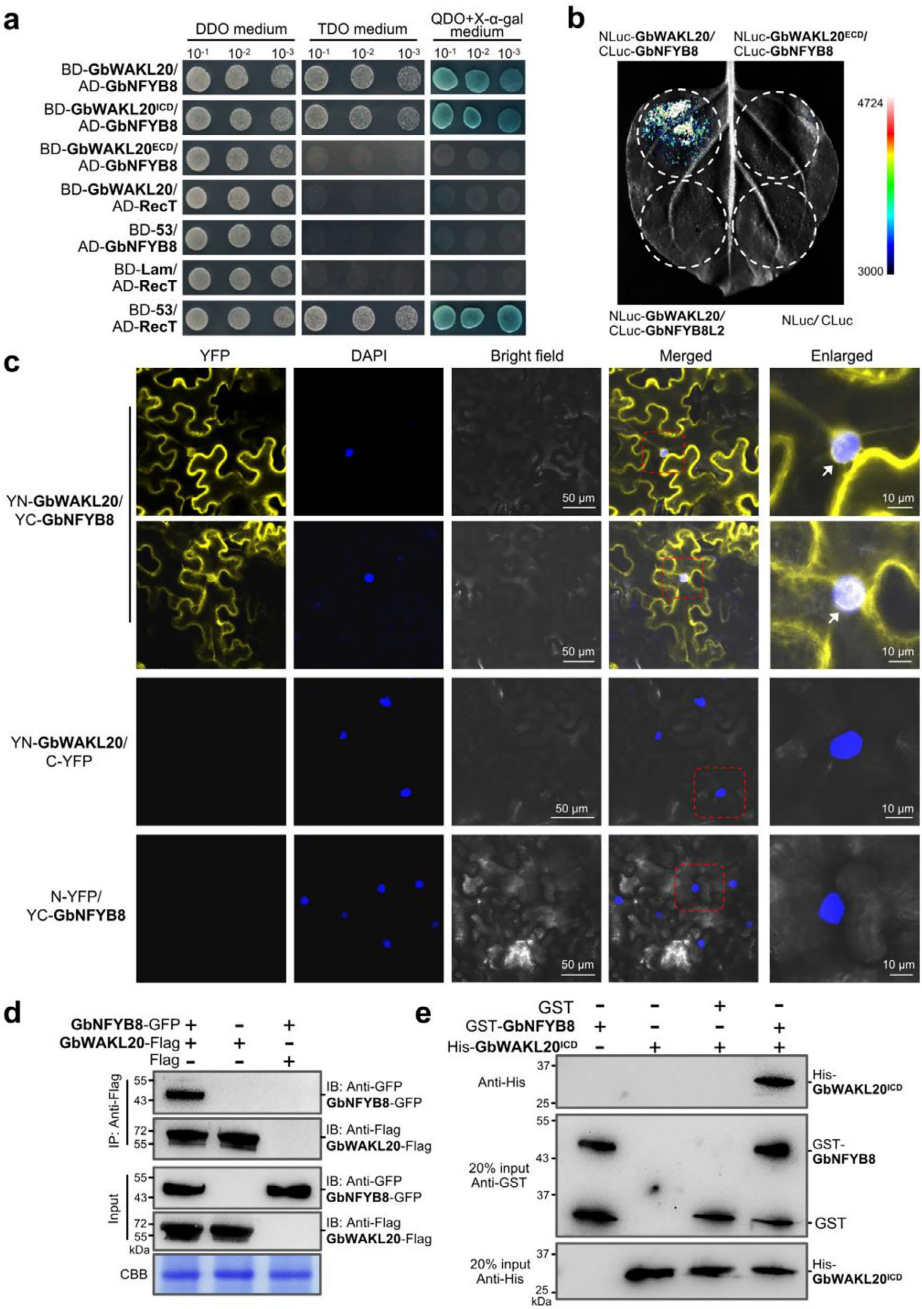
GbWAKL20 interacts with GbNFYB8. (a) GbWAKL20 interaction with GbNFYB8 in yeast‐two‐hybrid (Y2H) assay. Yeast cells containing the indicated plasmids were cultured on selective medium DDO (SD/‐Leu/‐Trp), TDO (SD/‐Leu/‐Trp/‐His), and QDO (SD/‐Leu/‐Trp/‐His/‐Ade) in the presence of X‐α‐gal. Positive interactions can grow and turn blue in QDO medium. Yeast cells with a series of concentrations were dotted on the medium to test the growth rate. GbWAKL20^ICD^, the extracellular domain‐truncated variant spanning residues 126‐630. GbWAKL20^ECD^, the intracellular domain‐truncated variant spanning residues 1‐342. Interactions of BD‐53/AD‐RecT and BD‐Lam/AD‐RecT were used as positive and negative controls, respectively. (b) Verification of the GbWAKL20/GbNFYB8 interaction by luciferase complementation imaging (LCI) assays. The *Agrobacterium* strain GV3101 harboring the indicated plasmid pairs was infiltrated into the leaves of *N. benthamiana* and transiently co‐expressed. The luminescent signal was collected 48 hours post‐infiltration. NLuc‐GbWAKL20^ECD^/GbNFYB8, NLuc‐GbWAKL20/ CLuc‐GbNFYB8L2, and NLuc/CLuc were used as the negative control. GbNFYB8L2, homologous gene of GbNFYB8. (c) Verification of the GbWAKL20/GbNFYB8 interaction by bimolecular fluorescence complementation (BiFC) assays in leaves of *N. benthamiana*. GbWAKL20 was fused to the N‐terminal fragment of YFP (YN‐GbWAKL20), and GbNFYB8 was fused to the C‐terminal fragment of YFP (YC‐GbNFYB8). YN‐GbWAKL20/YC, YN/YC‐GbNFYB8 were used as negative controls. YFP signals indicate an interaction between the two proteins. White arrows indicate that the YFP signals are distributed around the nucleus. The nucleus was stained with DAPI (4’, 6‐diamidino‐2‐phenylindole). Scale bars: 50 and 10 µm. (d) Confirmation of the GbWAKL20/GbNFYB8 interaction by Co‐IP assays. GbWAKL20‐Flag and GbNFYB8‐GFP were co‐expressed in leaves of *N. benthamiana*. Extracted proteins were subjected to Co‐IP using Flag‐trap beads and Western blotting with anti‐Flag and anti‐GFP, respectively. Total proteins stained with CBB served as a loading control. (e) Verification of the GbWAKL20^ICD^/GbNFYB8 interaction by in vitro glutathione S‐transferase (GST) pull‐down assay. GbWAKL20^ICD^ and GbNFYB8 were expressed in *E. coli* as GST or His tag fusion proteins, respectively. Purified GST‐GbNFYB8 or GST protein bound to glutathione sepharose beads was incubated with His‐GbWAKL20^ICD^. The GST‐GbNFYB8 fusion protein was used as bait, and the His‐GbWAKL20^ICD^ fusion protein was used as prey. Eluted proteins were analyzed by immunoblotting with monoclonal anti‐His or anti‐GST antibodies.

To identify proteins interacting with GbWAKL20, we performed a yeast‐two‐hybrid (Y2H) screen using GbWAKL20 as bait against a *G. barbadense* acc. Hai7124 root cDNA library. This screen identified a protein (GB_D11G3806) homologous to Arabidopsis nuclear factor Y, subunit B8 (*NFYB8*, *At2g37060*), designated as GbNFYB8. Y2H analysis demonstrated that GbWAKL20 interacts with GbNFYB8 with the full‐length coding region or the intracellular domain (ICD), but not with the extracellular domain (ECD) (Figure 5a). To further validate this interaction, we conducted luciferase complementation (LCI) and bimolecular fluorescence complementation (BiFC) assays in *N*. *benthamiana* leaves. Luciferase signals were detected only at the infiltration site co‐expressing NLuc‐GbWAKL20 and CLuc‐GbNFYB8, while no signals were observed in negative controls (Figure 5b). BiFC results further verified that the GbWAKL20‐GbNFYB8 complex localizes to the plasma membrane, endoplasmic reticulum, and nucleus. Notably, YFP fluorescence was predominantly observed at the nuclear periphery (Figure 5c). Co‐immunoprecipitation (Co‐IP) assay revealed that Flag‐GbWAKL20, but not Flag alone, precipitated GFP‐GbNFYB8 in *N. benthamiana* leaves (Figure 5d). GST pull‐down assay also demonstrated the interaction between GbNFYB8, expressed as a GST‐tagged fusion, and GbWAKL20^ICD^, expressed as a His‐tagged fusion in *Escherichia coli* (*E. coli*). The GST‐GbNFYB8 successfully pulled down a significant amount of His‐GbWAKL20^ICD^ protein, confirming their physical interaction in vitro (Figure 5e).

To further investigate the specificity of the interaction between GbWAKL20 and GbNFYB8 in cotton, we characterized their respective homologs. *GbWAKL20* possesses three homologs sharing <60% overall sequence identity and <70% identity in the Serine/Threonine kinase domain (Figure S11a–c). Notably, these homologs exhibited low expression levels across cotton tissues, including roots, and showed no response to *Vd* infection (Figure S11d). *GbNFYB8* also has three homologs, sharing 54.29%‐77.53% sequence identity. Additionally, phylogenetic analysis showed that NFYB3/6/7/9, which were clustered in the same clade with NFYB8, had lower homology of 24.62%–53.71% with NFYB8 (Figure S12a,b and Table S8). Although these NFYB proteins had conserved the CBFD_NFYB_HMF domain, their N‐ and C‐terminal regions showed marked divergence (Figure S12c). Thus, we selected the three GbNFYB8 homologs (designated *GbNFYB8L1‐L3*) and four relatively highly expressed *NFYB3* genes (named *GbNFYB3L1‐L4*) to test their interaction with GbWAKL20 (Figure S12d). Results showed that, besides GbNFYB8, only its closest homolog GbNFYB8L1 interacted with GbWAKL20, albeit with weaker interaction strength than GbNFYB8 (Figure S13).

To delineate the structural determinants of the GbNFYB8‐GbWAKL20 interaction, we generated five truncated variants of GbNFYB8 based on the CBFD_NFYB_HMF domain and N‐/C‐terminal regions. The Y2H, LCI, and Co‐IP assays revealed that both the N‐terminal region and CBFD_NFYB_HMF domain were essential for binding to GbWAKL20 (Figure S14). Sequence alignment indicated that GbNFYB8L1, the only homolog exhibiting weak interaction with GbWAKL20, shares higher similarity with GbNFYB8 in these critical regions compared to other NFYB family members. These results demonstrate the strong interaction ability between GbWAKL20 and GbNFYB8, rather than other members of the WAKL and NFYB families.

### 2.7 GbWAKL20 Phosphorylates GbNFYB8 and Promotes Its Nuclear Translocation

**FIGURE 6:**
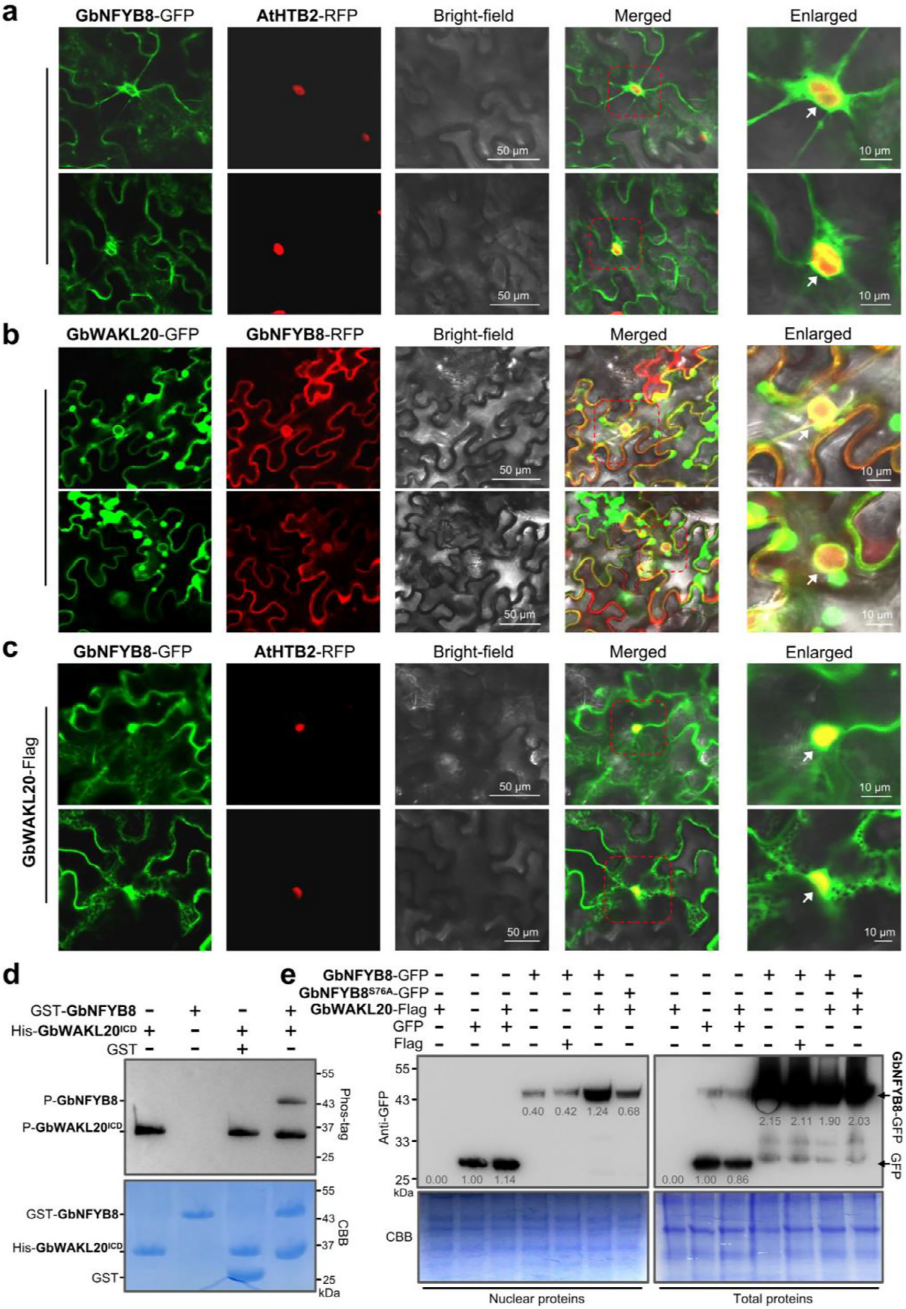
Phosphorylated GbNFYB8 undergoes nuclear translocation. (a) The GbNFYB8‐GFP fusion co‐localizes with a nuclear marker (AtHTB263, histone B2) in *N. benthamiana* leaves. The white arrow indicates that GbNFYB8‐GFP green fluorescence is primarily distributed around the nucleus, with a small amount within the nucleus. (b) Co‐localization of GbWAKL20‐GFP and GbNFYB8‐RFP in *N. benthamiana* leaves. White arrows indicate that GbWAKL20‐GFP fluorescence is distributed around the nucleus, while GbNFYB8‐RFP fluorescence is largely concentrated within the nucleus. (c) Expression of GbWAKL20‐Flag in *N. benthamiana* promotes the translocation of GbNFYB8‐GFP fluorescence into the nucleus. White arrows indicate that the GbNFYB8‐GFP fluorescence overlaps with the nuclear marker. Scale bars: 50 and 10 µm. d) Phosphorylation experiments of GbWAKL20^ICD^ and GbNFYB8 were performed in vitro using biotin‐pendant Zn^2+^ phos‐tag (BTL‐111). CBB is used for protein staining in the phosphorylation assays. (e) Immunodetection of GbNFYB8‐GFP and GFP in crude leaf protein extracts using a GFP antibody. GFP, GbNFYB8‐GFP, and GbNFYB8^S76A^‐GFP were expressed in *N. benthamiana* leaves, and total proteins or nuclear proteins were isolated at 72 hours post‐infiltration. When GbWAKL20‐Flag was co‐expressed, GbNFYB8‐GFP accumulated at high levels in the nucleus. In contrast, GbNFYB8‐GFP expression was minimal in the absence of GbWAKL20 or when the S76 phosphorylation site was mutated. “+” indicates protein expression in the leaves. Total proteins and nuclear proteins loaded were detected by Coomassie Brilliant Blue (CBB) staining. The numbers represent the relative protein level, the amount of GFP in total proteins and nuclear proteins was each set as 1.

To elucidate the functional mechanism of the GbWAKL20‐GbNFYB8 module, we compared the subcellular localization and phosphorylation status of GbNFYB8 with or without GbWAKL20. Subcellular localization indicated that GbNFYB8 localizes to the plasma membrane, endoplasmic reticulum, and nucleus. Similar to the results from the BiFC assay, the GbNFYB8 predominantly accumulated at the nuclear periphery, while low accumulation within the nucleus (Figure 6a and Figure S15a,c). Interestingly, when GbWAKL20‐GFP and GbNFYB8‐RFP were co‐expressed, the GbWAKL20‐GFP remained at the nuclear membrane, while GbNFYB8‐RFP was markedly enriched within the nucleus (Figure 6b). Similarly, co‐expression of the GbWAKL20‐Flag protein with GbNFYB8‐GFP in *N. benthamiana* showed the significantly enhanced accumulation of GbNFYB8‐GFP within the nucleus (Figure 6c and Figure S15b,d).

Phos‐tag phosphorylation assays revealed that GbWAKL20^ICD^ exhibits strong autophosphorylation activity. A distinct phosphorylation band was detected only when GST‐GbNFYB8 was incubated with His‐GbWAKL20^ICD^ and probed with Phos‐tag antibodies, indicating that GbWAKL20^ICD^ possesses kinase activity and phosphorylates GbNFYB8 in vitro (Figure 6d).

Bioinformatic analysis predicted nine high‐probability phosphorylation sites in GbNFYB8, distributed across its N‐terminus (five sites), CBFD domain (one site), and C‐terminus (three sites). Among these, the Serine residue at position 76 (S76) within the CBFD domain is highly conserved (Figure S16a,b). Due to the interaction between the N‐terminal region and the CBFD_NFYB_HMF domain of GbNFYB8 and GbWAKL20, we individually mutated six Serine residues of GbNFYB8 (Ser‐9, −14, −17, −21, −24, and −76) in the regions into Alanine. Co‐IP assays in *N. benthamiana* leaves revealed that GbWAKL20‐Flag phosphorylates GbNFYB8‐GFP, as detected by an anti‐pSer/pThr antibody. Notably, only mutating Ser‐76 to Ala (S76A) in full‐length GbNFYB8 largely blocked its phosphorylation by GbWAKL20, indicating that the Ser‐76 residue in GbNFYB8 is the primary phosphorylation site targeted by GbWAKL20 (Figure S16c).

We extracted the total proteins and nuclear proteins from *N. benthamiana* leaves to further quantify the nuclear accumulation of GbNFYB8. When expressed alone, GbNFYB8‐GFP exhibited low nuclear accumulation, in contrast to the GFP control with high nuclear accumulation. Co‐expression with GbWAKL20‐Flag substantially enhanced the nuclear accumulation of GbNFYB8‐GFP protein, while the nuclear accumulation of the GFP control remained unaffected. This enhanced nuclear accumulation was abolished when Ser‐76 of GbNFYB8‐GFP was mutated to alanine (S76A). Notably, the total protein levels of GbNFYB8‐GFP remained constant with or without GbWAKL20‐Flag or the Ser‐76 site mutated or not in GbNFYB8 (Figure 6e). Consistent with these findings, subcellular localization assays confirmed that the S76A mutation prevented GbWAKL20‐induced nuclear localization of both GbNFYB8‐RFP and GbNFYB8‐GFP (Figure S17). Collectively, these results demonstrate that GbWAKL20 promotes the nuclear translocation of GbNFYB8 by phosphorylating it at Ser‐76, thereby enhancing its transcriptional regulatory activity.

### 2.8 Silencing *GbNFYB8* Inhibits Immune‐Related Pathways in Cotton

**FIGURE 7:**
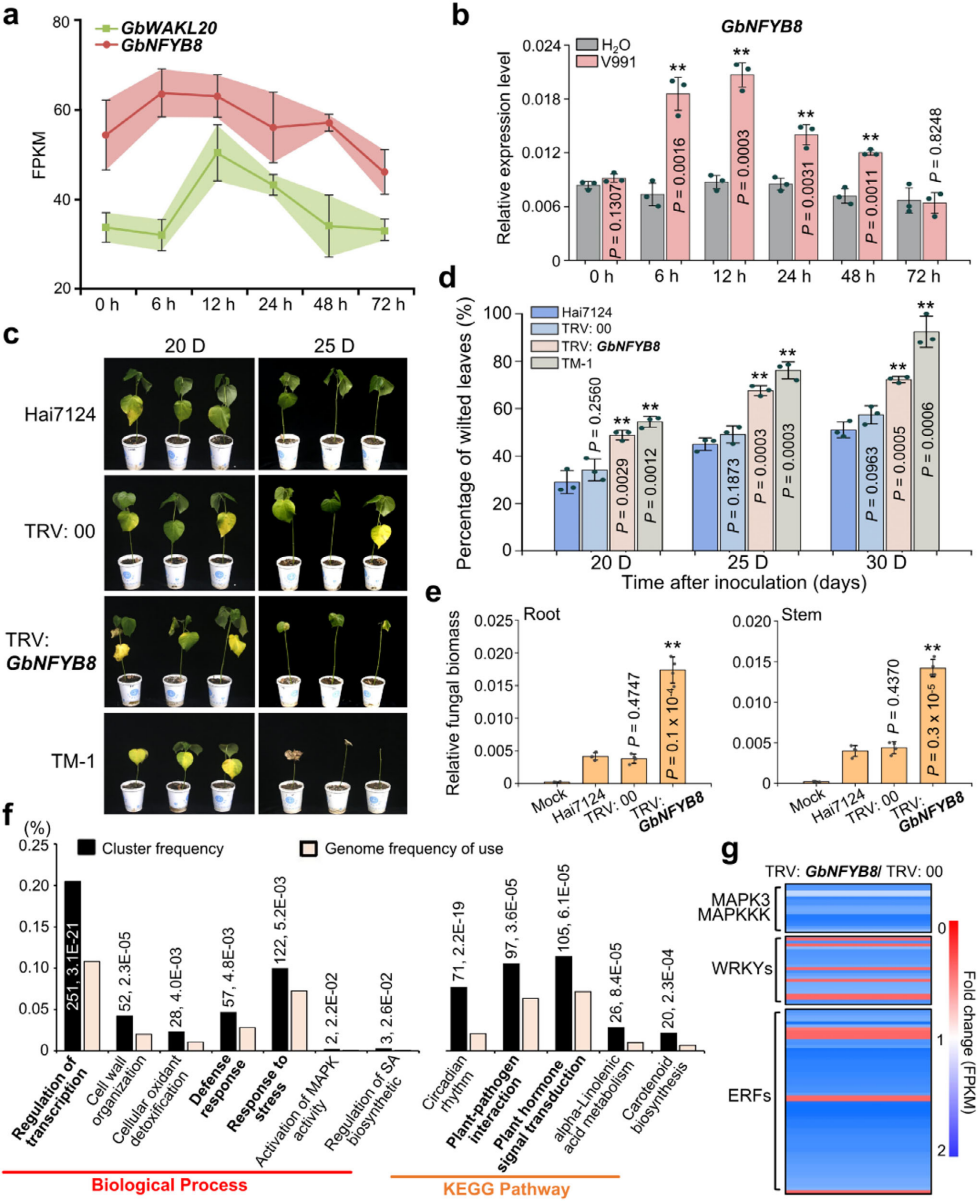
Silencing *GbNFYB8* weakens the systemic immune response and reduces the plant resistance to *Vd*. (a) G*bNFYB8* and *GbWAKL20* expression patterns in roots of Hai7124 seedlings in response to *Vd* infection were analyzed. The expression data were represented as FPKM values, which were used to calculate the expression levels of *GbNFYB8* and *GbWAKL20*. Error bars represent the standard deviation of three biological replicates (*n *= 3). (b) RT‐qPCR analysis of *GbNFYB8* expression in response to *Vd* infection. Error bars represent the standard deviation of three independent biological replicates (*n *= 3). The statistical analyses were performed by comparing expression levels at different time points between *Vd* infection and H_2_O treatment using Student's *t*‐test (**P < 0.01). (c) Disease symptoms of *GbNFYB8*‐silenced cotton plants were observed at 20 and 25 days after *Vd* inoculation. (d) The percentage of wilted leaves in *GbNFYB8*‐silenced cotton plants after *Vd* inoculation. Each biological repeat contains at least 30 seedlings. Error bars represent the standard deviation of three biological replicates (*n* = 3). Statistical analyses were performed by comparing with Hai7124 using Student's *t*‐test (**P < 0.01). (e) qPCR analysis of fungal biomass in *GbNFYB8*‐silenced and control plants. The DNAs of roots and stems were extracted from plants 15 days post‐inoculation. The mock plants used were Hai7124, which were not infected with *Vd*. Error bars represent the standard deviation of four biological replicates (*n* = 4). Statistical analyses were performed by comparing with controls using Student's *t*‐test (**P < 0.01). (f) GO terms for biological processes and KEGG pathways that were statistically enriched in 2342 DEGs in the TRV: *GbNFYB8* plants compared to TRV: 00 plants. The numbers near the columns indicate the count of DEGs with corresponding annotation and the *P.adjust*‐value, respectively. (g) Heatmap showing the MAPK cascade pathway‐related genes and hormone‐related transcription factors in TRV: *GbNFYB8* plants compared to TRV: 00 plants. The numerical values on the blue‐to‐red gradient bar represent the fold change of the FPKM values of the DEGs in the TRV: *GbNFYB8* plants compared to TRV: 00 plants.

NFYB, a highly conserved histone‐like transcription factor, with high specificity and affinity for binding to the CCAAT element in gene promoters [33]. Although extensively studied in cancer biology, its role in plant immunity remains largely unexplored [34, 35]. RNA‐seq and RT‐qPCR analyses showed that *GbNFYB8* and *GbWAKL20* exhibit similar induced expression patterns, showing upregulation in response to *Vd* infection, particularly during the early induction phase (6 to 24 hours post‐inoculation) (Figure 7a,b). In contrast, none of the GbNFYB8 homologs (*GbNFYB8L1‐3*) displayed such *Vd*‐responsive upregulation (Figure S18). We employed the VIGS technique to specifically silence *GbNFYB8* in cotton (Figure S19a). Repeated experiments confirmed that silencing *GbNFYB8* significantly reduced resistance to VW in Hai7124 (Figure 7c). At 25 days post‐inoculation, the percentage of wilted leaves in TRV: 00 control plants was approximately 49.12%, whereas in TRV: *GbNFYB8* plants, the percentage increased to 67.60% (Figure 7d). Microscopic observations of browning in vascular bundles, fungal biomass, and fungal recovery assay further indicated that *GbNFYB8*‐silenced plants exhibited greater fungal accumulation in stems of *GbNFYB8*‐silenced plants (Figure 7e and Figure S19b,c). This finding aligns with the observed effects of *GbWAKL20*‐silenced Hai7124 plants.

We further conducted a comparative transcriptome analysis to examine the DEGs in the roots of *GbNFYB8*‐silenced plants compared to control plants. A total of 2343 DEGs were identified, with *GbNFYB8* expression significantly reduced in TRV: *GbNFYB8* plants, while its homologs *GbNFYB8L1‐L3* remained unchanged (Figure S20a–c). The GO and KEGG enrichment analyses of DEGs in *GbNFYB8*‐silenced plants revealed similarities to those observed in *GbWAKL20*‐silenced plants. The DEGs were enriched in biological processes such as transcription regulation and stress response, pathways including plant–pathogen interaction and plant hormone signal transduction, and molecular functions such as DNA binding and transporter activity. Notably, unlike *GbWAKL20*‐silenced plants, phosphorylation‐related terms were not enriched, and the associated cellular components were primarily nuclear (Figure 7f, Figure S20d, and Table S9). MAPK cascade pathway‐related genes such as *MAPK3*, and hormone‐associated transcription factors (WRKYs and ERFs), were significantly downregulated in TRV: *GbNFYB8* plants, consistent with the results observed after silencing *GbWAKL20* (Figure 7g and Table S10). These results suggest that *GbNFYB8* plays a critical role in transcriptional regulation and defense responses in cotton.

### 2.9 GbWAKL20 Enhances the Transcriptional Activation Activity of GbNFYB8

**FIGURE 8:**
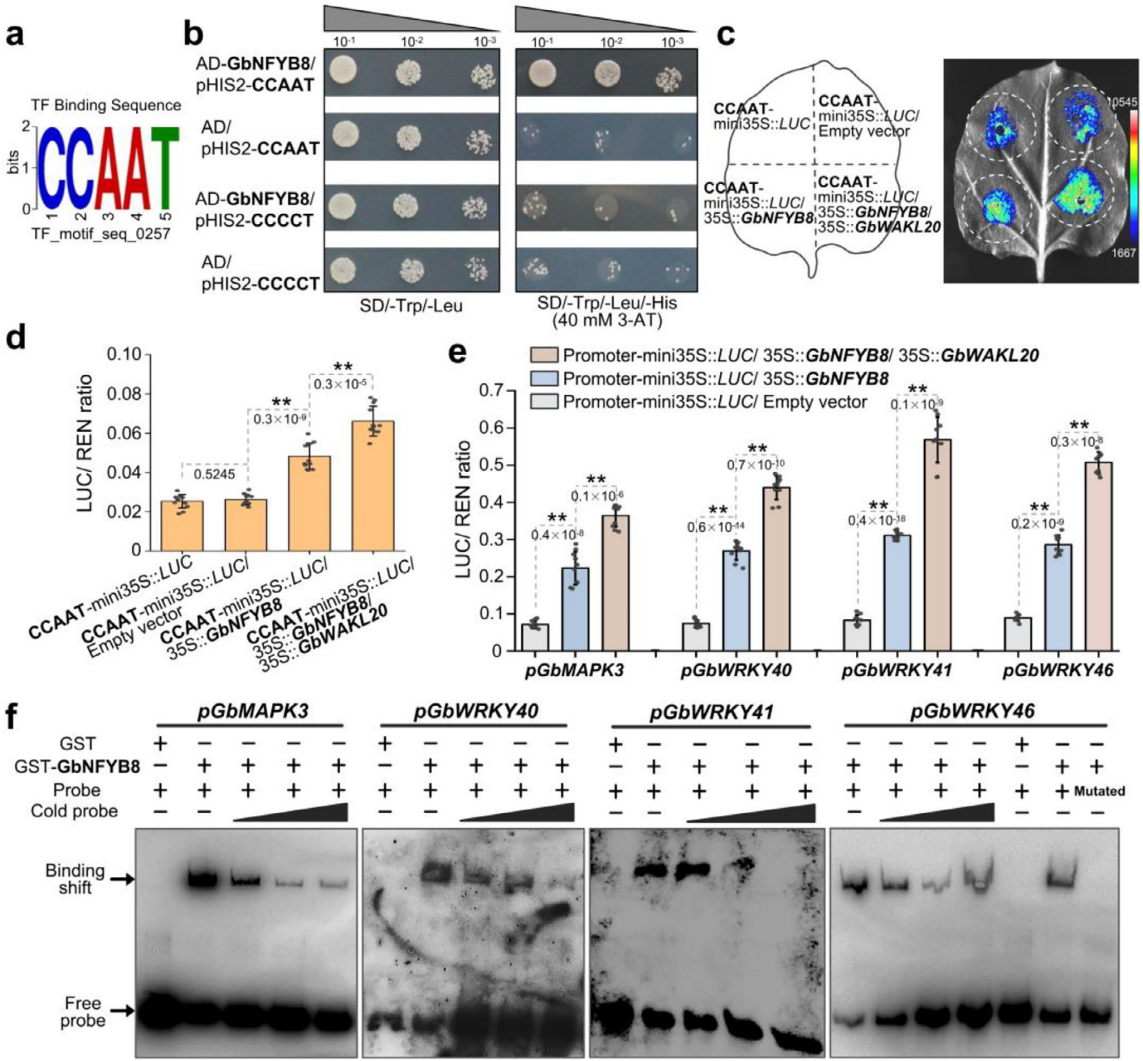
GbWAKL20‐mediated phosphorylation of GbNFYB8 enhances its ability to bind the CCAAT element. (a) Predictions from the PlantPAN 4.0 website (https://plantpan.itps.ncku.edu.tw) indicate that GbNFYB8 could bind to the CCAAT element. (b) The yeast one‐hybrid assay shows that GbNFYB8 binds to the CCAAT element. The CCCCT element with the base mutation is used as the negative control. A quantity of 40 mm 3‐amino‐1,2,4‐triazole (3‐AT) is applied to suppress the basal expression of the bait construct. (c) Transactivation analysis using the LUC reporter system reveals that the GbNFYB8 binds to the CCAAT element and promotes downstream LUC gene expression. Furthermore, GbWAKL20 interacts with the GbNFYB8, promoting LUC accumulation. The mini35S promoter sequence with a CCAAT element was co‐transformed with GbNFYB8 and GbWAKL20, respectively. (d) Dual‐LUC transient expression assay showed that GbNFYB8 enhances the activity of the CCAAT‐mini35S promoter. The activity of the CCAAT‐mini35S promoter was significantly elevated following the additional expression of the GbWAKL20. Data were collected from three biological replicates for each reaction with four technical replicates for each (*n* = 12). (e) The LUC/REN ratio serves as a measure of the promoter activity in a transient expression assay. The LUC activities were normalized to REN. For each experiment. Data were collected from five biological replicates for each reaction with two technical replicates for each (*n* = 10). Asterisks indicate statistically significant differences, as determined by Student's *t*‐tests (**P < 0.01). (f) EMSA results of GbNFYB8 binding with the core sequence in the promoters of target genes. The 55 bp sequence of the *GbMAPK3*, *GbWRKY40*, *GbWRKY41*, and *GbWRKY46* promoters containing the CCAAT element was used as the probe and labeled with biotin. The unlabeled fragment served as a competitor. −, absence; +, presence. The mutated CCAAT element in the *GbWRKY46* promoter was mutated to CCCCT.

Phylogenetic analysis demonstrated that GbNFYB8 possesses a highly conserved CBFD_NFYB_HMF domain in different plants, which is responsible for binding CCAAT elements in gene promoters (Figure S21). Predictive analysis further indicated that GbNFYB8 could recognize and bind to CCAAT elements (Figure 8a). Yeast one‐hybrid assays confirmed that GbNFYB8 binds to the CCAAT element but not to a mutated CCCCT element (Figure 8b). To further investigate the transcriptional activation activity of GbNFYB8, we performed a firefly luciferase (LUC) assay, GbNFYB8 was used as the effector, and the sequence containing 3×tandem repeats of CCAAT‐element fused upstream of mini35S::*LUC* formed the reporters. The *N. benthamiana* leaves infiltrated with CCAAT‐mini35S::*LUC* alone or co‐infiltrated with CCAAT‐mini35S::*LUC* and a native effector exhibited weak fluorescence signals. In contrast, leaves co‐infiltrated with CCAAT‐mini35S::*LUC* and GbNFYB8 effector showed strong fluorescence, which was further enhanced by the addition of GbWAKL20 (Figure 8c). Additionally, we employed a dual‐luciferase reporter system, using the REN gene in the reporter construct as an internal control. The LUC/REN ratio was significantly increased in *N. benthamiana* leaves co‐transformed with the GbNFYB8 effector and CCAAT‐mini35S::*LUC* reporter. Similarly, the inclusion of GbWAKL20 further promoted LUC expression (Figure 8d).

Among the DEGs affected by silencing *GbWAKL20* or *GbNFYB8*, we identified 275 common DEGs, including significantly downregulated genes such as MAPK3 and several WRKY transcription factors. Many of these genes contain CCAAT elements within their upstream 1.5 kb promoter regions (Figure S22a,b). Among them, *GbMAPK3*, *GbWRKY40*, *GbWRKY41*, and *GbWRKY46* exhibited high expression levels in roots and stems, and were significantly upregulated 6–24 hours after *Vd* infection, showing expression patterns similar to *GbWAKL20* and *GbNFYB8* (Figure S22c). The interaction of the promoters of *GbMAPK3*, *GbWRKY40*, *GbWRKY41*, and *GbWRKY46* with GbNFYB8 was investigated using the LUC/REN system, respectively. The LUC/REN ratios exhibited a significant increase in the leaves that transformed with both the GbNFYB8 effector and promoter‐mini35S::*LUC* reporters compared to the control, and the addition of GbWAKL20 further promoted the expression of the *LUC* gene (Figure 8e). Next, electrophoretic mobility shift assays (EMSA) were further conducted to determine whether GbNFYB8 binds to the promoter of the four genes. The DNA probes used in this assay were 55‐bp promoter fragments containing CCAAT elements, which were biotin‐labeled. The results indicated that the recombinant GbNFYB8 protein strongly bound to the biotin‐labeled probe containing the promoters of *GbMAPK3*, *GbWRKY40*, *GbWRKY41*, and *GbWRKY46*, respectively. Furthermore, the addition of unlabeled probes (competitive) significantly reduced the binding. However, when the CCAAT element in the *GbWRKY46* promoter was mutated to CCCCT, it could not bind GbNFYB8 (Figure 8f).

RNA‐seq analysis demonstrated that silencing either *GbWAKL20* or *GbNFYB8* significantly downregulated multiple defense‐related genes, including pathogenesis‐related protein genes, disease resistance genes, cell wall‐related genes, and secondary metabolism‐related genes. In addition, these genes exhibited enhanced induction in *GbWAKL2*0‐overexpressing plants following *Vd* infection compared to WT controls (Figure S23 and Table S11). GbMAPK3 or WRKY transcription factors may ultimately enhance VW resistance by modulating these defense‐related processes.

**FIGURE 9:**
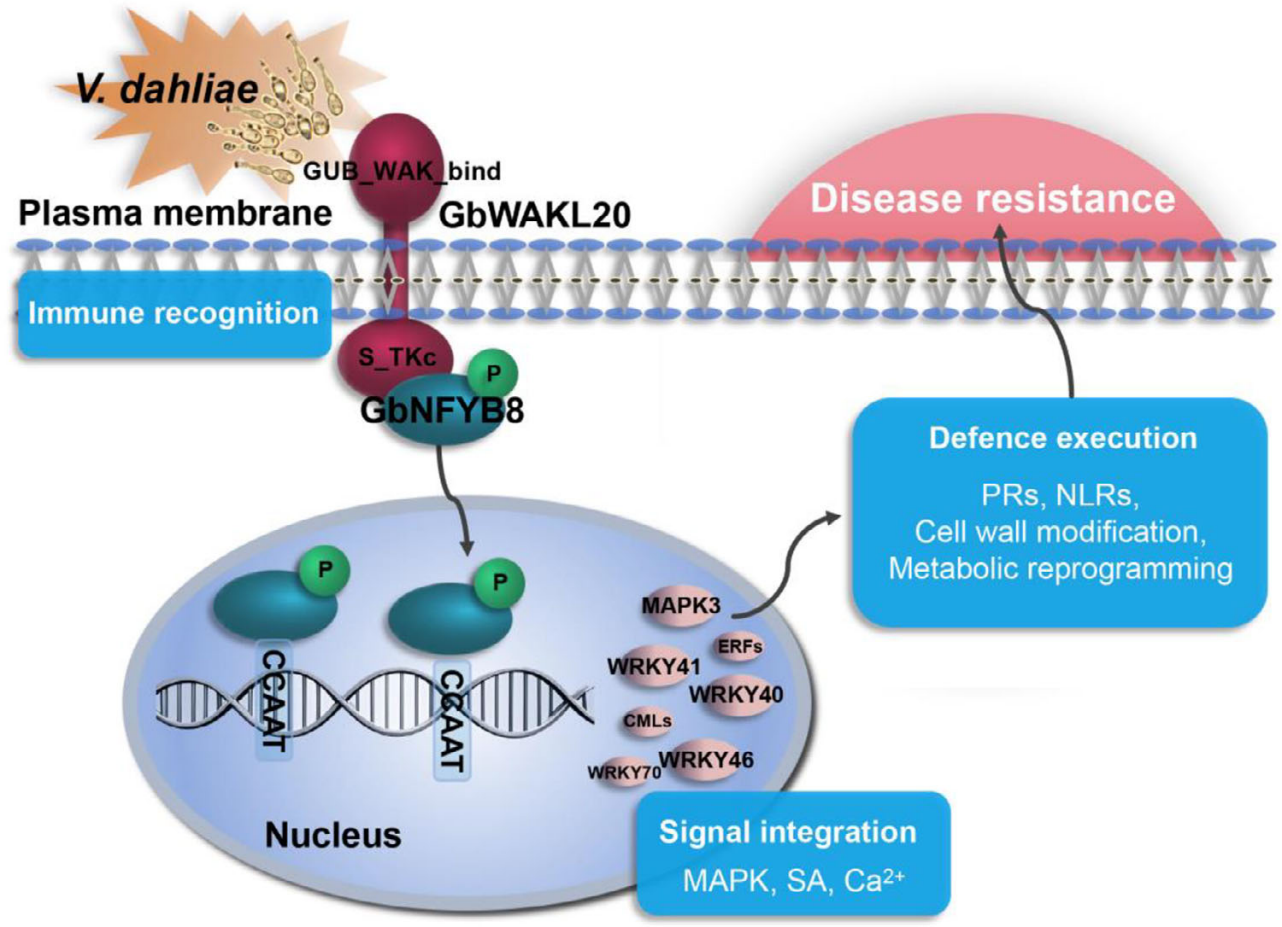
Model depicting the cooperative regulation of VW resistance by GbWAKL20 and GbNFYB8. GbWAKL20 senses *Vd* infestation at the cell membrane through the extracellular GUB_WAK_bind domain and transmits signals through its intracellular Serine‐Threonine kinase domain. GbWAKL20 interacts with and phosphorylates GbNFYB8, promoting its nuclear translocation and thereby transmitting defense signals from the plasma membrane along the endoplasmic reticulum‐mediated Golgi vesicle transport to the nucleus. The phosphorylated GbNFYB8 binds CCAAT elements, promoting the transcription of downstream immune‐related genes and activating the MAPK cascade and SA signaling pathway. This ultimately triggers defense responses, including the induction of defense‐related protein expression, cell wall modification, and metabolic reprogramming, collectively enhancing cotton resistance to VW.

Taken together, these findings demonstrate a novel defense mechanism in plants, with immune recognition beginning with GbWAKL20 at the plasma membrane upon sensing *Vd* infection, and further enhancing transcriptional activation of GbNFYB8 through phosphorylation. The signal is transmitted to the nucleus through the endoplasmic reticulum‐mediated Golgi vesicle transport, promoting the nuclear translocation of GbNFYB8. GbNFYB8 subsequently binds to the CCAAT element in the promoters of downstream immune‐related genes, triggering the activation of the MAPK cascade and hormone‐related signaling pathways. Multiple defense signals are subsequently integrated to modulate the release of disease resistance proteins, cell wall remodeling, and metabolic reprogramming. This defense execution ultimately enhances plant disease resistance (Figure 9).

## 3 Discussion

### 3.1 GbWAKL20 is a Key Pathogen Recognition Receptor in Cotton Immunity

WAK/WAKL proteins serve as critical mediators bridging the extracellular and intracellular compartments, enabling transmembrane signal transduction. Recent studies have highlighted their pivotal roles in plant–pathogen interactions and disease resistance [36]. For instance, Rlm9, a WAKL‐type resistance gene in *Brassica napus*, confers race‐specific resistance to blackleg by interacting with the effector AvrLm5‐9 [37]. GhWAK7A enhances cotton immunity against *Vd* and *F. oxysporum* by facilitating chitin‐induced GhCERK1‐GhLYK5 polymerization [29]. In rice, *OsWAKL21.2* is upregulated upon *Xanthomonas oryzae* infection, and its silencing compromises immunity, while its heterologous expression in Arabidopsis triggers defense activation [38]. The WAKL protein RFO1 in Arabidopsis is essential for both growth and early defense against *F. oxysporum* [39]. ZmWAKL‐ZmWIK‐ZmBLK1‐ZmRBOH4 module promotes maize defense responses against gray leaf spot by triggering a ROS burst [25]. *CsWAKL01* was shown to regulate phytohormone signaling to combat citrus bacterial canker [40]. In this study, we conducted a systematic analysis of the WAK/WAKL family in cotton and identified several WAKL genes strongly induced by *Vd* in roots, with *GbWAKL20* playing a key role in disease resistance (Figure S1 and Table S1). Silencing *GbWAKL20* in *G. barbadense* acc. Hai7124 significantly compromised disease resistance, whereas its heterologous overexpression in Arabidopsis enhanced disease tolerance (Figure 1c–g and Figure 4a–c).

WAKs/WAKLs, despite being called cell wall‐associated kinases, tend to be localized in the plasma membrane rather than the cell wall. The extracellular domains are used to sense external stress signals to the cell membrane, while the intracellular kinase domains are used to transmit signals. These receptors exhibit distinct organ‐specific expression patterns, reflecting their evolutionary adaptation to recognize diverse extracellular ligands [13]. In cotton, WAKs/WAKLs also showed diversity of expression, and most of them were expressed in roots, stems, leaves, and flower organs, while a few were expressed in ovules and fibers, which may be due to the former being more readily exposed to external stress environments (Figure S24). *GbWAKL20* was highly expressed in roots, stems, and floral organs, with significant up‐regulated expression following *Vd* infection, indicating it as a key pathogen recognition receptor in cotton (Figure 1a,b and Figure 2b). During host colonization, *Vd* employs a sophisticated infection strategy involving secretion of various toxins, cell wall‐degrading enzymes, and glycoproteins [31, 41]. This process affects the integrity of plant cell walls and releases many degradation products, such as pectin fragments, oligogalacturonides (OGs), and glycine‐rich proteins [22, 42, 43]. Simultaneously, plant‐derived hydrolases targeting fungal cell walls generate PAMPs including chitin oligosaccharides and β‐glucans [44]. These degradation products serve as critical defense signaling molecules that interact with the WAKs/WAKLs extracellular domain. Arabidopsis WAK1/2 have been well‐characterized as OG receptors [45], while cotton GhWAK7A appears to modulate immunity through an alternative mechanism, facilitating chitin‐induced GhLYK5‐GhCERK1 dimerization without direct OG responsiveness [29]. The sequence analysis revealed low conservation in the extracellular domain of WAKL20 orthologs, suggesting potential ligand specificity divergence across plant species (Figure S4). Future studies will focus on identifying the specific external signal sources recognized by GbWAKL20 and elucidating its role in cotton immune signaling networks.

### 3.2 GbWAKL20 Activates Defense Responses Through MAPK Cascade and SA Signaling Pathways

The intracellular kinase domain of WAKs/WAKLs is highly conserved and plays a crucial role in transmitting extracellular signals to activate systemic immune responses in plants. Although the molecular mechanism underlying WAKs/WAKLs‐mediated disease resistance remains largely unknown, accumulating evidence suggests their involvement in hormone signaling and ROS regulation. *AtWAK1* contributes to bacterial disease resistance, with its expression being induced by SA in an NPR1‐dependent manner [10]. The accumulation of SA and the activation of downstream NPR1 are necessary for the establishment of systemically acquired resistance, and ultimately improve the expression of the PR genes [46]. *CsWAKL08* in citrus can be induced by SA and JA, and confers resistance to bacterial canker by modulating ROS homeostasis and JA signaling [47]. *OsWAK91* in rice enhances pathogen resistance by reestablishing ROS homeostasis and upregulating PR genes [24]. In addition, WAKs/WAKLs also engage in immune signal transduction through MAPK cascade activation. For example, *AtWAK2* triggers innate immunity by activating *MPK3/6* [48]. Silencing *GhWAK7A* in cotton suppresses *GhMPK3/6* activation, ROS production and PR gene expression [29]. The comparative transcriptome analysis revealed that silencing *GbWAKL20* downregulates the MAPK cascade and hormone‐related genes (Figure 3a–c), whereas its overexpression in Arabidopsis enhances their expression upon *Vd* infection (Figure 4d–f). Further assays confirmed that *GbWAKL20* promotes SA accumulation and signaling in plants (Figure 3d–f and Figure 4g), and elevates MAPK phosphorylation post‐*Vd* challenge (Figure 4h). These results suggest that GbWAKL20‐mediated resistance involves MAPK cascade and SA signaling pathways, which may orchestrate downstream defense responses, including calcium homeostasis, defense protein accumulation, cell wall modification, and metabolic reprogramming (Figure S23).

### 3.3 GbNFYB8 is an Important Transcriptional Regulator in GbWAKL20‐Dependent Immunity

Nuclear factor Y (NF‐Y), ubiquitous in eukaryotes, functions as a transcription factor by specifically binding to the conserved CCAAT‐box promoter element, hence its alternative designation as the CCAAT‐binding factor (CBF) [49, 50]. NF‐Y is composed of three distinct subunits: NF‐YA, NF‐YB, and NF‐YC, all of which contain a highly conserved core domain essential for DNA binding [51]. While animals typically possess a single NFYB gene, plants exhibit significant gene family expansion, with 13, 18, 29, and 50 NFYB members in Arabidopsis, grape, tomato, and upland cotton, respectively [49, 52, 53, 54]. Due to the functional differentiation caused by the evolutionary process, the functions of NFYB are complex and remain poorly reported in plants. In this study, an NFYB transcription factor, GbNFYB8, was identified in cotton that interacts with GbWAKL20 (Figure 5). Like GbWAKL20, GbNFYB8 expression was strongly induced upon *Vd* infection (Figure 7a,b). Silencing *GbNFYB8* in *G. barbadense* compromised disease resistance, attenuating both MAPK cascade activation and SA signaling pathway‐mediated immune responses (Figure 7c–g and Figure S19). However, unlike GbWAKL20, GbNFYB8 exhibited broad constitutive expression across cotton diverse tissues and organs (Figure S25), implying pleiotropic roles in plant development and stress adaptation. Moreover, although the GbNFYB8 homolog GbNFYB8L1 also weakly interacts with GbWAKL20, it does not respond to *Vd* induction and exhibits higher expression during ovule and fiber development (Figures S12 and S13). This divergence in interaction strength and expression patterns implies distinct biological functions for the GbWAKL20/GbNFYB8 module compared to the GbWAKL20/GbNFYB8L1 interaction, warranting further mechanistic investigation.

The initiation of plant stress responses typically originates at the plasma membrane, while the implementation of defense mechanisms requires sophisticated nuclear transcriptional reprogramming. WAKLs are often thought to be membrane‐localized receptors that positively or negatively regulate immune responses during stress perception [55, 56]. Although GbWAKL20 also localizes to the plasma membrane, it transduces stress signals through Golgi‐derived vesicles along the membrane‐endoplasmic reticulum‐nuclear envelope axis, where it interacts intracellularly with GbNFYB8 to promote its accumulation within the nucleus and modulate nuclear gene transcription (Figure 5, Figure 6a–c, Figure S15, and Movies S1–S4). Transcriptomic analysis also confirmed that silencing *GbNFYB8* predominantly affects biological processes such as transcriptional regulation, molecular functions such as DNA binding, and nuclear‐related components, highlighting its central role in gene expression regulation (Figure 7f and Figure S20d). The MAPK cascade serves as a central hub of plant signal transduction systems, amplifying extracellular immune signals into intracellular responses through phosphorylating diverse substrates [57]. In Arabidopsis, MAPK3/6 plays a pivotal role in plant defense signaling. The cotton ortholog of *MAPK3*, *GhMPK9*, enhances resistance to VW by phosphorylating downstream components to activate PR gene expression [58]. WRKY transcription factors constitute another crucial component of plant immunity, functioning in defense against both biotrophic and necrotrophic pathogens [59]. For instance, Arabidopsis *AtWRKY33* is essential for resistance to *B. cinerea* and *Alternaria brassicicola*, while *AtWRKY46*, *AtWRKY53*, and *AtWRKY70* participate in SA‐mediated defense against *Pseudomonas syringae* [60, 61]. In tomato, *ShWRKY41* is induced by SA and ET, playing a positive role in defense activation against *O. neolycopersici* [62]. In chickpea, CaMPK9 phosphorylates CaWRKY40, thereby positively regulating plant resistance to *F. oxysporum* [63]. In cotton, *GhWRKY1‐like* and *GhWRKY41* enhance VW defense by modulating phenylpropanoid metabolism [64, 65]. *GhWRKY33* and *GhWRKY53* regulate cotton tolerance to VW through JA‐ and SA‐mediated signaling pathways [66, 67]. Despite low sequence conservation in the N‐ and C‐terminal regions among species, NFYB8 maintains a highly conserved CBFD_NFYB_HMF domain, suggesting evolutionary preservation of its DNA‐binding specificity (Figure S21). In this study, we demonstrate that transcript levels of MAPK3 and WRKY transcription factors were significantly reduced in *GbWAKL20*‐ and *GbNFYB8*‐silenced plants but increased in *GbWAKL20*‐overexpressing plants induced by *Vd*. Through combining element prediction, yeast one‐hybrid assays, dual‐luciferase reporter systems, and EMSA, we established that GbNFYB8 directly binds to the CCAAT elements in the promoters of both MAPK3 and WRKY genes, thereby promoting downstream defense response execution (Figure 8, Figure S22).

### 3.4 GbWAKL20 Phosphorylates GbNFYB8 to Promote Its Nuclear Translocation and Potentiate Transcriptional Activation of Defense Genes

Transcriptomic analysis revealed that neither *GbWAKL20* silencing in cotton nor its overexpression in Arabidopsis altered *NFYB8* transcript levels. Similarly, *GbNFYB8* silencing also had no effect on the transcriptional level of *GbWAKL20* (Figure S26). Therefore, we speculated that the GbWAKL20 may modulate GbNFYB8 activity post‐translationally. In vitro assays demonstrated that GbWAKL20 exhibits strong autophosphorylation activity and could phosphorylate GbNFYB8 (Figure 6d). The Ser‐76 residue within the conserved CBFD_NFYB_HMF domain of GbNFYB8 is critical for phosphorylation by GbWAKL20. Substitution of Ser‐76 with Ala prevents this phosphorylation event and consequently blocks the nuclear translocation of GbNFYB8 (Figure 6e and Figures S16,S17). Moreover, the intracellular kinase domain of GbWAKL20 enhances the binding of GbNFYB8 to CCAAT‐box elements, thereby upregulating downstream gene expression (Figure 8c–f). These results indicate that GbWAKL20 promotes GbNFYB8 nuclear translocation and activates its transcriptional activity via phosphorylation. Phosphorylation‐induced nuclear translocation of transcription factors represents a critical mechanism for activating plant immune responses. Interestingly, structural modeling revealed that the extracellular GUB_WAK_bind structural domain of GbWAKL20 (amino acids 166–168) and the intracellular kinase structural domain (amino acid 504) can form a connecting bridge, which makes GbWAKL20 resemble a hairpin in its 3D structure just enough to sandwich the CBFD_NFYB_HMF structural domain of GbNFYB8 in the middle (Figure S27a–c). The results predicted by AlphaFold3 also suggest a potential interaction between the intracellular kinase domain of GbWAKL20 and the CBFD_NFYB_HMF domain of GbNFYB8 (Figure S27d,e). We propose that this bridge may be important for the formation and phosphorylation of the GbWAKL20‐GbNFYB8 module, though the mechanistic details require further validation.

In conclusion, our findings reveal a novel pathway of the GbWAKL20‐GbNFYB8 module conferring VW resistance. GbWAKL20 interacts with and phosphorylates GbNFYB8, promoting its accumulation within the nucleus, which enhances the binding of GbNFYB8 to the promoters of downstream MAPK3 and WRKY transcription factors, thereby activating both the MAPK cascade and SA signaling pathway to execute defense responses.

## 4 Experimental Section

### 4.1 Plant Materials and Treatments

The expression of *GbWAKL20* and *GbNFYB8* was analyzed in *G. barbadense* acc. Hai7124 and *G. hirsutum* acc. TM‐1 following different stress treatments, respectively. The gene prefixed as *Gb* or *Gh* was following the origin from *G. barbadense* (*Gb*) acc. Hai7124 and *G. hirsutum* (*Gh*) acc. TM‐1, respectively. The cotton seedlings were planted in the same controlled greenhouse under the following conditions: 16 h light (28°C)/8 h dark (25°C) cycle for 2 weeks. The Arabidopsis plants (wild‐type Col‐0 and *GbWAKL20*‐overexpressing transgenic lines) were grown in a controlled environmental chamber maintained at 12 h light (23°C)/ 12 h dark (21°C) conditions for 4 weeks. All necessary permits for collecting Hai7124 and TM‐1 were obtained from Nanjing Agricultural University, Jiangsu, China.

The *Vd* V991, a highly aggressive and defoliating strain, which causes wilting and defoliation in cotton, was cultured on potato dextrose agar (PDA) medium at 25°C for 5 days, and then transferred to Czapek's liquid medium in a shaker (25°C, 180 rpm) for further incubation for 5–7 days [68]. The concentration of conidia was adjusted with sterile water to 1 × 10^7^ conidia mL^−1^ and used to inoculate plant seedlings.

To detect the *GbWAKL20* and *GbNFYB8* transcript levels in Hai7124 and TM‐1 after V991 treatment, the roots of cotton seedlings were sampled at 0, 6, 12, 24, 48, and 72 hours after inoculation using the dip‐inoculation method, with sterile water‐treated cotton seedlings as controls [69, 70].

The seedlings of Hai7124 were subjected to treatments with four different solutions of defense‐related signaling molecules, containing 100 mm salicylic acid (SA), 100 µm jasmonic acid (JA), 1 mm ethylene (ET), and 10 mm hydrogen peroxide (H_2_O_2_), respectively. Sterile water served as a control for the solvent, and leaf samples were collected at intervals of 0, 1, 2, 4, 6, 8, 10, and 12 hours post‐treatment. Three biological repeats for each treatment were collected, rapidly frozen in liquid nitrogen, and subsequently stored at −80°C before RNA extraction.

### 4.2 RNA Isolation and Gene Expression Pattern Analysis

For the analysis of gene expression, the total RNA was extracted from plant tissues utilizing the FastPure Universal Plant Total RNA Isolation Kit (RC411, Vazyme Biotech Co., Ltd., Nanjing, China) for plant tissues. The extracted RNA was subsequently reverse transcribed into cDNA employing the HiScript IV All‐in‐One Ultra RT SuperMix for qPCR Kit (R433, Vazyme Biotech Co., Ltd., Nanjing, China) for RT‐qPCR. Gene‐specific primers for RT‐qPCR were designed using Beacon Designer 7.0 software. The cotton *histone 3* (AF024716) and Arabidopsis *AtUbq5* (AT3G62250) were used as reference genes, respectively. All experimental reactions were conducted in triplicate, with primer information detailed in Table S12. The RT‐qPCR assays were executed on a Bio‐Rad CFX‐96 PCR system using PerfectStart Green qPCR SuperMix (AQ601, TransGen Biotech Co., Ltd., Beijing, China). The high‐throughput RNA sequencing data from *G. barbadense* acc. Hai7124 and *G. hirsutum* acc. TM‐1 were utilized to investigate the expression patterns of *GbWAKL20* and *GbNFYB8* homologs in various tissues [71].

### 4.3 Construction of VIGS Vectors and Agrobacterium‐Mediated VIGS Experiments

The 300–500 bp *GbWAKLs* and *GbNFYB8* gene‐specific fragments were inserted into the TRV2 vector. The empty vectors TRV: 00 and TRV: *GbCLA1* (cloroplastos alterados 1) served as negative and positive controls, respectively. These plasmids were subsequently transformed into the *Agrobacterium tumefaciens* (*A. tumefaciens*) strain GV3101 and injected into the cotyledons of cotton seedlings. Following a two‐week period post‐injection, RNA was extracted from the roots to detect the transcript levels of *GbWAKLs* and *GbNFYB8* using RT‐qPCR analysis. At least 30 plants from each VIGS treatment were selected for the V991 infection assay, and the ratio of diseased to healthy leaves was investigated. To investigate invasive growth in cotton, the stems infected with *Vd* were cut and observed using a stereoscope (LEICA DVM6, Germany). Detailed primer information is provided in Table S12.

### 4.4 Fungal Biomass Assays

To assess the relative biomass quantification of *Vd* infiltrating into the interior of plant tissues, samples from V991‐treated plant tissues were collected for DNA extraction. The ribosomal DNA's Internal Transcribed Spacer (ITS) region was targeted using the *Vd*‐specific ITS1‐F primer and STVe1‐R reverse primer. Additionally, primers for *histone 3* in cotton and *AtUbq5* in Arabidopsis served as endogenous plant controls. The qPCR was conducted on the extracted genomic DNA [72]. Detailed primer information is provided in Table S12.

### 4.5 Fungal Recovery Experiments

To assess the *Vd* infection rate, the approximately 0.1 cm stem segment above the cotton cotyledons was individually sampled and surface sterilized with 70% ethanol (v/v), followed by at least four rinses with sterile water. The stem segments were then transferred onto the PDA plates supplemented with chloramphenicol (34 mg L^−1^) and incubated for 3 days before photographing.

### 4.6 Subcellular Localization

To investigate the localization of GbWAKL20 and GbNFYB8 within the *N. benthamiana* leaf cells, the ORF of *GbWAKL20* and *GbNFYB8* were fused to GFP within the pBinGFP4 expression vector. These vectors were then transiently expressed in the leaf cells of *N. benthamiana* through the *A. tumefaciens* infiltration technique, and co‐expressed in leaves with Golgi bodies, cell membrane, endoplasmic reticulum, or nucleus marker genes [73, 74, 75, 76]. Three days post‐infiltration, the infiltrated leaves were collected for analysis, and fluorescence signals from the GFP and RFP were observed using a confocal microscope (LSM780, Zeiss, Jena, Germany) at 488 and 561 nm, respectively.

### 4.7 Transcriptome Sequencing

The cotton and Arabidopsis root samples were collected following treatment with either V991 or sterile water, respectively. Total RNAs were subsequently extracted and utilized for the construction of the RNA‐seq libraries. Sequencing was conducted on the Illumina NovaSeq6000 platform. Following the removal of adapters using Cutadapt, the reads were mapped to *G. barbadense* acc. Hai7124 (ZJU_v1.1) and Arabidopsis (TAIR10) genome using Hisat2 with default parameters [77]. The quantity of aligned reads was determined using HTSeq software and then imported into R statistical software for DEGs analysis with DESeq2 (*q *< 0.05, log_2_FC > 1) [78]. The gene expression values were normalized through FPKM analysis. Hierarchical clustering, GO, and KEGG analysis were performed using the Omicshare website (https://www.omicshare.com/).

### 4.8 Determination of the Total SA Content

The SA was extracted and quantified following the method described by Xiao et al. with slight modifications [79]. In brief, the roots from cotton and Arabidopsis were collected and subsequently ground in liquid nitrogen. Approximately 0.2 g of the resulting plant powder was placed in 1.5 mL tubes, followed by the addition of 750 µL of 80% (v/v) methanol. The samples underwent extraction overnight in a shaker at 4°C, shielded from light, and were then centrifuged at 12 000 rpm for 15 min. The supernatant was collected, and an additional 250 µL of extraction buffer was introduced to facilitate further extraction for 2 hours. Following a second centrifugation, the two supernatants were combined. The resultant supernatant was then completely evaporated using a freeze‐dryer. The remaining residue was reconstituted in 0.5 mL of 20% (v/v) methanol and filtered through a 0.22 µm membrane. SA content was determined using liquid chromatography‐tandem mass spectrometry (LC‐MS/MS) (SCIEX Triple Quad 6500+ system), and quantification was performed based on a standard SA curve generated from commercially obtained SA from Sigma (S7401, Sigma‐Aldrich, Saint Louis, MO, USA).

### 4.9 Total Protein and Nuclear Proteins Extraction

The total protein and nuclear proteins were extracted using the Plant Protein Extraction Kit (BC3720, Solarbio Life Science, Beijing, China) and Plant Nuclear Proteins Extraction Kit (BB31541, BestBio, Shanghai, China) with 1 mm proteinase inhibitor and phosphatase inhibitor cocktail (CW2383S, CWBIO Co., Ltd., Beijing, China). All experiments were completed following the supplier's operation manual. Coomassie brilliant blue (CBB) staining was used to normalize protein amounts.

### 4.10 MAP Kinase Activity Analysis

At several time points, 4‐week‐old Arabidopsis seedlings were treated with *Vd* and sampled to extract total protein. The total proteins were subjected to 10% SDS‐PAGE. After electrophoresis, the proteins were transferred onto nitrocellulose membranes. They were then incubated with anti‐phospho‐p44/42 MAPK (Erk1/2) (Thr202/Tyr204) (9101S, Cell Signaling Technology, Danvers, MA, USA) primary antibodies and then the corresponding secondary antibodies. Then, the proteins were visualized using a SuperPico ECL Chemiluminescence Kit (E422, Vazyme Biotech Co., Ltd., Nanjing, China).

### 4.11 Identification of VW Resistance in GbWAKL20 Transgenic Arabidopsis Plants

The full‐length sequences of the *GbWAKL20* were inserted into the pBI121 vector with the 35S promoter. Subsequently, the overexpression vectors were transferred into the GV3101 strain for the transformation of Arabidopsis using the floral dip method. Pure lines were subsequently screened within a controlled growth chamber environment. DNA and RNA were extracted from the roots of the transgenic lines to identify positive transformants. For the inoculation of Arabidopsis, the roots of 4‐week‐old seedlings were rinsed in water and then immersed in a suspension of *Vd* conidia (1 × 10^7^ conidia mL^−1^) for a duration of 90 s, after which the plants were replanted in fresh soil. The severity of disease symptoms was assessed using an index ranging from 0 (healthy plant) to 4 (dead plant) for the calculation of the disease index (DI). DI was calculated according to the following formula: DI = [(∑disease grades × number of infected plants)/(total number of plants assessed × 4)] × 100% [80].

### 4.12 Yeast Two‐Hybrid (Y2H) Screen and Validation

To screen the GbWAKL20 interacting proteins, the root samples from the Hai7124 treated with V991 at different times were utilized to construct a yeast expression cDNA library. The Y2H assay was conducted in accordance with the guidelines provided in the Matchmaker Y2H system manual (Clontech, San Francisco, CA, USA). The CDS region of *GbWAKL20* without signal peptide was cloned into the pGBKT7 vector to serve as the bait construct. Subsequently, the cotton cDNA library and the linearized AD‐rec vector were co‐transformed into yeast cells using the Y2H screening methodology. Positive clones were screened on SD/‐Leu/‐Trp/‐His TDO medium. The validated colonies were further selected on quadruple‐dropout (SD/‐Leu/‐Trp/‐His/‐Ade QDO) medium supplemented with 40 mg L^−1^ X‐α‐Gal to assess galactosidase activity.

### 4.13 Firefly Luciferase Complementation Imaging (LCI) Assay

For the LCI assay, full‐length cDNAs of *GbWAKL20* and *GbNFYB8* were ligated with the pCambia1300‐nLuc and pCambia1300‐cLuc vectors to generate NLuc‐GbWAKL20 and CLuc‐GbNFYB8. The Cluc‐GbNFYB8L1‐3, Cluc‐GbNFYB3L1‐4, and NLuc‐GbWAKL20^ECD^ vector constructed in the laboratory was used as a negative control. The *Agrobacterium* strains containing the recombinant plasmids were combined in a 1:1 ratio and co‐transferred into the lower epidermal cells of *N. benthamiana* leaves, then the plants were incubated in the dark for 48 hours. The luciferase was subsequently measured using 1 mm D‐luciferin sodium salt (Promega, Madison, WI, USA) and detected with a low‐light cooled charge‐coupled device camera (Tanon 5200 Multi, Shanghai, China).

### 4.14 BiFC Assay

For the BiFC assay, the full‐length cDNAs of *GbWAKL20* and *GbNFYB8* were fused with the SPYNE(R)173 and SPYCE(M) vectors in frame with YFP‐N and YFP‐C to generate YN‐GbWAKL20 and YC‐GbNFYB8, respectively. These vectors were subsequently transformed into *Agrobacterium* and co‐expressed in the lower epidermal cells of *N. benthamiana* leaves. After 72 hours post‐infiltration, the *N. benthamiana* leaves were placed in nucleus staining solution (1 mm DAPI) for 15 min before observation under a microscope. YFP fluorescence was imaged using a confocal microscope (LSM780, Zeiss) at an excitation wavelength of 514 nm.

### 4.15 Co‐Immunoprecipitation Assay

For the co‐immunoprecipitation (Co‐IP) assay, the full‐length cDNAs of *GbWAKL20* and *GbNFYB8* (including *GbNFYB8* homologs and *GbNFYB8* with mutated phosphorylation sites) were constructed on pCambia1390‐3×Flag and pBinGFP4‐1×GFP vectors, respectively. These vectors were subsequently transformed into *A. tumefaciens*, and co‐expressed in the *N. benthamiana* leaves. Three days post‐infiltration, the treated leaves were collected for total protein extraction. An equal volume of the crude extract was then incubated with Flag beads (SM009001, Smart‐Lifesciences, Changzhou, China) for a duration of 4 hours. The immunoprecipitated proteins were rinsed with ice‐cold PBS buffer and analyzed by immunoblotting with Flag, GFP, and pSer/pThr antibodies (M185‐7 and M048‐3, MBL Co., Ltd, Nagoya, Japan; 31553ES60, Yeasen Biotechnology Co., Ltd., Shanghai, China).

### 4.16 In Vitro Glutathione S‐Transferase (GST) Pull‐Down Assays

The interaction between GbWAKL20 and GbNFYB8 was confirmed through an in vitro pull‐down assay. The GbWAKL20^ICD^ (without extracellular GUB_WAK_bind domain and SP) was inserted into the pET‐30a vector to construct a His‐fusion plasmid, and GbNFYB8 was inserted into the pGEX4T‐2 vector to generate a GST‐fusion plasmid, respectively. The *E. coli* strain BL21 (DE3) was transformed with each fusion plasmid, and the expression of the recombinant proteins was induced overnight at 20°C using 0.5 mm IPTG. The proteins were subsequently purified using Ni‐Agarose Resin and Glutathione‐Sepharose Resin (Genentech, San Francisco, CA, USA), respectively. Following the ProFound Pull‐Down GST Protein–Protein Interaction Kit (Pierce, Thermo Fisher Scientific, Waltham, MA, USA), the purified GST proteins were incubated with 200 µL of Glutathione Agarose Beads for 2 hours at 4°C, then washed five times and incubated overnight with an equivalent amount of purified His‐GbWAKL20^ICD^ purified protein. The beads were washed more than eight times again, and the His‐GbWAKL20^ICD^ protein was detected by immunoblotting using anti‐His antibody (A02003 and A00097, Genscript, Nanjing, China) [81].

### 4.17 In Vitro Phosphorylation Assay

The in vitro phosphorylation assay was performed according to standard methods [58]. Protein inputs were assessed using CBB staining. For the western blot analysis, the purified His‐GbWAKL20^ICD^ recombinant protein and the purified GST‐GbNFYB8 recombinant protein were incubated in a kinase reaction buffer composed of 25 mm Tris‐HCl (pH = 7.5), 10 mm MnCl_2_, 1 mm DTT, and 200 µm ATP at 30°C for 30 min. The reactions were terminated by the addition of 5 × SDS loading buffer, followed by boiling for 5 min. The phosphorylated proteins were subsequently visualized by 10% SDS‐PAGE gel followed by immunoblotting with biotin‐pendant Zn^2+^‐Phostag according to the manufacturer's guidelines. Western blot analysis of phosphorylated proteins‐chemiluminescent detection using biotinylated Phos‐tag (BTL‐111, Nard, Amagasaki, Japan).

### 4.18 Yeast One‐Hybrid (Y1H) Assay

The Matchmaker Gold Yeast One‐Hybrid System (Clontech) was employed to examine the potential binding affinity of the GbNFYB8 protein to the CCAAT element (mutated CCCCT element as negative control). The CCAAT and CCCCT elements were cloned into the yeast expression vector pHIS2 to create the respective pHIS2‐bait plasmids. The *GbNFYB8* gene was inserted into the pGADT7 vector to produce the pGADT7‐GbNFYB8 construct. The polyethylene glycol‐mediated transformation method was utilized to co‐transform the *Saccharomyces cerevisiae* (*S. cerevisiae*) Y1H Gold strain with the pHIS2‐ and pGADT7‐based vectors. The assessment of DNA‐protein interactions was conducted by evaluating the growth status of yeast cells cultured on selective SD/‐Trp/‐Leu/‐His selective medium supplemented 40 mm 3‐AT (the minimal inhibitory concentration) for three days at 30°C.

### 4.19 Electrophoretic Mobility Shift Assay (EMSA)

The 55‐bp promoter fragments of *GbMAPK3*, *GbWRKY40*, *GbWRKY41*, or *GbWRKY46* containing the CCAAT elements were labeled with biotin and employed as the detection probes. The unlabeled competitor DNA probe was synthesized. The signals from the labeled probes were detected utilizing the Light Shift Chemiluminescent EMSA kit (RE231894, Thermo Fisher Scientific, Waltham, MA, USA). Specifically, the purified GST‐GbNFYB8 protein was incubated with the DNA probe for 30 min at room temperature in EMSA/Gel‐Shift binding buffer (GS005, Beyotime Biotechnology, Shanghai, China). The DNA–protein complexes were then separated using a 6.5% polyacrylamide gel, subsequently transferred to a nylon membrane, and detected following the manufacturer's guidelines. Primers used are listed in Table S12.

### 4.20 Promoter‐Luciferase (LUC) Activity Assay

For the LUC assay, the CCAAT‐elements or *GbMAPK3*, *GbWRKY40*, *GbWRKY41*, and *GbWRKY46* promoter fragments were fused upstream of the mini35S::*LUC* construct to create the reporter vector pGreen II 0800‐LUC. The effector constructs, pBI121‐35S::*GbNFYB8*, pBI121‐35S::*GbWAKL20*, and the empty pBI121 vector served as the effector and negative control, respectively. The effector and reporter plasmids were transformed into *A. tumefaciens* GV3101 strain. *A. tumefaciens*‐mediated transformation of *N. benthamiana* leaf cells was conducted using a mixture of *Agrobacterium* cells containing the effector and the reporter plasmids in a 5:1 ratio. Following infiltration, the *N. benthamiana* were incubated in darkness for 12 hours before being transferred to standard growth conditions at 25°C for an additional 48 hours. The LUC activities were assessed using a histochemical method and captured with a low‐light cooled charge‐coupled device camera (Tanon 5200 Multi, Shanghai, China). The activities of LUC and Renilla luciferase (REN) were quantified utilizing the Dual‐Luciferase Reporter Assay System (Promega, Madison, USA) in conjunction with an Infinite200 Proreader (Tecan, Switzerland). The transient expression assay was conducted with at least three biological replicates, and the promoter activity was represented as the ratio of LUC to REN.

### 4.21 Statistical Analysis

At least three independent biological replicates for each experiment were used in this study. The figure legends provide detailed information on sample size (*n*) for each statistical analysis. The standard deviation (SD) of the mean can be visualized in the figures as error bars. SPSS software (SPSS 27.0.0; SPSS, Chicago, IL, USA) was used to conduct statistical analysis. To determine significant differences between the two groups, the two‐tailed paired Student's *t*‐test was used. Asterisks (*) and double asterisks (**) indicate statistical significance at *P *< 0.05 and *P *< 0.01.

## Author Contributions

W.G. and G.W. designed the research. G.W., Q.S., Z.C., Z.Y., L.W., and Z.G. performed research. G.W., W.L., and W.G. analyzed data. G.W. and W.G. wrote the paper. W.G. revised the paper.

## Conflicts of Interest

The authors declare no conflict of interest.

## Supporting information




**Supporting File 1**: advs73734‐sup‐0001‐SuppMat.pdf.

**Supporting File 2**: advs73734‐sup‐0002‐TableS1‐S12.xlsx.

**Supporting File 3**: advs73734‐sup‐0003‐MovieS1.mp4.

**Supporting File 4**: advs73734‐sup‐0004‐MovieS2.mp4.

**Supporting File 5**: advs73734‐sup‐0005‐MovieS3.mp4.

**Supporting File 6**: advs73734‐sup‐0006‐MovieS4.mp4.

## Data Availability

All data are available in the main text or the supplementary materials. The RNA‐seq data from this study have been deposited in the National Center for Biotechnology Information Sequence Read Archive under accession number PRJNA1260225.
