# Supplementary material for: GbWAKL20 Phosphorylates GbNFYB8 to Modulate Verticillium Wilt Resistance in Cotton
Source: Adv Sci (Weinh). 2026 Jan 11;13(16):e15724. doi: 10.1002/advs.202515724 (PMC13042932; doi:10.1002/advs.202515724)
Supplement: Supplementary file 1 — Supporting File 1: advs73734‐sup‐0001‐SuppMat.pdf. [file ADVS-13-e15724-s002.pdf]

Supplemental information

GbWAKL20 Phosphorylates GbNFYB8 to Modulate Verticillium wilt Resistance in Cotton

Guilin Wang, Qingxin Si, Zhiguo Chen, Zhe Yu, Zhan Guo, Lu Wang, Weixi Li, Wangzhen Guo *

Corresponding author:

Wangzhen Guo (moelab@njau.edu.cn);

This PDF file includes:

Supplemental Figures S1 to S27

Supplemental Figures

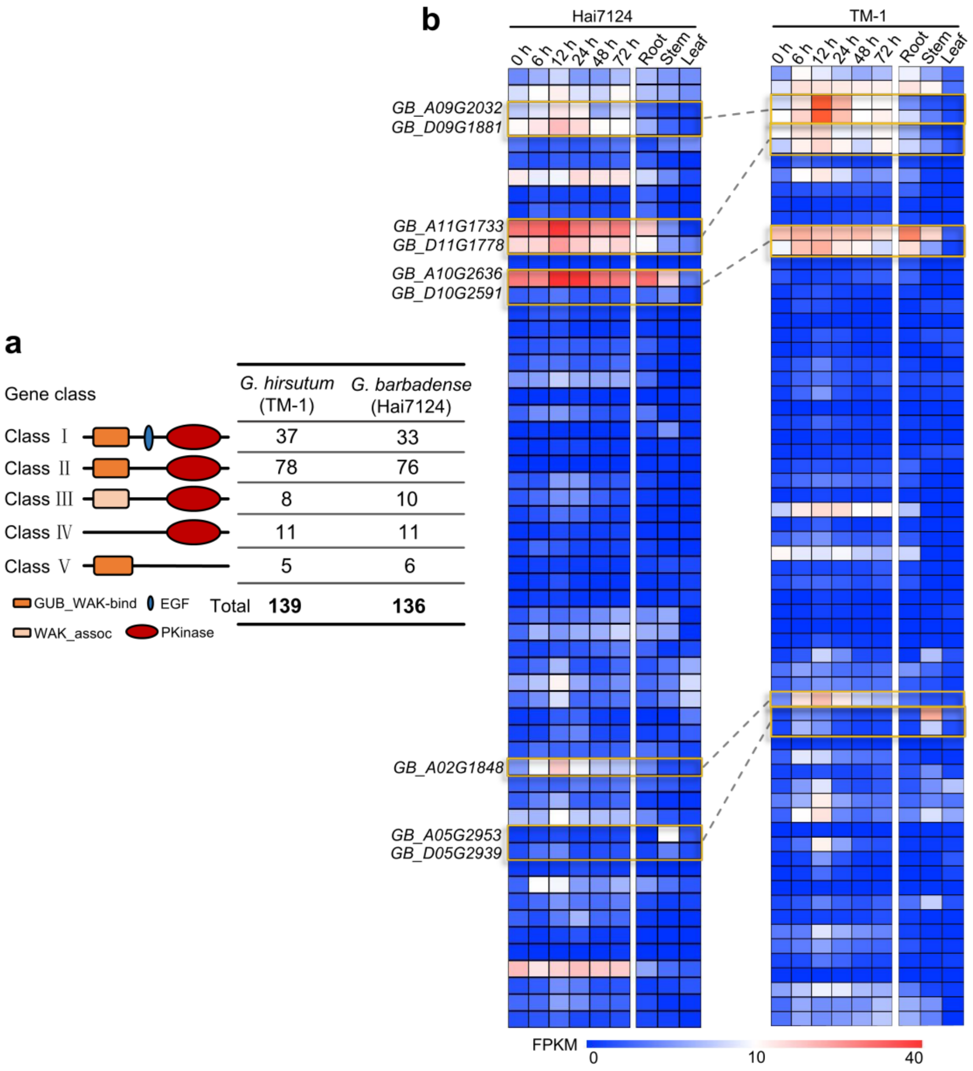

Figure S1 Classification and expression analysis of WAKs/WAKLs family genes in cotton. a) The WAKs/WAKLs genes in *G. barbadense* acc. Hai7124 and *G. hirsutum* acc. TM-1 were identified based on the GUB_WAK_bind domain, the C-terminal WAK_assoc residues, the EGF domain, and the intracellular Serine/Threonine kinase domain. The genes were categorized into five groups according to these domains. **b)** Expression patterns of WAKs/WAKLs in roots, stems, and leaves in cotton seedlings, and their induced expression profiles in response to *Vd* infection. Expression data for roots, stems, and leaves were obtained from http://www.ncbi.nlm.nih.gov/bioproject/503814. Roots of Hai7124 and TM-1 were sampled for transcriptome analysis at 0, 6, 12, 24, 48 and 72 hours after *Vd* inoculation. Each treatment included three biological replicates (n=3). A heat map was generated with Multi Experiment Viewer V.4.9 (http://en.bio-soft.net/chip/MeV.html). The expression data were converted to

Fragments Per Kilobase of exon model per Million mapped fragments (FPKM) to calculate the expression levels of the genes. Colored squares indicated expression levels from 0 (blue) to 40 (red).

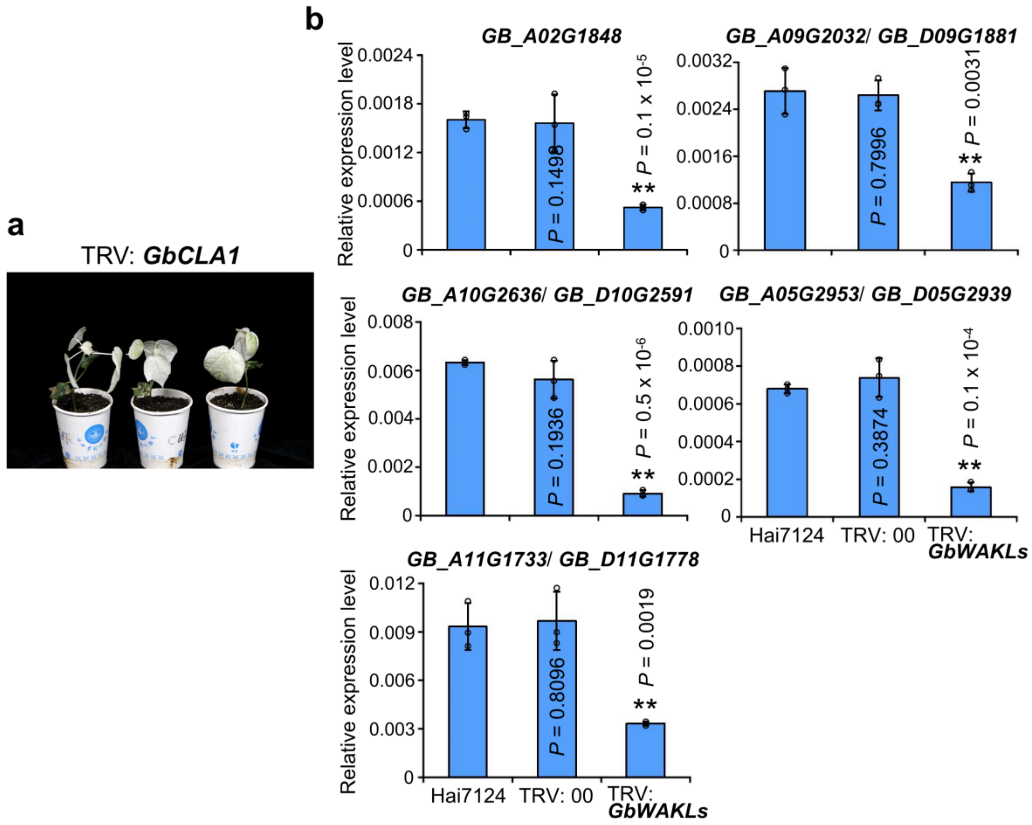

Figure S2 Verification of VIGS silencing system. a) Silencing of the endogenous cloroplastos alterados gene (*GbCLA1*) in cotton through tobacco rattle virus (TRV)-mediated virus-induced gene silencing (VIGS). Seven-day-old cotton seedlings (Hai7124) with two fully expanded cotyledons were infiltrated with TRV: *GbCLA1*, and the leaf bleaching phenotype was observed two weeks later. **b)** The efficiency of silencing *GbWAKL* genes each was verified by RT-qPCR in the roots of VIGS plants. Error bars represent the standard deviation of three biological replicates (n=3). Statistical analyses were performed using Student's *t*-test (***P*<0.01).

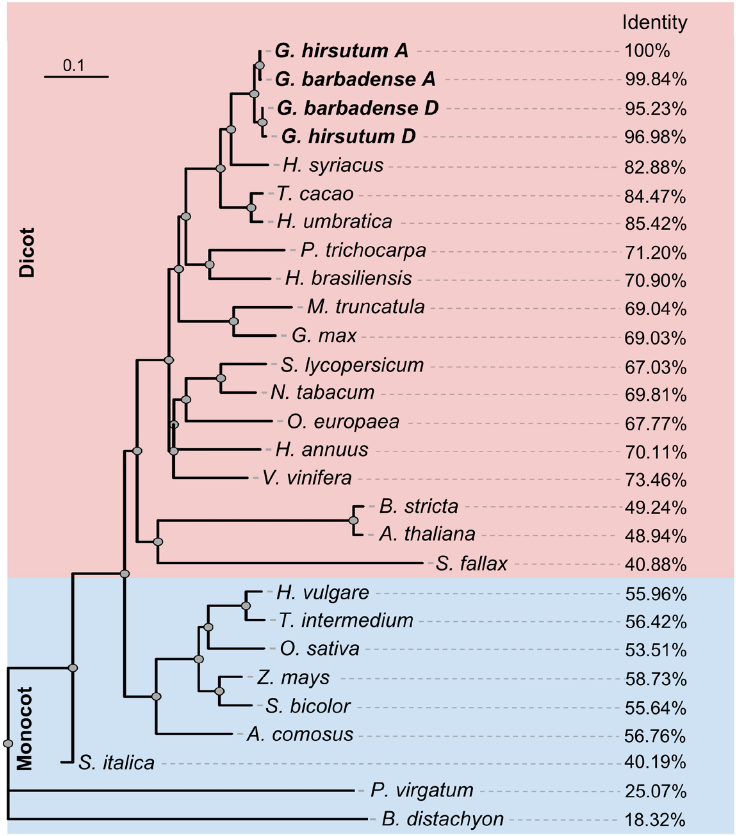

Figure S3 Phylogenetic analysis of WAKL20 homologs in different plant species. Phylogenetic tree of WAKL20 **homologs** from *Gossypium hirsutum*, *Gossypium barbadense*, *Hibiscus syriacus*, *Theobroma cacao*, *Herrania umbratica*, *Populus trichocarpa*, *Hevea brasiliensis*, *Medicago truncatula*, *Glycine max*, *Solanum lycopersicum*, *Nicotiana tabacum*, *Olea europaea*, *Helianthus annuus*, *Vitis vinifera*, *Boechera stricta*, *Arabidopsis thaliana*, *Sparganium fallax*, *Hordeum vulgare*, *Thinopyrum intermedium*, *Oryza sativa*, *Zea mays*, *Sorghum bicolor*, *Ananas comosus*, *Setaria italica*, *Panicum virgatum*, and *Brachypodium distachyon*. The neighbour-joining tree was constructed using the MEGA5.1 program (http://www.megasoftware.net/). The identity value was relative to GbWAKL20 and was calculated using DNAMAN software (http://www.lynnon.com/).

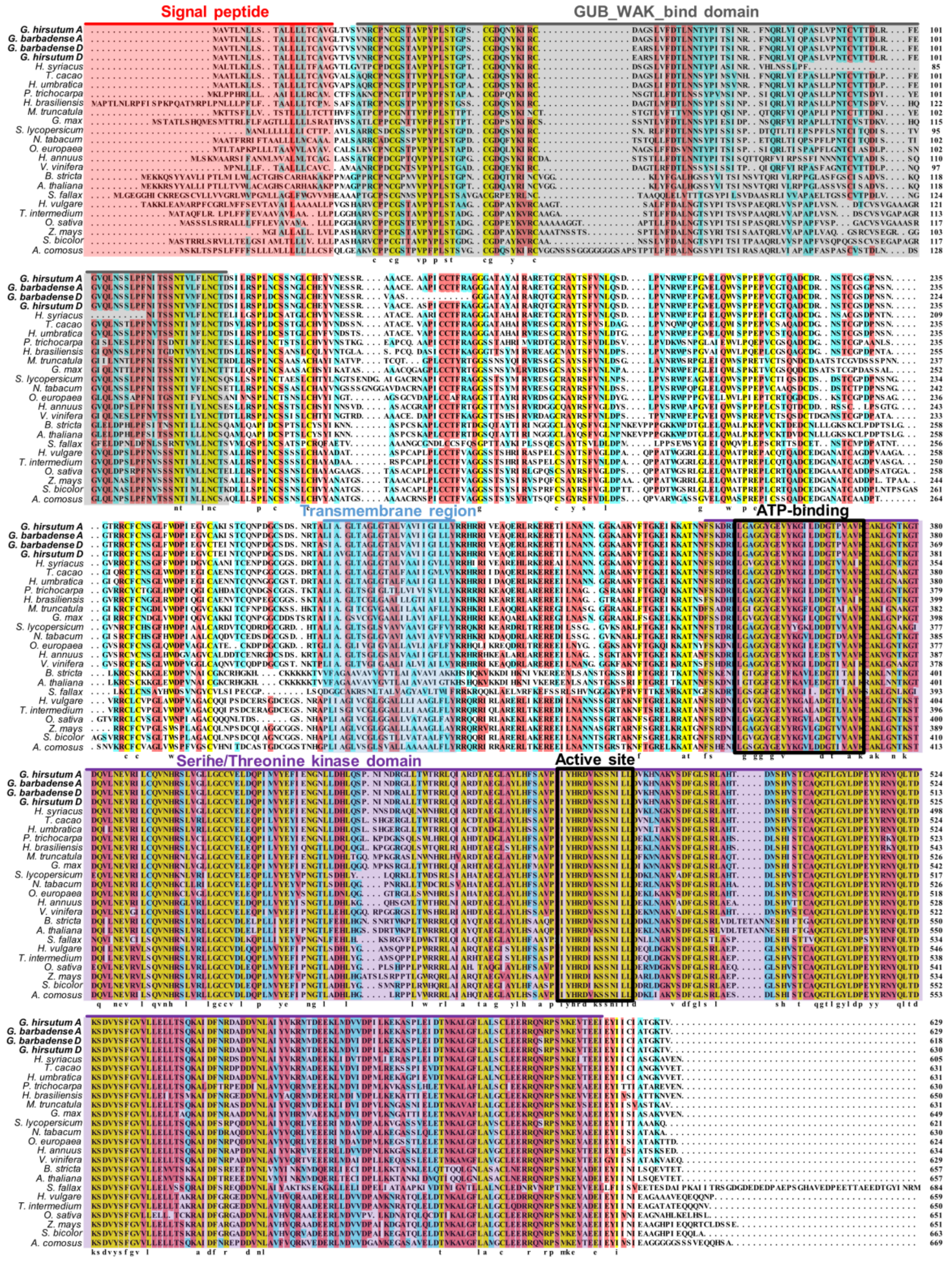

Figure S4 Sequence alignment of WAKL20 homologs from different plant species. Amino acid sequence alignment of WAKL20 homologs in *Gossypium hirsutum*, *Gossypium barbadense*, *Hibiscus syriacus*, *Theobroma cacao*, *Herrania umbratica*, *Populus trichocarpa*, *Hevea brasiliensis*, *Medicago truncatula*, *Glycine max*, *Solanum lycopersicum*, *Nicotiana tabacum*, *Olea europaea*, *Helianthus annuus*, *Vitis vinifera*, *Boechera stricta*, *Arabidopsis thaliana*, *Sparganium fallax*, *Hordeum vulgare*, *Thinopyrum intermedium*, *Oryza sativa*, *Zea mays*, *Sorghum bicolor*, *Ananas comosus*, *Setaria italica*, *Panicum virgatum*, and *Brachypodium distachyon* were

performed using software DNAMAN (http://www.lynnon.com/). Identical amino acid residues are highlighted in yellow. The Signal peptide, GUB_WAK_bind domain, Transmembrane region, and the Serine/Threonine kinase domain are labeled with red, gray, blue and purple colors. The black box shows ATP-binding motif and Active site in kinase domain.

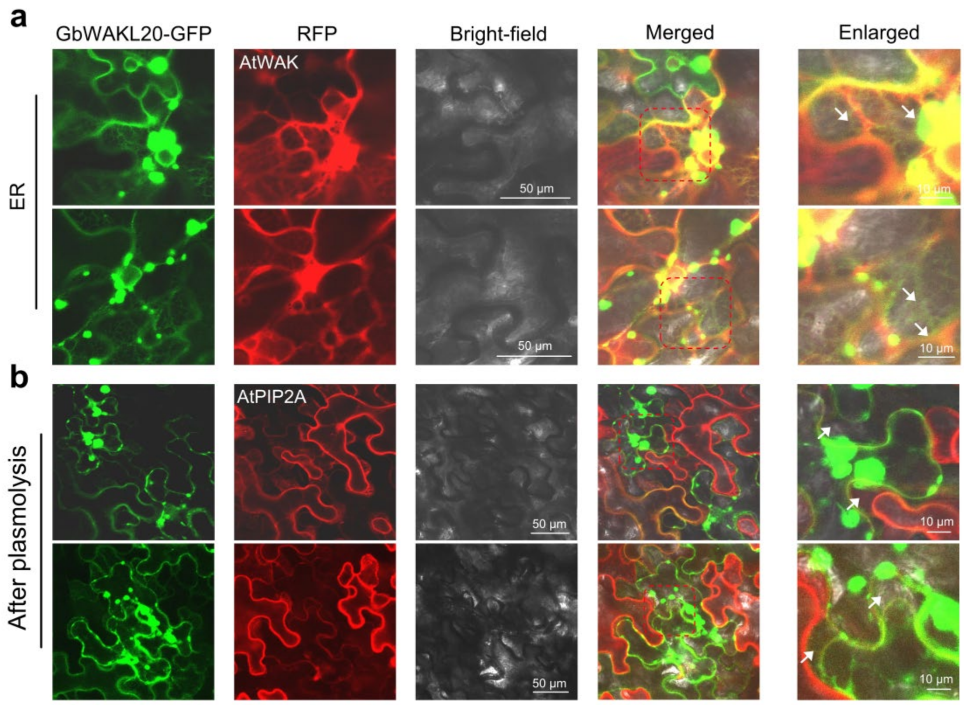

Figure S5 Subcellular localization of GbWAKL20 in *N. benthamiana* epidermal cells. a) The GbWAKL20-GFP fusion co-localizes with an endoplasmic reticulum (ER) marker (AtWAK, wall-associated kinase 2). White arrows indicate overlapping regions of the endoplasmic reticulum. **b)** After *N. benthamiana* leaves were treated with 0.8 M mannitol solution, which induced plasmolysis, GbWAKL20-GFP remained localized inside the plasma membrane. White arrows show the apoplast space. Images were captured by a confocal microscopy (LSM 780; Zeiss). Scale bars: 50 µm and 10 µm.

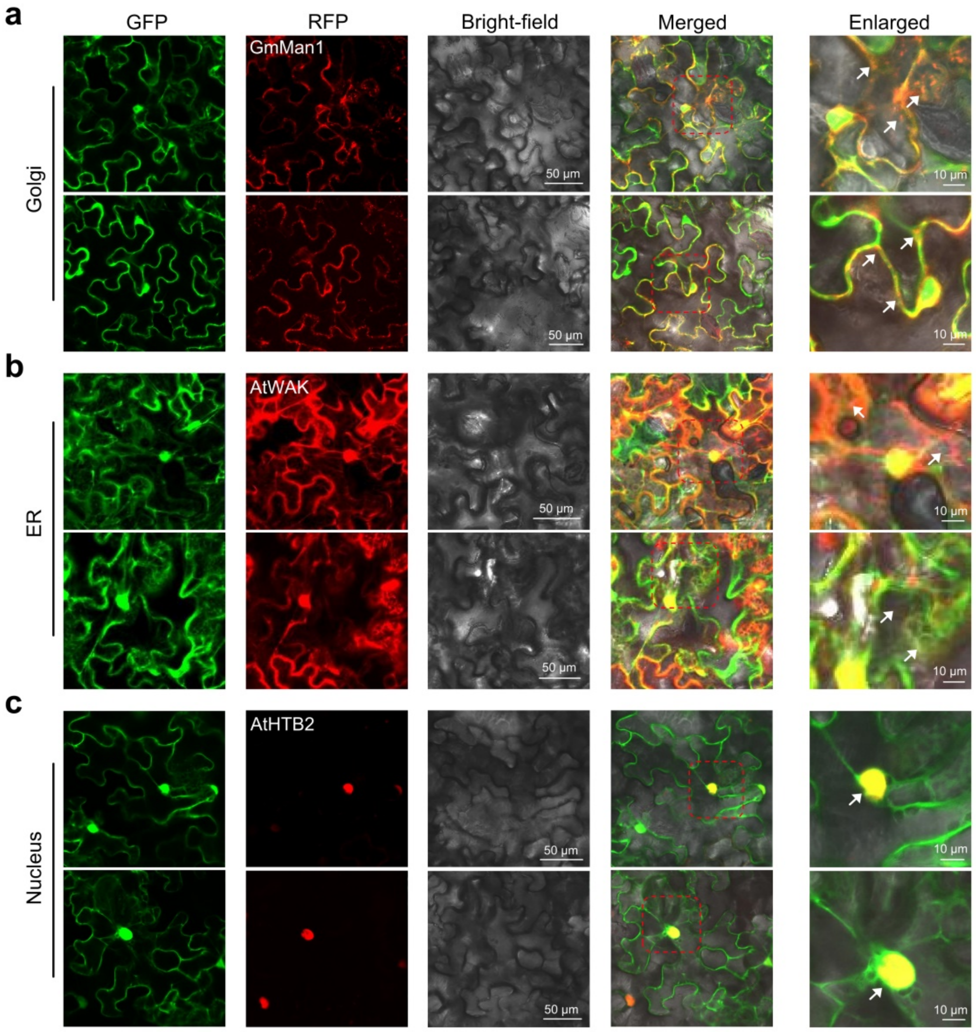

Figure S6 Co-localization analysis of the GFP protein with the Golgi, endoplasmic reticulum and nucleus markers. a) The GFP protein does not co-localize with a Golgi marker (GmMan1, soybean a-1,2-mannosidase I). White arrows indicate small Golgi vesicles. **b)** The GFP protein co-localizes with an endoplasmic reticulum marker (AtWAK, wall-associated kinase 2). White arrows indicate overlapping regions with the endoplasmic reticulum. **c)** The GFP protein co-localizes with a nucleus marker (AtHTB2, histone B2). White arrows indicate GFP green fluorescence was within the nucleus. Images were captured by confocal microscopy (LSM 780; Zeiss). Scale bars: 50 µm and 10 µm.

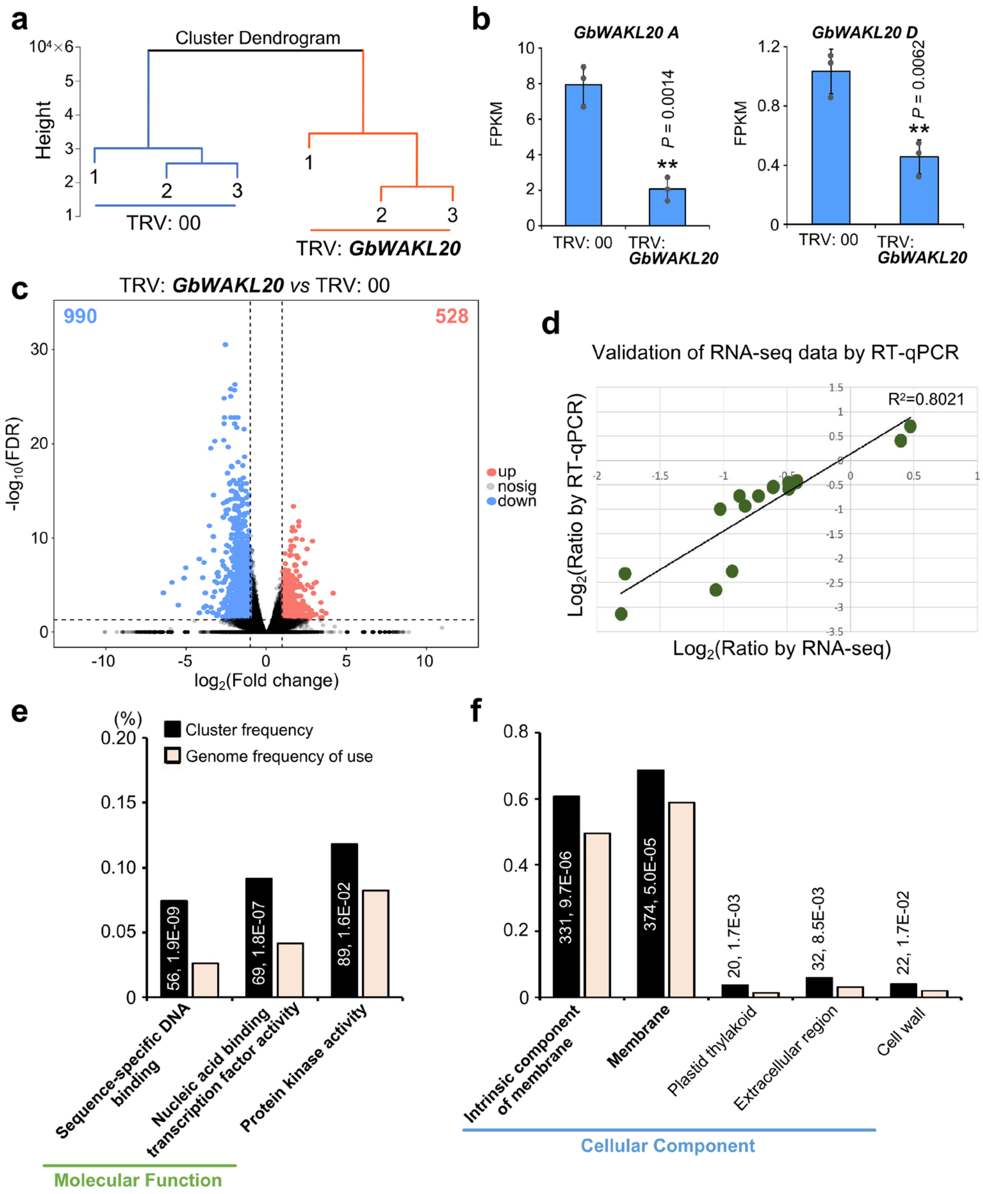

Figure S7 RNA-seq assay of TRV: *GbWAKL20* and TRV: 00 plants. a) Clustering dendrogram of the 6 RNA-seq samples with three biological replicates for each treatment (n=3). Root from 3-week-old TRV: *GbWAKL20* and TRV: 00 plants were sampled for RNA-seq analysis. **b)** Transcripts of *GbWAKL20* from RNA-seq sequencing data were normalized using FPKM. The transcript levels of *GbWAKL20* in TRV: *GbWAKL20* plants were significantly lower than those in TRV: 00 plants. Each treatment included three biological replicates (n=3). There were two homoeologous genes of *WAKL20* from A-subgenome (A) and D-subgenome (D) in tetraploid cotton species. Both were silenced due to the high sequence identity. **c)** Volcano plots of differentially expressed genes (DEGs) in TRV: *GbWAKL20* plants. Genes with an adjusted *q-value*<0.05 and absolute fold change value >2 are designated as DEGs. Blue indicates

down-regulated DEGs and red indicates up-regulated DEGs. **d)** Correlation of fold change analyzed by RNA-seq data was compared with results obtained from RT-qPCR. Expression data were collected from 17 genes for TRV: *GbWAKL20* and TRV: 00 plants. The $\log_2$ (expression ratio) values from RNA-seq were plotted against the $\log_2$ (expression ratio) obtained by RT-qPCR, drew standard curves and calculated the R^2 value to test the fitness. **e-f)** GO enrichment analysis of 1,518 DEGs in TRV: *GbWAKL20* versus TRV: 00 plants. Significantly enriched terms in Molecular Function and Cellular Component categories are shown. The numbers near the columns indicate the number of DEGs with corresponding annotations and the *P. adjust*-value, respectively.

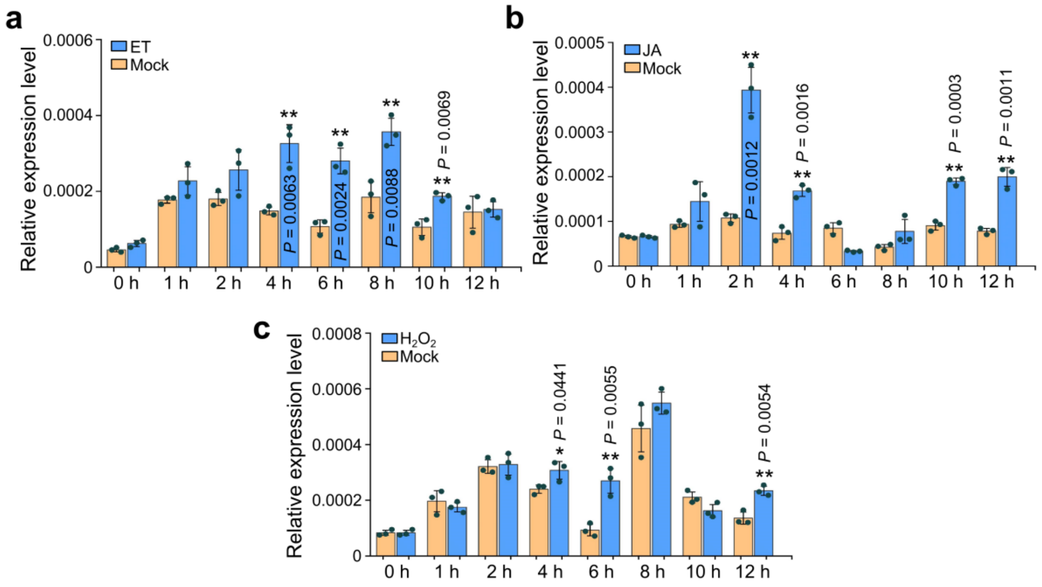

Figure S8 Induced expression analysis of *GbWAKL20* in response to hormone and H_2O_2 signaling molecule treatments in cotton. Comparison of the expression levels of *GbWAKL20* in cotton plants treated with **a)** ET, **b)** JA and **c)** H_2O_2 signal compound and H_2O-treated control plants. Error bars represent the standard deviation of three independent experiments for each experiment (n=3). Asterisks indicate statistically significant differences, as determined by Student's *t*-tests (**P*<0.05, ***P*<0.01).

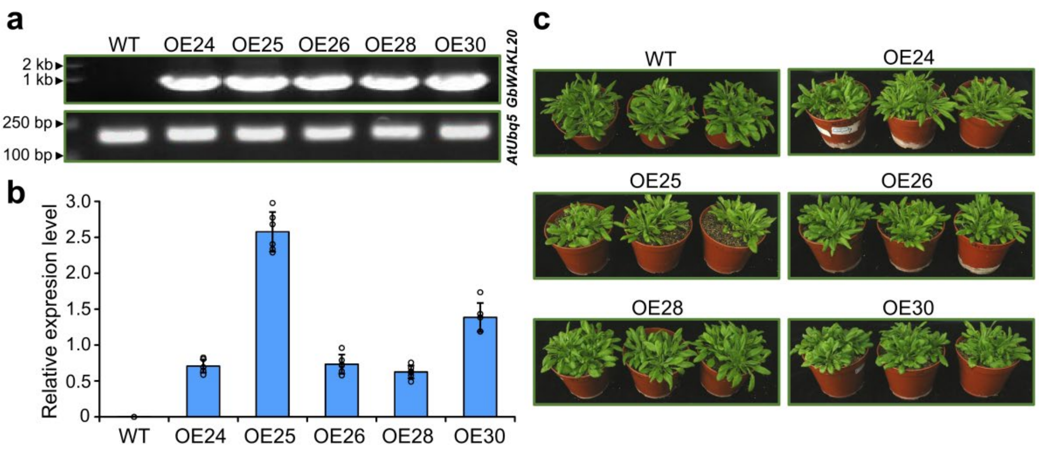

Figure S9 Molecular identification and growth observation of *GbWAKL20*-overexpressing Arabidopsis plants. a) *GbWAKL20* was verified by PCR using genomic DNA in different Arabidopsis transgenic lines, using Arabidopsis *AtUbq5* (*At3g62250*) as the internal control. **b)** *GbWAKL20* transcript levels in each transgenic line was quantified by RT-qPCR using Arabidopsis *AtUbq5* (*At3g62250*) as the internal control. WT, Arabidopsis Columbia-0 (Col-0); OEs represent the different Arabidopsis transgenic lines. Error bars represent the standard deviation of six biological replicates (n=6). **c)** Phenotype observation of the above-ground part of transgenic Arabidopsis lines. After being cultured vertically about 8 days on 1/2 MS solid medium, the seedlings were transferred to vermiculite in a growth chamber. Phenotype observations were performed on 4-weeks-old plants.

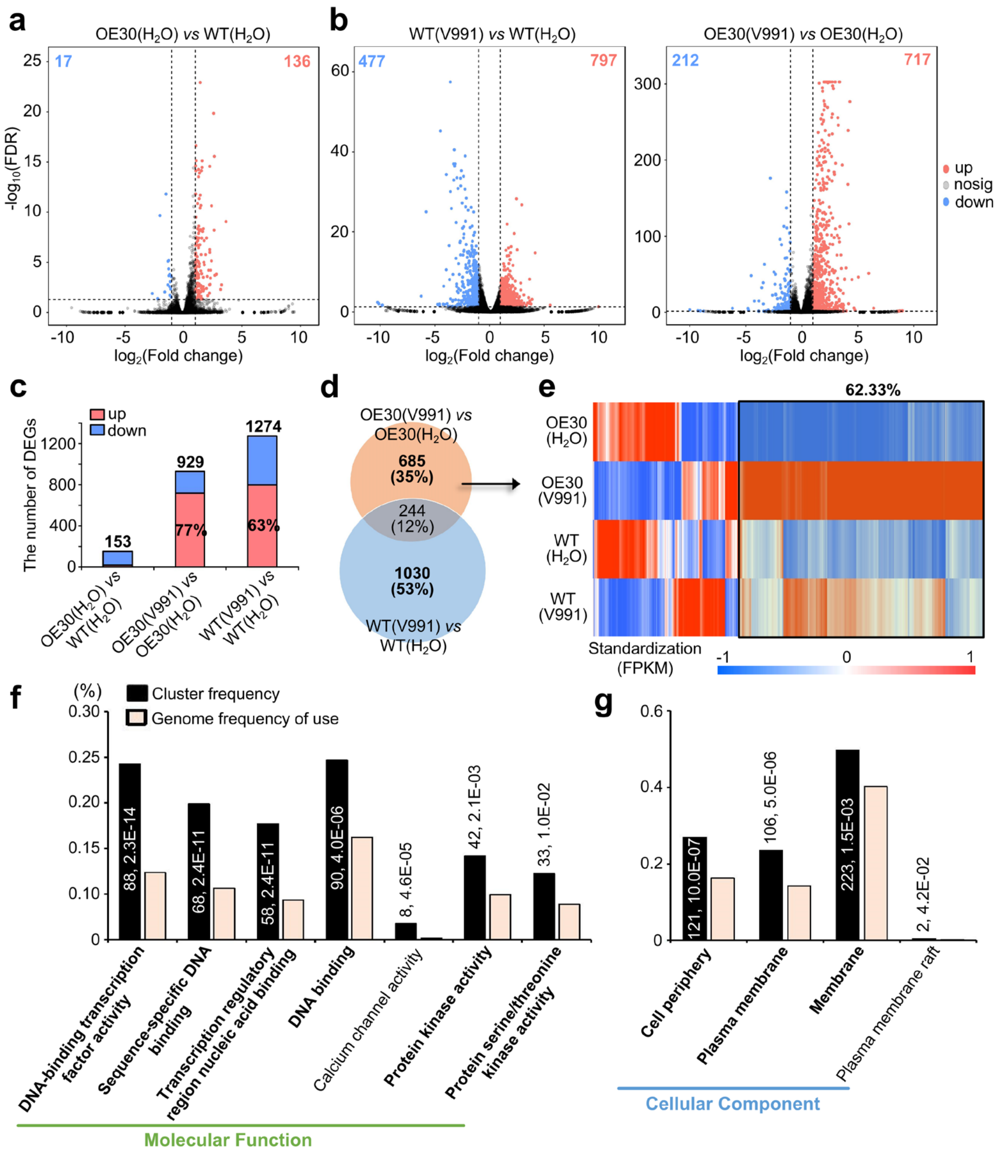

Figure S10 RNA-seq assay of *GbWAKL20*-overexpressing Arabidopsis plants. a) Volcano map of DEGs in *GbWAKL20*-overexpressing plants compared to control plants under water treatment conditions. **b)** Volcanic maps of induced DEGs in *GbWAKL20*-overexpressing plants or control plants after treatment with *Vd*. Genes with an adjusted *q-value*<0.05 and an absolute fold change>2 are designated as DEGs. Blue indicates down-regulated DEGs and red indicates up-regulated DEGs. **c)** The number of DEGs in the roots of *GbWAKL20*-overexpressing OE30 line versus W0 plants after three days of treatment with H_2O or *Vd*. The red represents the number of up-regulated DEGs, while the blue represents the number of down-regulated DEGs in the histogram. **d)** Comparative analysis of the number of DEGs between *GbWAKL20*-overexpressing plants and WT plants after induction by *Vd*. **e)** Heatmaps of 929 DEGs in OE30 plants after treatment with H_2O or *Vd*. The black box shows DEGs that were more strongly induced by *Vd* in

OE30 plants, accounting for 62.33% (579/929) of the total DEGs. The numerical values for the blue-to-red gradient bar represent the FPKM standardization values of the DEGs in each sample. **f-g)** GO enrichment analysis of 579 DEGs in *GbWAKL20*-overexpressing plants. GO terms for DEGs were enriched in Molecular function and Cellular component. A total of 579 DEGs were strongly induced and upregulated after *Vd* inoculation in *GbWAKL20*-overexpressing plants. The numbers near the columns indicate the count of DEGs with their corresponding annotations and the *P. adjus*t-value, respectively.

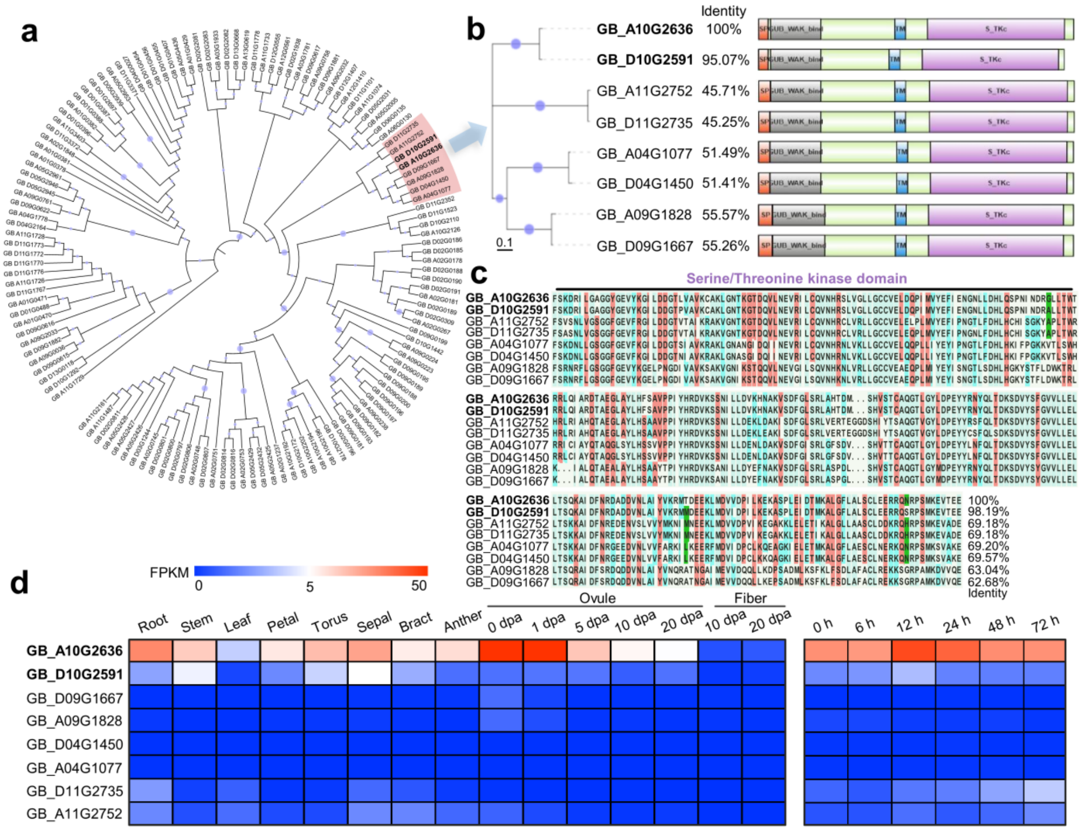

Figure S11 Sequence and expression pattern of GbWAKL20 homologs in cotton. a) Phylogenetic relationship of WAKL family genes in *G. barbadense* acc. Hai7124. The phylogenetic tree was generated using the maximum likelihood method under the WAG model in MEGA v5.1 (http://www.megasoftware.net/), and the reliability of interior branches was assessed with 1000 bootstrap re-samplings. The pink area shows the homologs for WAKL20. **b)** Homology and structural analysis of WAKL20 homologs in cotton. DNAMAN software (http://www.lynnon.com/) was used to compare homology between protein sequences. The domains of GbWAKL20 were analyzed using SMART (http://smart.embl.de) and INTERPROSCAN software (http://www.ebi.ac.uk/interpro/). **c)** Sequence alignment of the Serine/Threonine kinase domain of WAKL20 homologs. Identical amino acid residues are highlighted in blue. **d)** Expression patterns of *GbWAKL20* homologs in various tissues, and their induced expression profiles in response to *Vd* infection. Roots of Hai7124 and TM-1 were sampled for transcriptome analysis at 0, 6, 12, 24, 48 and 72 hours after *Vd* inoculation. The expression data were converted to FPKM to calculate the expression levels of the genes. Each treatment included three biological replicates (n=3). Colored squares indicated expression levels from 0 (blue) to 50 (red).

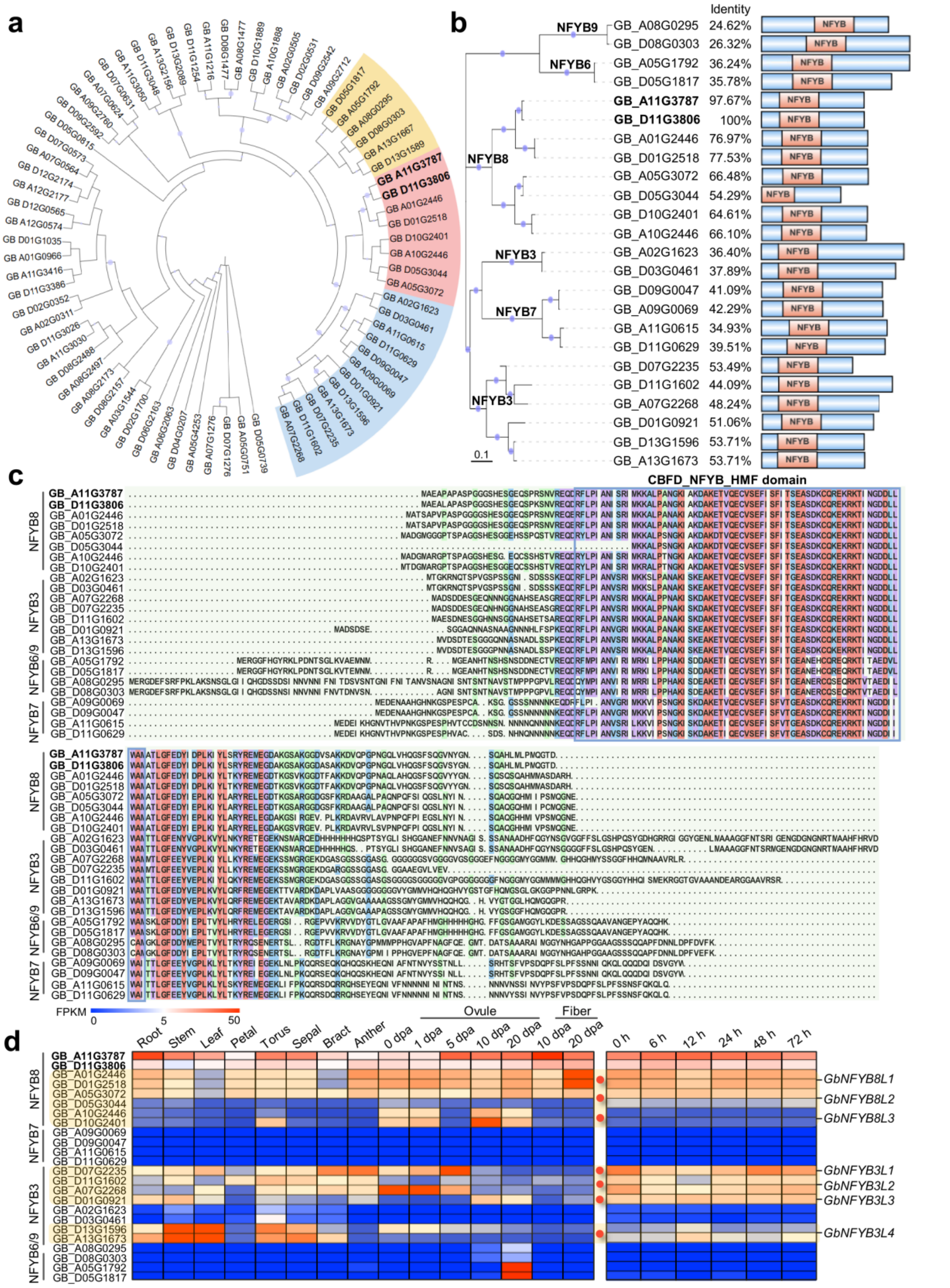

Figure S12 Sequence and expression pattern of GbNFYB8 homologs in cotton. a) Phylogenetic relationship of NFYB family genes in *G. barbadense* acc. Hai7124. The pink area shows the homologs for GbNFYB8. The yellow and blue areas show NFYB3/6/7/9 clustered on the same branch with NFYB8. **b)** Homology and structural analysis of NFYB8 homologs and NFYB3/6/7/9 homologs in cotton. The nomenclature of cotton NFYBs follows as the homology analysis of NFYB genes between cotton and Arabidopsis. The homologous information between

cotton and Arabidopsis was provided in Table S8. **c)** Sequence alignment of NFYB8 homologs and NFYB3/6/7/9 homologs. Identical amino acid residues were highlighted in red. The CBFD_NFYB_HMF domain was labeled with blue box. **d)** Expression patterns of NFYB8 homologs and NFYB3/6/7/9 homologs in various tissues, and their induced expression profiles in response to *Vd* infection. Roots of Hai7124 and TM-1 were sampled for transcriptome analysis at 0, 6, 12, 24, 48 and 72 hours after *Vd* inoculation. The expression data were converted to FPKM to calculate the expression levels of the genes. Each treatment included three biological replicates (n=3). Colored squares indicated expression levels from 0 (blue) to 50 (red).

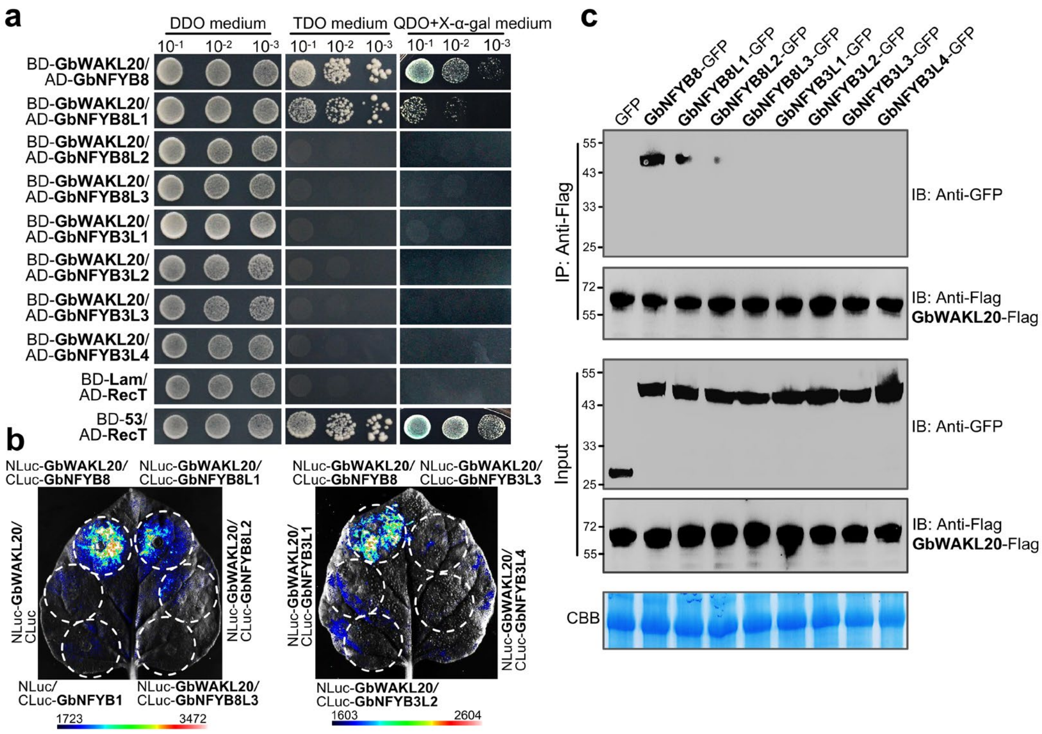

Figure S13 Interaction analysis between GbWAKL20 and GbNFYB8 homologs. a) GbWAKL20 interaction with GbNFYB8 and GbNFYB8L1 in yeast-two-hybrid (Y2H) assay. Yeast cells containing the indicated plasmids were cultured on selective medium (DDO) SD/-Leu/-Trp/, (TDO) SD/-Leu/-Trp/-His, and (QDO) SD/-Leu/-Trp/-His/-Ade in the presence of X-α-gal, positive interactions can grow and turn blue. Yeast cells with a series of concentrations were dotted on the medium to test the growth rate. Interactions of BD-53/AD-RecT and BD-Lam/AD-RecT were used as positive and negative controls, respectively. **b)** Verification of interaction between GbWAKL20 and GbNFYB8 homologs by luciferase complementation imaging (LCI) assays. The Agrobacterium strain GV3101 harboring the indicated plasmid pairs was infiltrated into the leaves of *N. benthamiana* and transiently co-expressed. The luminescent signal was collected 48 hours post-infiltration. **c)** Verification of interaction between GbWAKL20 and GbNFYB8 homologs by Co-IP assays. GbWAKL20-Flag and GbNFYB8 homologs-GFP were co-expressed in leaves of *N. benthamiana*. Extracted proteins were subjected to Co-IP using Flag-trap beads and Western blotting with anti-Flag and anti-GFP, respectively. Total proteins stained with CBB served as a loading control.

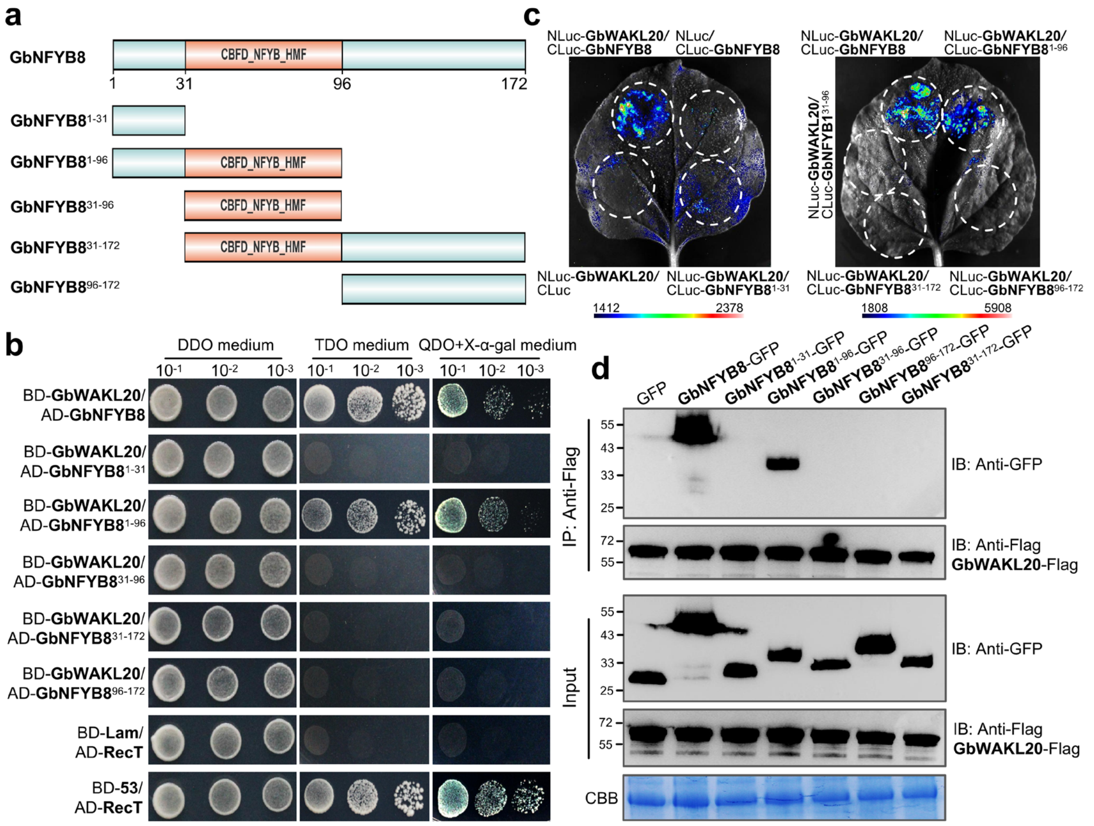

Figure S14 The N-terminal region and the CBFD_NFYB_HMF domain of GbNFYB8 is required for the interaction with GbWAKL20. a) Constructs for identification of the region in GbNFYB8 required for interaction with GbWAKL20. Five truncated versions of GbNFYB8, GbNFYB8$^{1-31}$, GbNFYB8$^{1-96}$, GbNFYB8$^{31-96}$, GbNFYB83$^{1-172}$ and GbNFYB8$^{96-172}$ as well as full-length of GbNFYB8 were used to test the interaction with GbWAKL20. **b)** GbWAKL20 interaction with GbNFYB8$^{1-96}$ in yeast-two-hybrid (Y2H) assay. Yeast cells containing the indicated plasmids were cultured on selective medium DDO (SD/-Leu/-Trp), TDO (SD/-Leu/-Trp/-His), and QDO (SD/-Leu/-Trp/-His/-Ade) in the presence of X-α-gal. Positive interactions can grow and turn blue in QDO medium. Yeast cells with a series of concentrations were dotted on the medium to test the growth rate. Interactions of BD-53/AD-RecT and BD-Lam/AD-RecT were used as positive and negative controls, respectively. **c)** Verification of GbWAKL20 and GbNFYB8$^{1-96}$ interaction by luciferase complementation imaging (LCI) assays. The Agrobacterium strain GV3101 harboring the indicated plasmid pairs was infiltrated into the leaves of *N. benthamiana* and transiently co-expressed. The luminescent signal was collected 48 hours post-infiltration. **d)** Verification of GbWAKL20 and GbNFYB8$^{1-96}$ interaction by Co-IP assays. GbWAKL20-Flag and five truncated versions of GbNFYB8 were co-expressed in leaves

of *N. benthamiana*. Extracted proteins were subjected to Co-IP using Flag-trap beads and Western blotting with anti-Flag and anti-GFP, respectively. Total proteins stained with CBB served as a loading control.

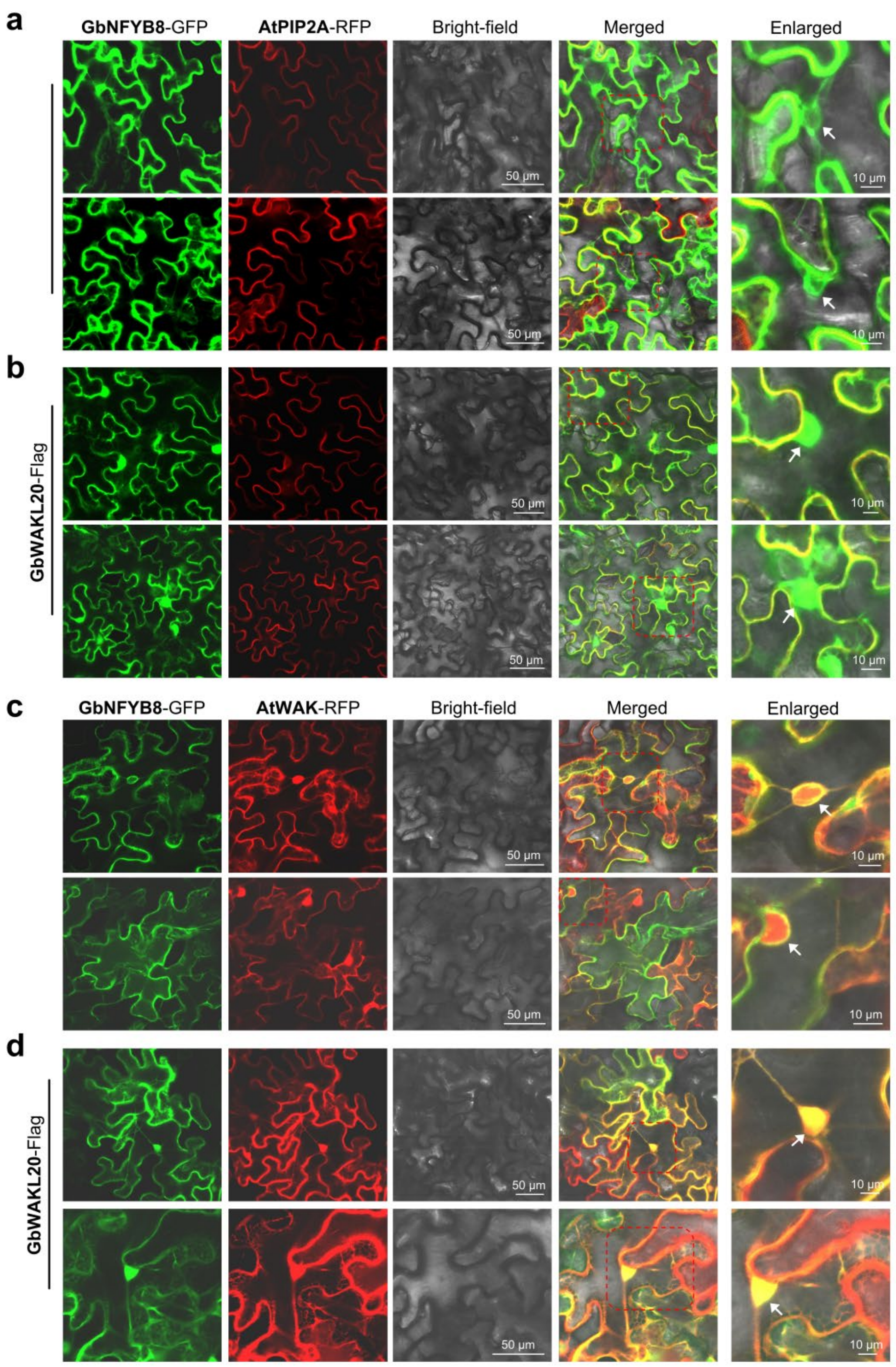

Figure S15 Subcellular localization of GbNFYB8 in *N. benthamiana* epidermal cells. a) The GbNFYB8-GFP fusion protein co-localizes with a plasma membrane marker (AtPIP2A-RFP, a plasma membrane aquaporin). White arrow indicates GbNFYB8-GFP fluorescence is mostly distributed around the nucleus, with a small amount distributed within the nucleus. **b)** Based on a), expression of GbWAKL20-Flag promotes the translocation of GbNFYB8-GFP fluorescence into the nucleus. White arrow indicates the accumulation of GbNFYB8-GFP fluorescence is in the nucleus. **c)** The GbNFYB8-GFP fusion co-localizes with an endoplasmic reticulum marker

(AtWAK, wall-associated kinase 2). White arrow indicates GbNFYB8-GFP fluorescence is mostly distributed around the nucleus, with a small amount distributed within the nucleus. **d)** Based on c), expression of GbWAKL20-Flag promotes the translocation of GbNFYB8-GFP fluorescence into the nucleus. White arrow indicates the accumulation of GbNFYB8-GFP fluorescence is in the nucleus. Scale bars: 50 μm and 10 μm.

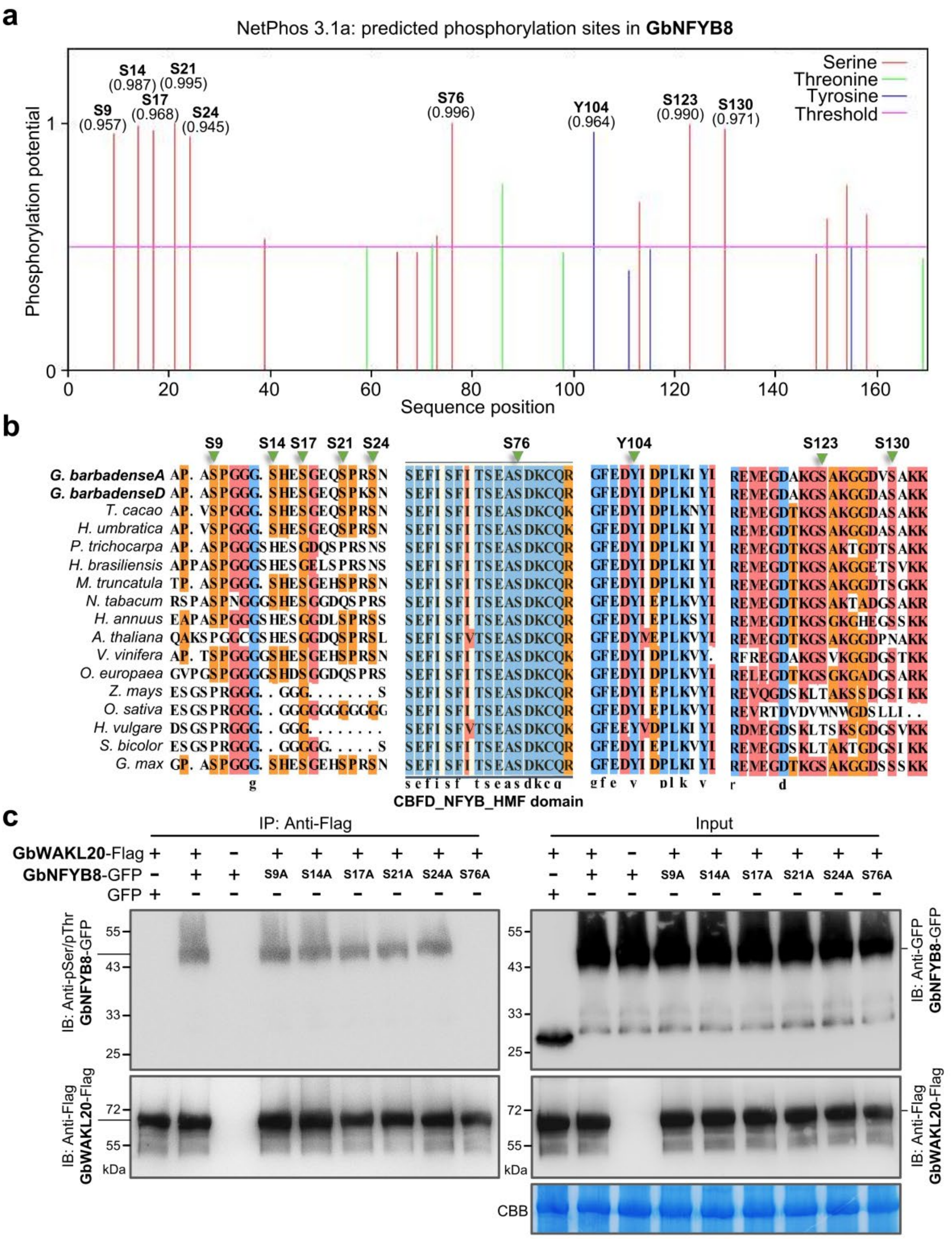

Figure S16 GbWAKL20 phosphorylates GbNFYB8 at Ser-76 site. a) Phosphorylation sites predictions for GbNFYB8 by NetPhos 3.1 (http://www.cbs.dtu.dk/services/NetPhos-3.1/). Score for serine (S) and tyrosine (Y) residues are plotted against their sequence positions; scores >0.9 indicate predicted phosphorylation sites. **b)** Conservation analysis of NFYB8 phosphorylation sites across plant species. Green arrows mark predicted phosphorylation sites; blue shading denotes conserved residues. The Ser-76 site (within the CBFD_NFYB_HMF domain) is a highly conserved putative phosphorylation site. **c)** Mutation of Ser76 in GbNFYB8 blocked its phosphorylation by GbWAKL20 in *N. benthamiana*.

GbWAKL20-Flag and GbNFYB8-GFP were co-expressed in the leaves of *N. benthamiana*. Total proteins stained with CBB served as a loading control. Serine residues at positions S9, S14, S17, S21, S24 and S76 of GbNFYB8 were individually mutated to Alanine, respectively. The GbNFYB8-GFP or its mutant proteins were immunoprecipitated using an anti-Flag antibody, and detected by immunoblotting with anti-pSer/pThr and anti-GFP antibodies. GbWAKL20-Flag protein was detected by immunoblotting with an anti-Flag antibody.

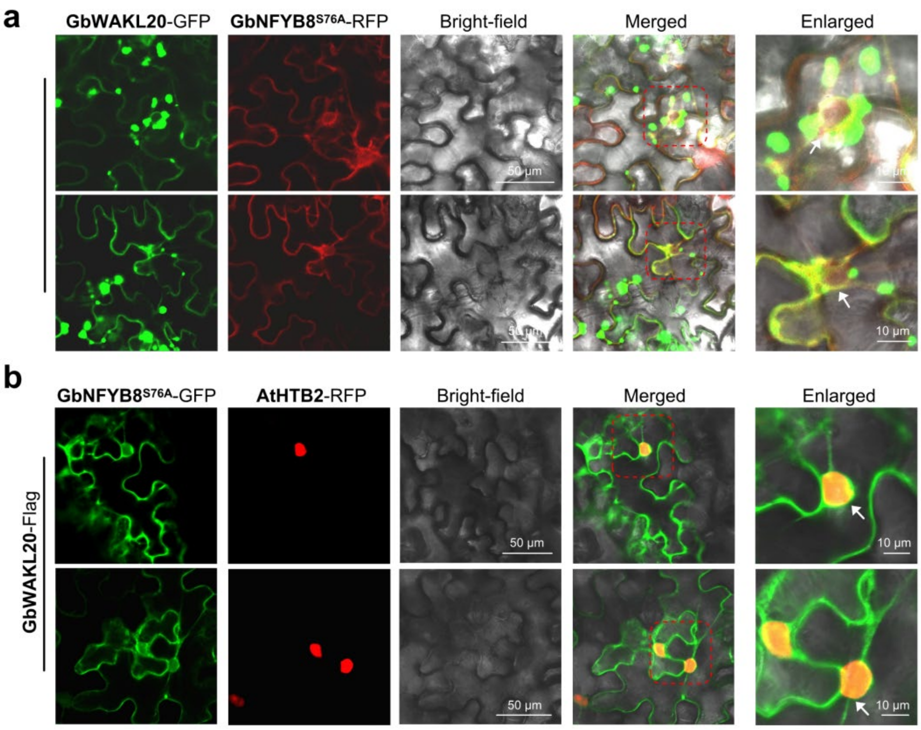

Figure S17 The mutation of Ser-76 prevents GbNFYB8 from being phosphorylated by GbWAKL20 and translocated to the nucleus. a) Co-localization of GbWAKL20-GFP and GbNFYB8^{S76A}-RFP in *N. benthamiana* leaves. White arrows indicate that both GbWAKL20-GFP and GbNFYB8-RFP distributed around the nucleus. **b)** When the Serine residue at position 76 of GbNFYB8 mutated to Alanine, the expression of GbWAKL20-Flag in *N. benthamiana* fails to promote nuclear translocation of GbNFYB8^{S76A}-GFP fluorescence. White arrows indicate that the GbNFYB8-GFP^{S76A} fluorescence is mostly distributed around the nucleus, with a small amount distributed within the nucleus. Scale bars: 50 μm and 10 μm.

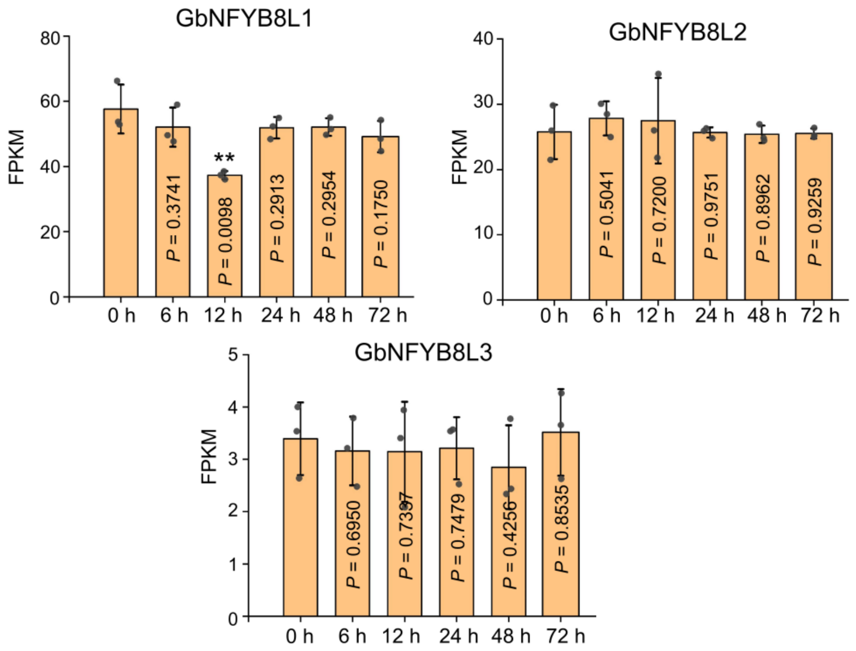

Figure S18 Induced expression analysis of *GbNFYB8* homologs in response to *Vd* in cotton.

The Hai7124 roots were sampled for transcriptome analysis at 0, 6, 12, 24, 48 and 72 hours after *Vd* inoculation. The expression data were represented as FPKM values, which were used to calculate the expression levels of *GbNFYB8L1-3*. Error bars represent the standard deviation of three independent experiments for each experiment (n=3). Asterisks indicate statistically significant differences, as determined by Student's *t*-tests (***P*<0.01).

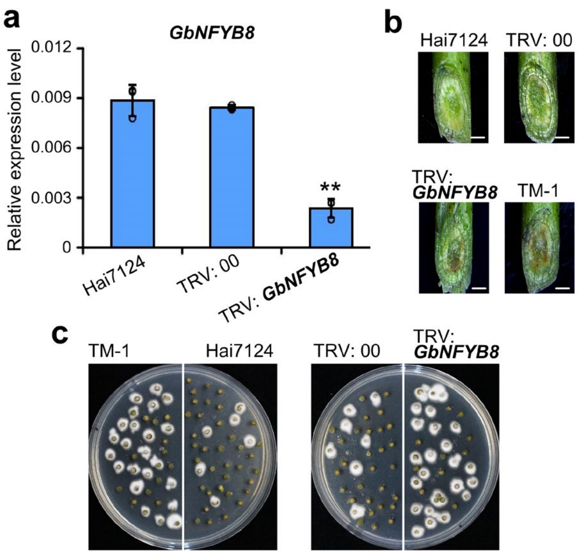

Figure S19 Silencing *GbNFYB8* in *G. barbadense* acc. Hai7124 leads to decreased resistance to *Vd*. a) *GbNFYB8* transcript levels was detected using RT-qPCR in *GbNFYB8*-silenced and control plants. Error bars represent the standard deviation of three biological replicates (n=3). Statistical analyses were performed using Student's *t*-test (***P*<0.01). **b)** Vascular discoloration was observed in *GbNFYB8*-silenced plants compared to the control plants after inoculation with *Vd*. Photographs were taken with a stereoscope (Olympus MVX10, Tokyo, Japan) 15 days post-inoculation. Scale bars: 1.5 mm. **c)** Fungal recovery experiments. Stem sections of *GbNFYB8*-silenced and control plants at 15 days post-inoculation were cut and placed on potato dextrose agar plates and incubated at 25°C. Photographs were taken at 3 days after culture.

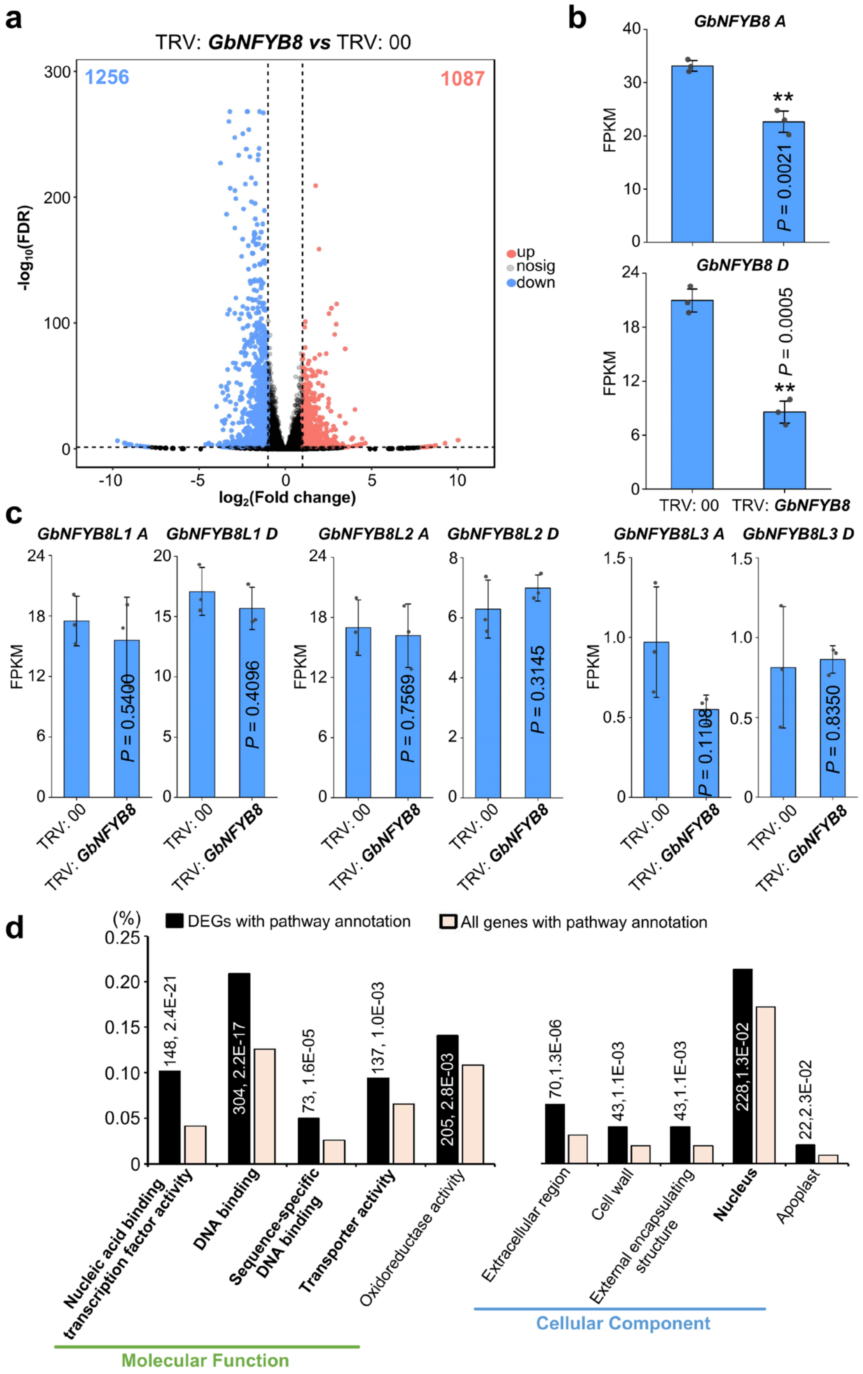

Figure S20 RNA-seq assay of TRV: *GbNFYB8* and TRV: 00 plants. a) Volcano plots of differentially expressed genes (DEGs) in TRV: *GbNFYB8* plants. Genes with an adjusted *q-value* <0.05 and an absolute fold change >2 are designated as DEGs. Blue indicates down-regulated DEGs and red indicates up-regulated DEGs. Root samples from 3-week-old TRV: *GbNFYB8* and TRV: 00 plants were taken for RNA-seq analysis. **b)** Transcripts of *GbNFYB8* in RNA-seq sequencing data, which were normalized by FPKM. The transcripts level of *GbNFYB8* in TRV: *GbNFYB8* plants were significantly lower than those in TRV: 00 plants. Error bars represent the

standard deviation of three biological replicates (n=3). **c)** Transcripts of *GbNFYB8* homologs in RNA-seq sequencing data, which were normalized by FPKM. There was no obvious change in the expression level of *GbNFYB8L1-3* in TRV: *GbNFYB8* and TRV: 00 plants. Error bars represent the standard deviation of three biological replicates (n=3). **d)** GO enrichment analysis of 2,343 DEGs in TRV: *GbNFYB8* versus TRV:00 plants. Significantly enriched terms in Molecular Function and Cellular Component categories are shown. The numbers near the columns indicate the number of DEGs with corresponding annotations and the *P.adjust*-value, respectively.

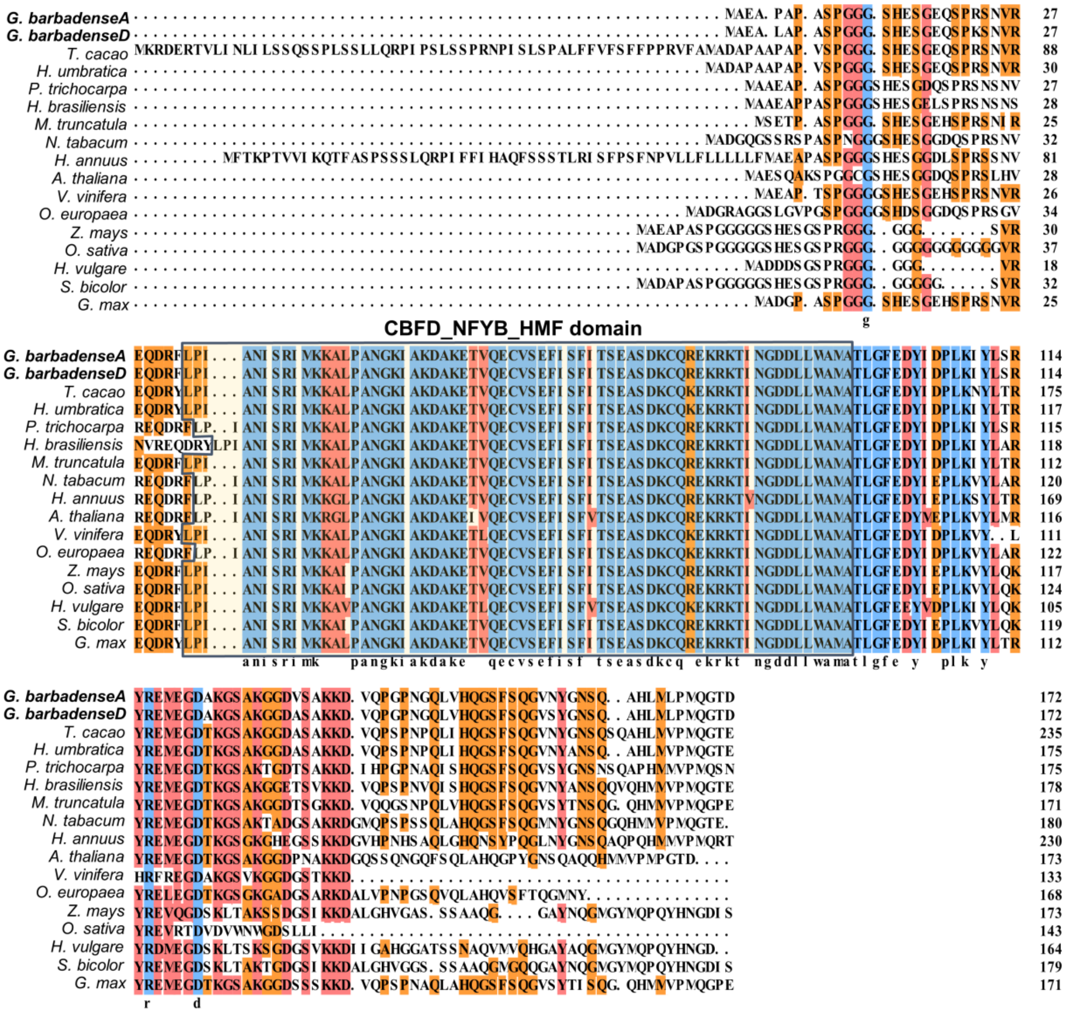

Figure S21 Sequence alignment of NFYB8 homologs from different plant species. Amino acid sequence alignment of NFYB8 homologs in *Gossypium barbadense*, *Theobroma cacao*, *Herrania umbratica*, *Populus trichocarpa*, *Hevea brasiliensis*, *Medicago truncatula*, *Nicotiana tabacum*, *Helianthus annuus*, *Arabidopsis thaliana*, *Vitis vinifera*, *Olea europaea*, *Zea mays*, *Oryza sativa*, *Hordeum vulgare*, *Sorghum bicolor*, and *Glycine max* were performed using software DNAMAN. Identical amino acid residues were highlighted in blue. The CBFD_NFYB_HMF domain was labeled with black box.

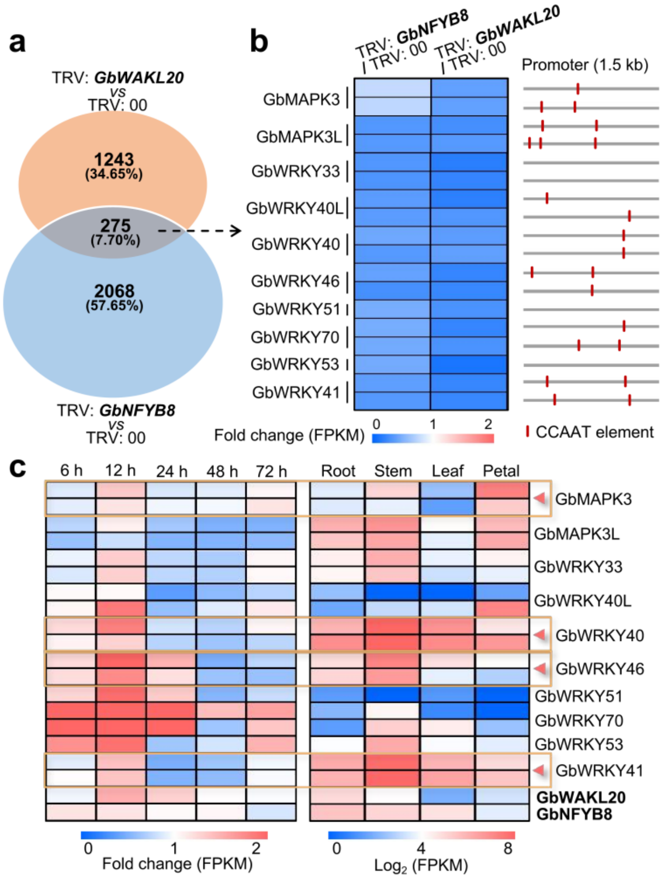

Figure S22 Analysis of potential downstream genes regulated by GbNFYB8. a) Venn diagrams showed the number of common DEGs in *GbWAKL20*-silenced and *GbNFYB8*-silenced plants. **b)** The 275 common DEGs in the a) include *GbMAPK3* and many WRKY transcription factors. Several of these genes contain CCAAT-binding elements within the 1.5 kb upstream promoter region. **c)** Analysis of the expression patterns of *GbMAPK3-1* and WRKY transcription factors in cotton. Roots of Hai7124 and TM-1 were sampled for transcriptome analysis at 0, 6, 12, 24, 48 and 72 hours after *Vd* inoculation. The expression data represent the fold change compared to 0 hours and log_2(FPKM). Each treatment included three biological replicates (n=3). Expression patterns were visualized using MeV 4.7.0. The yellow box showed that *GbMAPK3-1*, *GbWRKY40*, *GbWRKY41* and *GbWRKY46* were highly expressed in both roots and stems, and were significantly up-regulated after *Vd* inoculation, with an expression pattern similar with that of *GbWAKL20* and *GbNFYB8*.

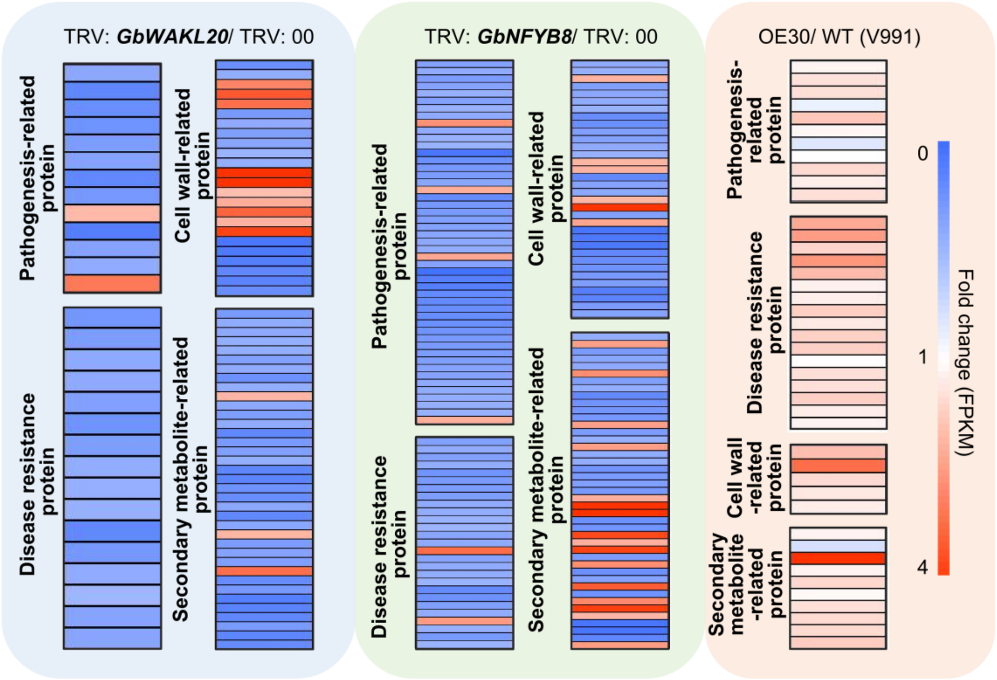

Figure S23 Heatmap showing the differential expression of immune-related genes in the TRV: *GbWAKL20* and TRV: *GbNFYB8* cotton plants, and *GbWAKL20*-overexpressing Arabidopsis, compared to control plants. These genes encode proteins related to pathogenesis, disease resistance, cell wall and secondary metabolism. The numerical values for the blue-to-red gradient bar represent the multiples of differences in the FPKM values of the DEGs in each sample. Each treatment included three biological replicates (n=3).

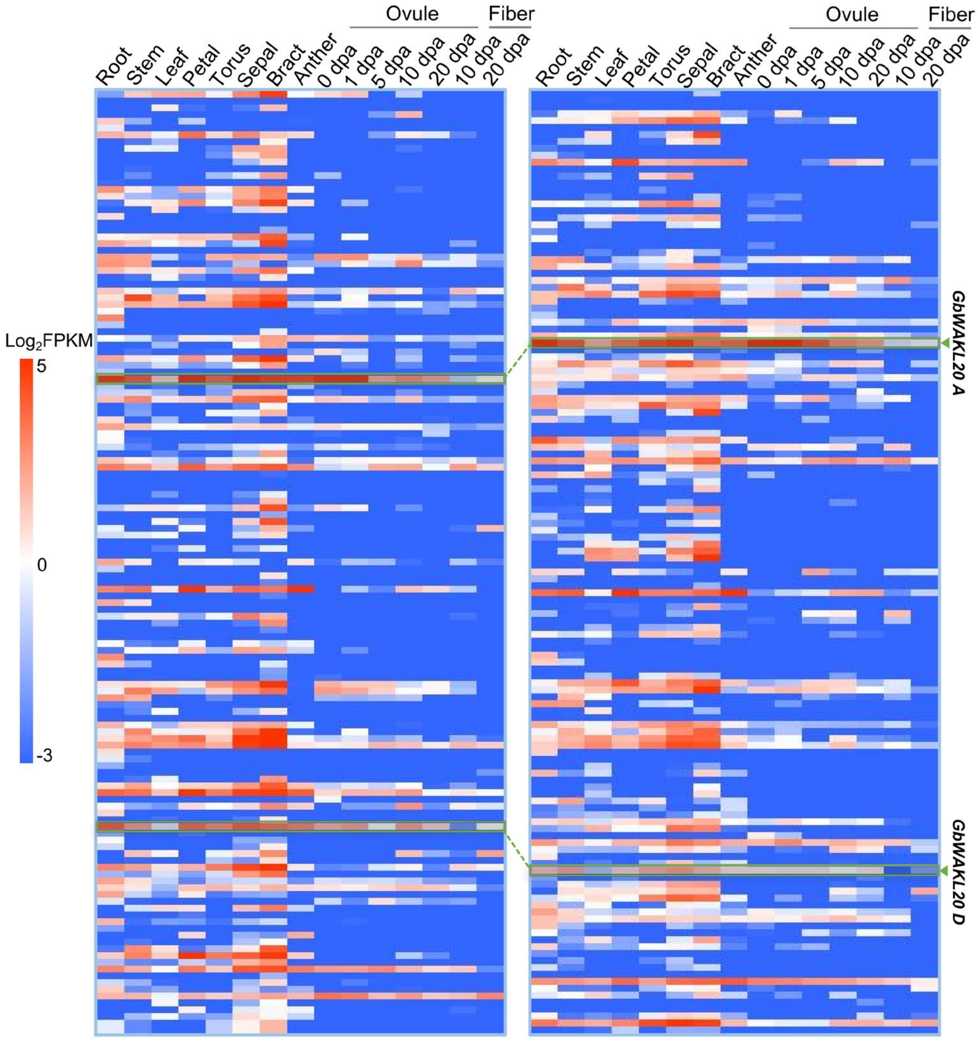

Figure S24 Transcriptional profiling of WAKs/WAKLs in different tissues and organs in *G. hirsutum* acc. TM-1 and *G. barbadense* acc. Hai7124. Root, stem, leaf, petal, sepal, bract, anther, ovules at 0, 1, 5, 10, 20 days post anthesis (dpa), fiber at 10 and 20 dpa were used for the comparative transcriptome analysis. The expression profile of *WAKL20* were displayed in the green box. Each treatment included three biological replicates (n=3). The RNA-seq data were from http://www.ncbi.nlm.nih.gov/bioproject/503814. left: TM-1; right: Hai7124.

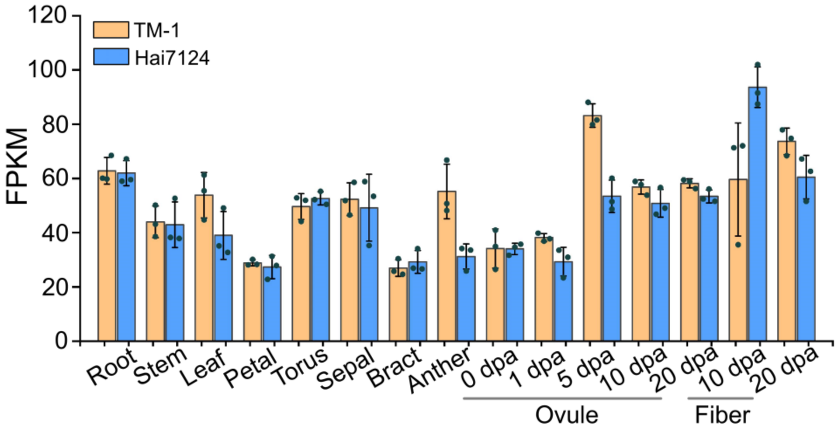

Figure S25 Expression pattern of *GbNFYB8*/*GhNFYB8* in various tissues in *G. barbadense* acc. Hai7124 and *G. hirsutum* acc. TM-1. The expression data were converted to FPKM to calculate the expression levels of *GbNFYB8*/*GhNFYB8*. Error bars represent the standard deviation of three biological replicates (n=3). The RNA-seq data were from http://www.ncbi.nlm.nih.gov/bioproject/503814. Root, stem, leaf, petal, sepal, bract, anther, ovules at 0, 1, 5, 10, 20 days post anthesis (dpa), and fibers at 10 and 20 dpa were used for expression analysis.

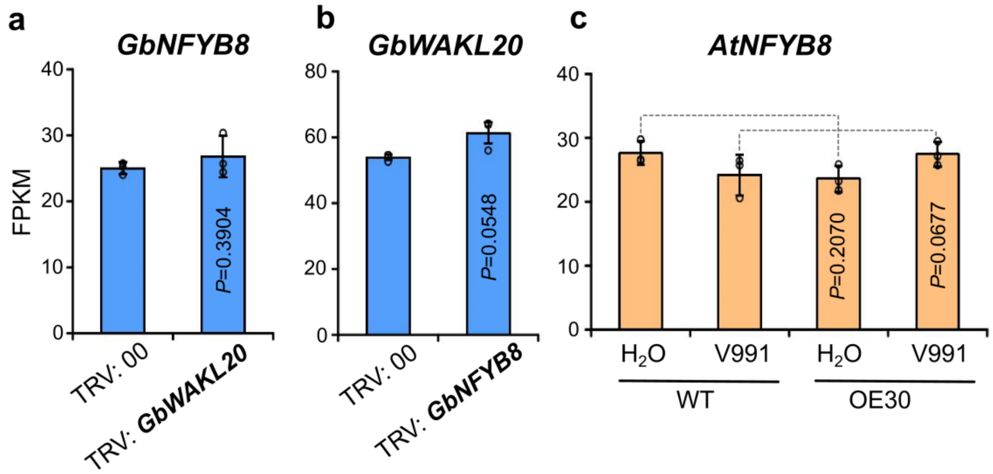

Figure S26 *GbNFYB8* and *GbWAKL20* do not affect each other on transcription levels. a) There was no obvious change of *GbNFYB8* transcription level in *GbWAKL20*-silenced and control plants. **b)** There was no obvious change of *GbWAKL20* transcription level in *GbNFYB8*-silenced and control plants. **c)** There was no obvious change of *AtNFYB8* transcription level in *GbWAKL20*-overexpressing transgenic Arabidopsis and control plants whether inoculation or non-inoculation by *Vd*. Error bars represent the standard deviation of three biological replicates (n=3).

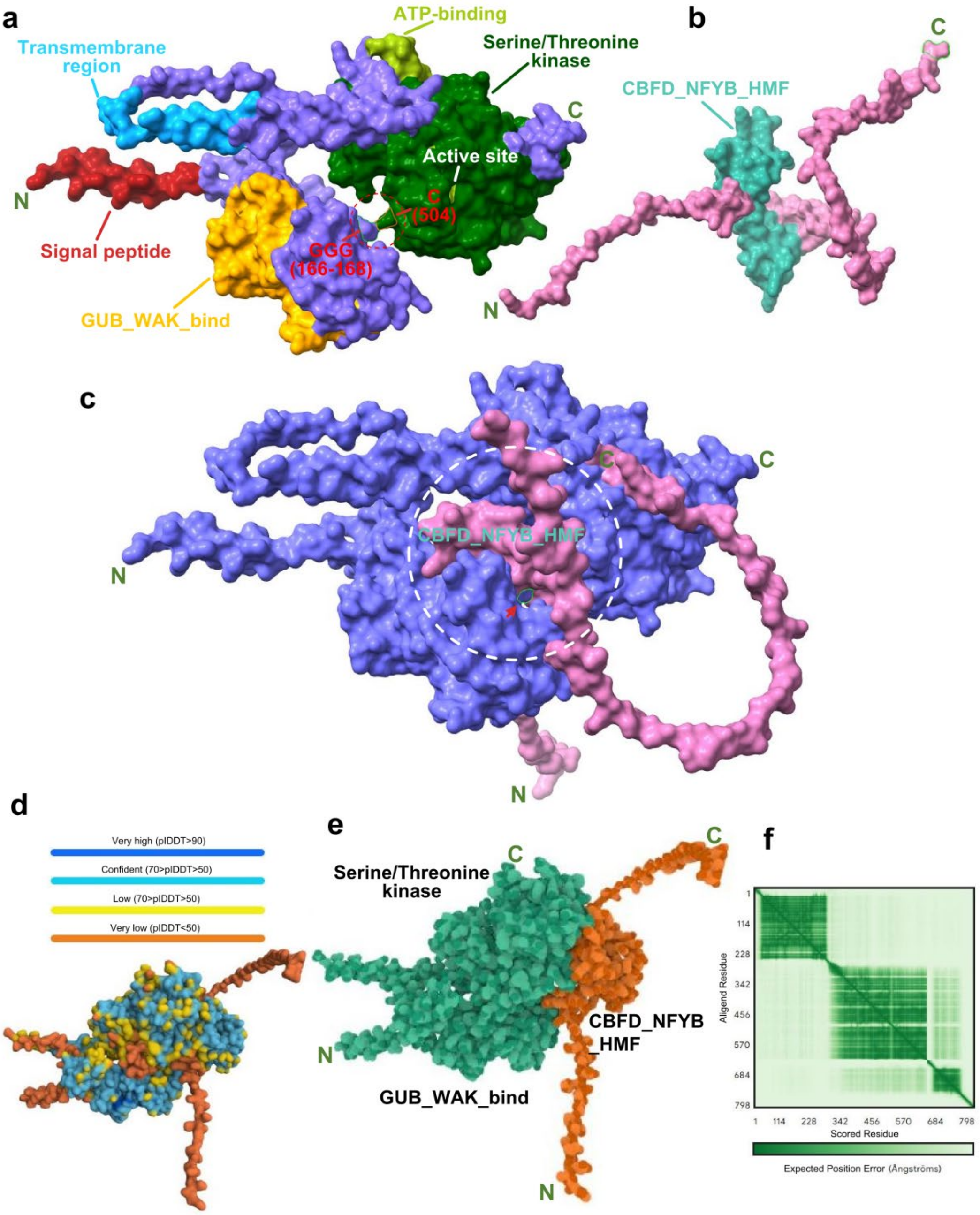

Figure S27 Protein structure analysis of GbWAKL20 and GbNFYB8 at the 3D level. a-b) The predicted structures of GbWAKL20 (PDB ID: C6ZRT3) and GbNFYB8 (PDB ID: A0A2P5XF23) are displayed as a diagram and surface views using UCSF Chimera software. The red arrows indicate a possible hairpin structure formed by the GUB_WAK_bind and Serine/Threonine kinase domains. **c)** Models of GbWAKL20 with GbNFYB8 interaction predicted using UCSF Chimera software. The white circle is labeled as GbWAKL20 interacts with the CBFD_NFYB_HMF domain of GbNFYB8. The red arrow indicates that GbWAKL20 may be

stuck in the CBFD_NFYB_HMF domain through the hairpin structure. **d)** Models of GbWAKL20 with GbNFYB8 interaction predicted using AlphaFold 3. The predicted local distance difference test (pLDDT) scores range from 0 to 100, where $\text{pLDDT}\geqslant 90$ indicates residues predicted with very high confidence. Residues with $90>\text{pLDDT}\geqslant 70$ are classified as confident, while those with $70>\text{pLDDT}\geqslant 50$ are predicted with low confidence. Values below 50 indicate extremely low confidence, suggesting that accurate prediction may not be possible. **e)** The GUB_WAK_bind domain of GbWAKL20 interacts with the CBFD_NFYB_HMF domain of GbNFYB8. **f)** Estimate of the error in the relative position and orientation between two tokens in the predicted structure. Higher values indicate higher predicted error and therefore lower confidence.
